# Supplementary material for: Comparison of IMU-Based Knee Kinematics with and without Harness Fixation against an Optical Marker-Based System
Source: Bioengineering (Basel). 2024 Sep 28;11(10):976. doi: 10.3390/bioengineering11100976 (PMC11505205; doi:10.3390/bioengineering11100976)
Supplement: Supplementary file 1 [file bioengineering-11-00976-s001.zip › bioengineering-3158722-supplementary.pdf]

Supplementary Materials: Comparison of IMU-Based Knee Kinematics with and without Harness Fixation against an Optical Marker-Based System

Jana G. Weber [1,†], Ariana Ortigas-Vásquez [1,2,†], Adrian Sauer [1,*], Ingrid Dupraz [1], Michael Utz [1], Allan Maas [1,2] and Thomas M. Grupp [1,2]

[1] Research and Development, Aesculap AG, 78532 Tuttlingen, Germany;

[2] Department of Orthopaedic and Trauma Surgery, Musculoskeletal University Center Munich (MUM), Campus Grosshadern, Ludwig Maximilians University Munich, 81377 Munich, Germany

[*] Corresponding Author: adrian.sauer@aesculap.de

† These authors contributed equally to this work.

1 Averaged over all knees and cycles

	Femur		Tibia	
	IMUs on harness	IMUs on skin	IMUs on harness	IMUs on skin
Rx	0.0	0.0	11.1	12.7
Ry	8.0	15.6	8.2	16.4
Rz	19.8	25.1	25.8	31.0

Table S1: Absolute maximum (in degrees) of the rotational transformations (Rx: Rotation around X, Ry: Rotation around Y, Rz: Rotation around Z) applied to the femoral and tibial segment frames as part of REFRAME$_{IMU->OMC}$, calculated across all knees and cycles.

	Femur			Tibia		
	OMC on harness	IMUs on harness	IMUs on skin	OMC on harness	IMUs on harness	IMUs on skin
Rx	0.0	0.0	0.0	18.4	21.1	20.6
Ry	16.0	14.7	32.7	13.0	14.2	31.9
Rz	22.7	15.3	26.8	23.1	16.8	24.4

Table S2: Absolute maximum (in degrees) of the rotational transformations (Rx: Rotation around X, Ry: Rotation around Y, Rz: Rotation around Z) applied to the femoral and tibial segment frames as part of REFRAME$_{RMS}$, calculated across all knees and cycles.

	**Raw**		**REFRAME$_{IMU->OMC}$**		**REFRAME$_{RMS}$**	
	IMUs on harness	IMUs on skin	IMUs on harness	IMUs on skin	IMUs on harness	IMUs on skin
flexion/extension	11.3	12.7	3.1	3.9	3.5	6.5
abduction/adduction	11.7	10.5	1.6	3.3	1.9	3.9
external/internal rotation	11.6	13.9	2.1	7.4	2.1	7.3

Table S3: Maximum (in degrees) root-mean-square errors between tibio-femoral joint rotations estimated by the IMU-based systems and the optical reference on harness, calculated across all knees and cycles.

2 Averaged plots for individual knees

2.1 Averaged plots for individual knee 1

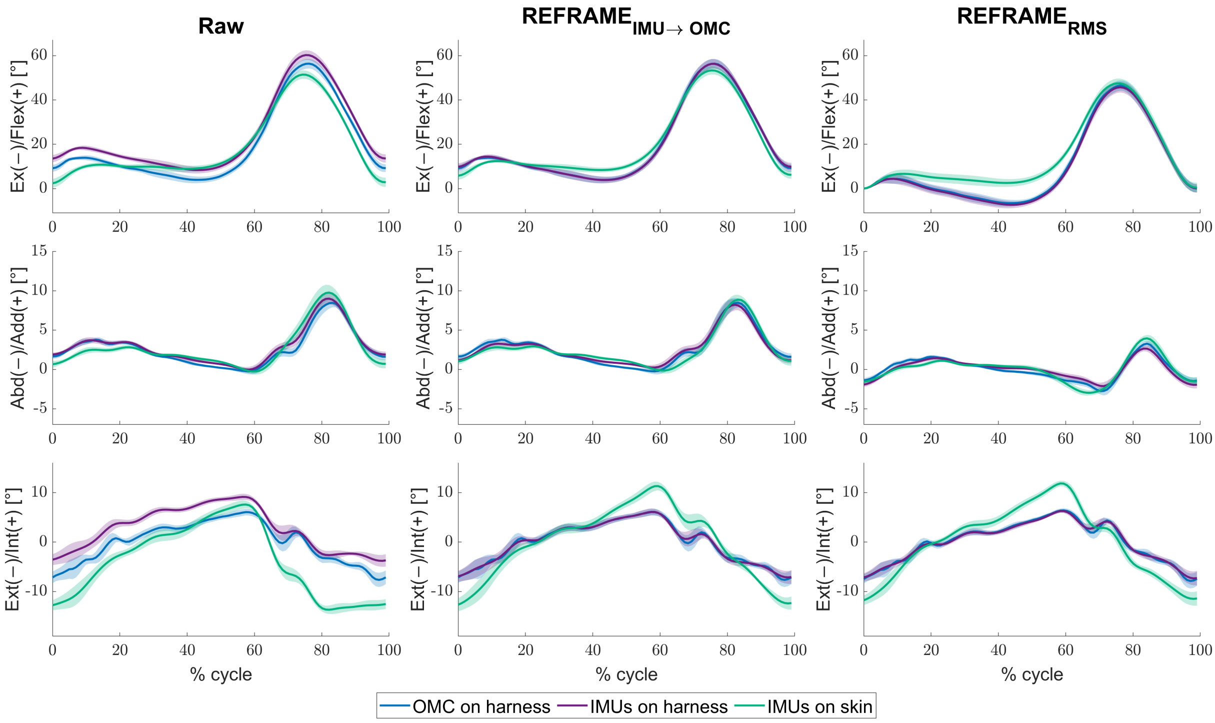

Figure S1: Mean tibio-femoral joint angles (solid lines) ± standard deviation (shaded areas), in degrees, as estimated by inertial measurement units (IMUs) on harness (purple), IMUs on skin (green), and optical motion capture (OMC) on harness (blue), averaged over all cycles for knee 1. Angles are shown as a percentage of the gait cycle under three conditions: 1) raw, i.e. in the absence of post-processing methods to correct reference frame orientation differences (left), 2) after implementation of REFRAME$_{IMU->OMC}$ (middle), and 3) after implementation of REFRAME$_{RMS}$ (right).

	Femur		Tibia	
	IMUs on harness	IMUs on skin	IMUs on harness	IMUs on skin
Rx	0.0 ± 0.0	0.0 ± 0.0	4.0 ± 0.1	-0.5 ± 0.2
Ry	-3.4 ± 0.2	-13.4 ± 1.1	-4.2 ± 0.2	-13.9 ± 1.2
Rz	1.7 ± 0.1	4.1 ± 0.8	-2.1 ± 0.1	3.2 ± 0.3

Table S4: Mean ± standard deviation (in degrees) of the rotational transformations (Rx: Rotation around X, Ry: Rotation around Y, Rz: Rotation around Z) applied to the femoral and tibial segment frames as part of $\text{REFRAME}_{IMU->OMC}$, calculated across all cycles of knee 1.

	Femur			Tibia		
	OMC on harness	IMUs on harness	IMUs on skin	OMC on harness	IMUs on harness	IMUs on skin
Rx	0.0 ± 0.0	0.0 ± 0.0	0.0 ± 0.0	10.1 ± 1.5	14.4 ± 1.6	5.6 ± 2.0
Ry	-5.5 ± 0.9	-9.3 ± 0.9	-12.6 ± 1.6	-7.3 ± 1.0	-12.2 ± 1.0	-14.0 ± 1.6
Rz	-2.9 ± 0.8	-1.1 ± 0.7	-1.4 ± 1.1	-3.0 ± 1.0	-4.2 ± 0.9	0.0 ± 0.9

Table S5: Mean ± standard deviation (in degrees) of the rotational transformations (Rx: Rotation around X, Ry: Rotation around Y, Rz: Rotation around Z) applied to the femoral and tibial segment frames as part of REFRAME_{RMS}, calculated across all cycles of knee 1.

	**Raw**		**REFRAME**$_{IMU->OMC}$		**REFRAME**$_{RMS}$	
	IMUs on harness	IMUs on skin	IMUs on harness	IMUs on skin	IMUs on harness	IMUs on skin
flexion/extension	4.2 ± 0.1	4.8 ± 0.2	0.6 ± 0.1	3.0 ± 0.3	0.8 ± 0.1	5.6 ± 0.5
abduction/adduction	0.5 ± 0.0	1.0 ± 0.2	0.4 ± 0.0	0.6 ± 0.1	0.4 ± 0.0	0.6 ± 0.1
external/internal rotation	3.0 ± 0.1	5.4 ± 0.4	0.4 ± 0.0	3.2 ± 0.3	0.4 ± 0.0	3.2 ± 0.2

Table S6: Mean ± standard deviation (in degrees) root-mean-square errors between tibio-femoral joint rotations estimated by the IMU-based systems and the optical reference on harness, calculated across all trials of knee 1.

2.2 Averaged plots for individual knee 2

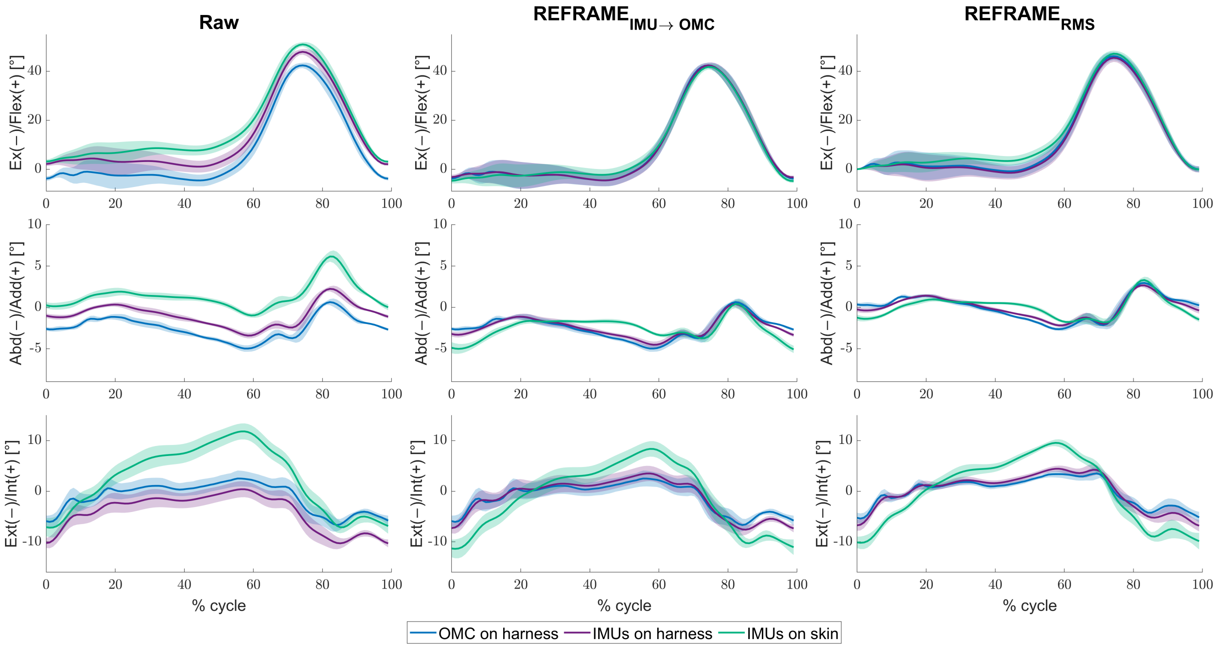

Figure S2: Mean tibio-femoral joint angles (solid lines) ± standard deviation (shaded areas), in degrees, as estimated by inertial measurement units (IMUs) on harness (purple), IMUs on skin (green), and optical motion capture (OMC) on harness (blue), averaged over all cycles for knee 2. Angles are shown as a percentage of the gait cycle under three conditions: 1) raw, i.e. in the absence of post-processing methods to correct reference frame orientation differences (left), 2) after implementation of REFRAME$_{IMU->OMC}$ (middle), and 3) after implementation of REFRAME$_{RMS}$ (right).

	Femur		Tibia	
	IMUs on harness	IMUs on skin	IMUs on harness	IMUs on skin
Rx	0.0 ± 0.0	0.0 ± 0.0	5.7 ± 0.1	8.9 ± 0.6
Ry	0.1 ± 0.3	-3.7 ± 1.0	-1.1 ± 0.3	-7.8 ± 1.1
Rz	0.5 ± 0.2	-0.3 ± 1.0	3.4 ± 0.2	-4.2 ± 1.1

Table S7: Mean ± standard deviation (in degrees) of the rotational transformations (Rx: Rotation around X, Ry: Rotation around Y, Rz: Rotation around Z) applied to the femoral and tibial segment frames as part of REFRAME$_{IMU->OMC}$, calculated across all cycles of knee 2.

	Femur			Tibia		
	OMC on harness	IMUs on harness	IMUs on skin	OMC on harness	IMUs on harness	IMUs on skin
Rx	0.0 ± 0.0	0.0 ± 0.0	0.0 ± 0.0	-3.8 ± 1.0	2.6 ± 0.9	3.7 ± 0.8
Ry	-3.2 ± 1.6	-3.5 ± 1.8	-3.4 ± 1.4	-0.6 ± 1.6	-2.4 ± 1.7	-4.5 ± 1.3
Rz	-0.1 ± 0.4	0.5 ± 0.4	-1.3 ± 0.8	0.7 ± 1.1	4.0 ± 1.3	-4.3 ± 2.0

Table S8: Mean ± standard deviation (in degrees) of the rotational transformations (Rx: Rotation around X, Ry: Rotation around Y, Rz: Rotation around Z) applied to the femoral and tibial segment frames as part of REFRAME$_{RMS}$, calculated across all cycles of knee 2.

	**Raw**		**REFRAME$_{IMU->OMC}$**		**REFRAME$_{RMS}$**	
	IMUs on harness	IMUs on skin	IMUs on harness	IMUs on skin	IMUs on harness	IMUs on skin
flexion/extension	5.6 ± 0.1	9.6 ± 0.5	0.5 ± 0.0	1.4 ± 0.2	0.8 ± 0.1	2.5 ± 0.7
abduction/adduction	1.6 ± 0.1	3.9 ± 0.3	0.4 ± 0.0	1.4 ± 0.1	0.4 ± 0.0	1.0 ± 0.1
external/internal rotation	3.0 ± 0.2	5.3 ± 0.3	0.9 ± 0.1	4.0 ± 0.2	0.9 ± 0.1	4.0 ± 0.2

Table S9: Mean ± standard deviation (in degrees) root-mean-square errors between tibio-femoral joint rotations estimated by the IMU-based systems and the optical reference on harness, calculated across all trials of knee 2.

2.3 Averaged plots for individual knee 3

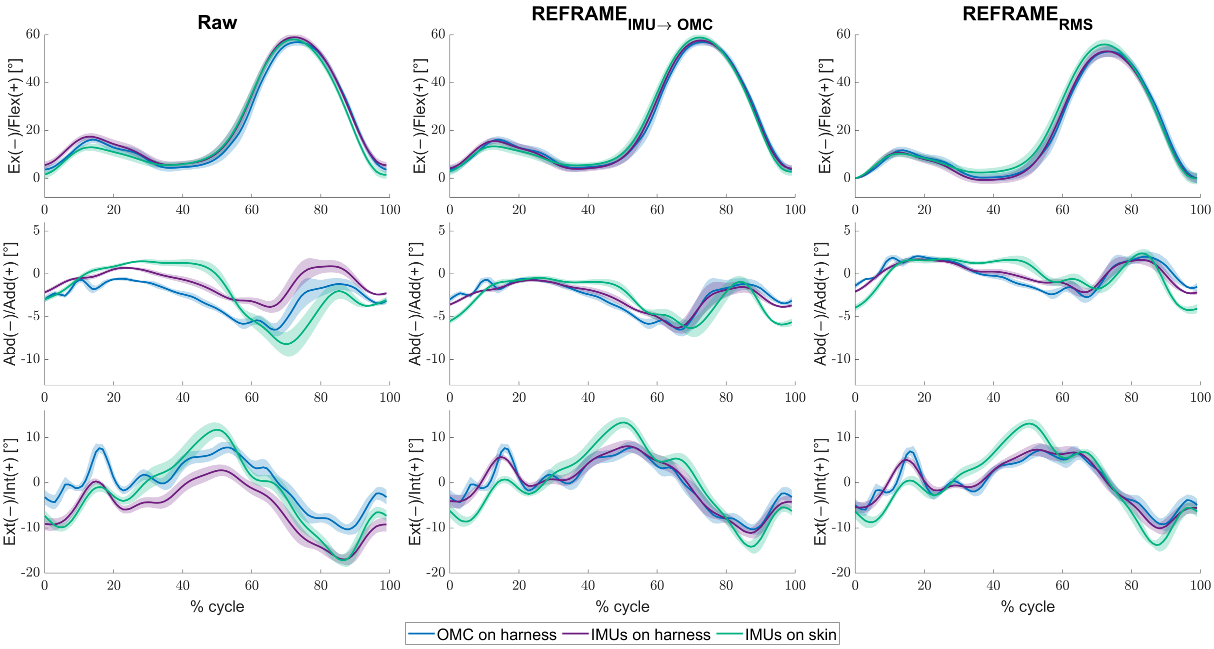

Figure S3: Mean tibio-femoral joint angles (solid lines) ± standard deviation (shaded areas), in degrees, as estimated by inertial measurement units (IMUs) on harness (purple), IMUs on skin (green), and optical motion capture (OMC) on harness (blue), averaged over all cycles for knee 3. Angles are shown as a percentage of the gait cycle under three conditions: 1) raw, i.e. in the absence of post-processing methods to correct reference frame orientation differences (left), 2) after implementation of $REFRAME_{IMU->OMC}$ (middle), and 3) after implementation of $REFRAME_{RMS}$ (right).

	Femur		Tibia	
	IMUs on harness	IMUs on skin	IMUs on harness	IMUs on skin
Rx	0.0 ± 0.0	0.0 ± 0.0	1.9 ± 0.1	-0.0 ± 0.2
Ry	-2.2 ± 0.2	-2.1 ± 0.9	-3.3 ± 0.2	-4.9 ± 0.9
Rz	-0.5 ± 0.3	6.7 ± 0.9	4.3 ± 0.2	7.8 ± 0.5

Table S10: Mean ± standard deviation (in degrees) of the rotational transformations (Rx: Rotation around X, Ry: Rotation around Y, Rz: Rotation around Z) applied to the femoral and tibial segment frames as part of $REFRAME_{IMU->OMC}$, calculated across all cycles of knee 3.

	Femur			Tibia		
	OMC on harness	IMUs on harness	IMUs on skin	OMC on harness	IMUs on harness	IMUs on skin
Rx	0.0 ± 0.0	0.0 ± 0.0	0.0 ± 0.0	3.9 ± 1.6	6.6 ± 1.5	2.7 ± 1.8
Ry	-5.6 ± 1.8	-6.8 ± 1.8	-2.7 ± 2.3	-3.8 ± 1.8	-5.7 ± 1.8	-3.3 ± 2.5
Rz	5.1 ± 1.1	4.4 ± 1.2	10.2 ± 1.8	3.2 ± 1.0	7.9 ± 1.0	11.1 ± 1.2

Table S11: Mean ± standard deviation (in degrees) of the rotational transformations (Rx: Rotation around X, Ry: Rotation around Y, Rz: Rotation around Z) applied to the femoral and tibial segment frames as part of $REFRAME_{RMS}$, calculated across all cycles of knee 3.

	**Raw**		**$REFRAME_{IMU->OMC}$**		**$REFRAME_{RMS}$**	
	IMUs on harness	IMUs on skin	IMUs on harness	IMUs on skin	IMUs on harness	IMUs on skin
flexion/extension	2.2 ± 0.1	2.8 ± 0.3	1.3 ± 0.2	2.4 ± 0.4	1.4 ± 0.2	3.0 ± 0.5
abduction/adduction	1.8 ± 0.1	2.4 ± 0.3	0.6 ± 0.1	1.6 ± 0.2	0.7 ± 0.1	1.6 ± 0.2
external/internal rotation	5.7 ± 0.2	4.5 ± 0.4	1.3 ± 0.1	3.9 ± 0.4	1.3 ± 0.1	3.9 ± 0.4

Table S12: Mean ± standard deviation (in degrees) root-mean-square errors between tibio-femoral joint rotations estimated by the IMU-based systems and the optical reference on harness, calculated across all trials of knee 3.

2.4 Averaged plots for individual knee 4

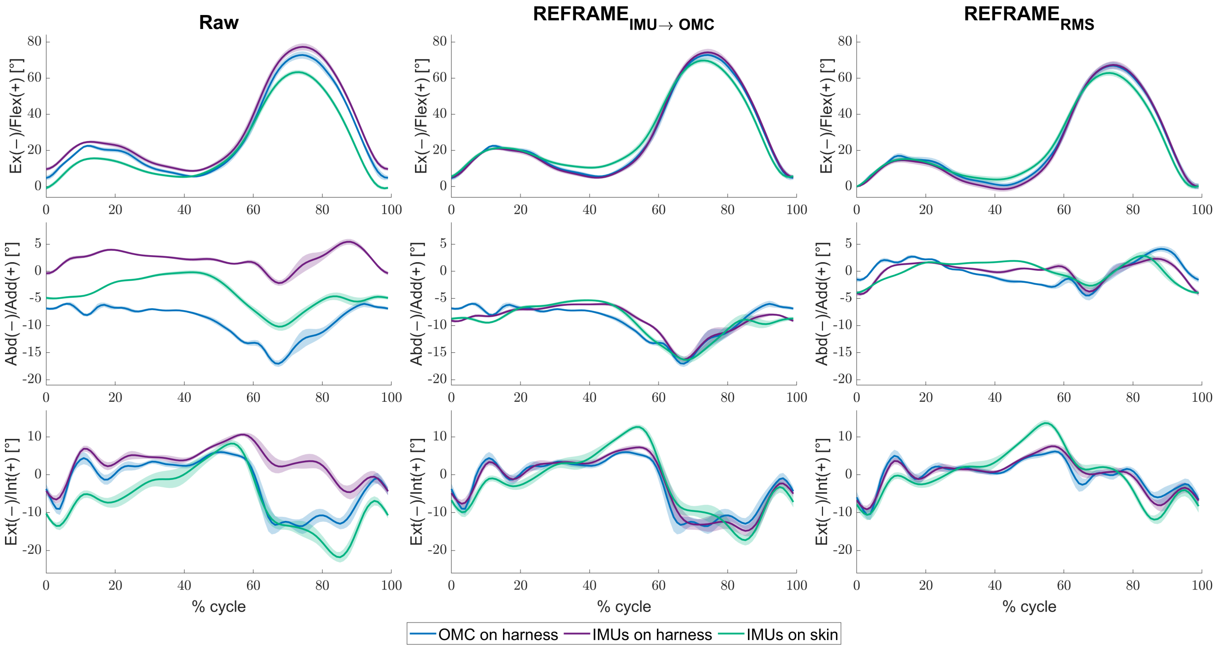

Figure S4: Mean tibio-femoral joint angles (solid lines) ± standard deviation (shaded areas), in degrees, as estimated by inertial measurement units (IMUs) on harness (purple), IMUs on skin (green), and optical motion capture (OMC) on harness (blue), averaged over all cycles for knee 4. Angles are shown as a percentage of the gait cycle under three conditions: 1) raw, i.e. in the absence of post-processing methods to correct reference frame orientation differences (left), 2) after implementation of REFRAME$_{IMU->OMC}$ (middle), and 3) after implementation of REFRAME$_{RMS}$ (right).

	Femur		Tibia	
	IMUs on harness	IMUs on skin	IMUs on harness	IMUs on skin
Rx	0.0 ± 0.0	0.0 ± 0.0	2.6 ± 0.1	-5.4 ± 0.2
Ry	7.7 ± 0.2	-2.4 ± 0.8	0.4 ± 0.2	-7.1 ± 1.0
Rz	-12.6 ± 0.2	-1.6 ± 1.0	-12.4 ± 0.2	2.2 ± 0.6

Table S13: Mean ± standard deviation (in degrees) of the rotational transformations (Rx: Rotation around X, Ry: Rotation around Y, Rz: Rotation around Z) applied to the femoral and tibial segment frames as part of REFRAME$_{IMU->OMC}$, calculated across all cycles of knee 4.

	Femur			Tibia		
	OMC on harness	IMUs on harness	IMUs on skin	OMC on harness	IMUs on harness	IMUs on skin
Rx	0.0 ± 0.0	0.0 ± 0.0	0.0 ± 0.0	5.2 ± 1.2	9.9 ± 1.1	1.1 ± 1.1
Ry	-7.4 ± 1.7	0.4 ± 1.5	-7.6 ± 1.9	-1.7 ± 1.7	-2.6 ± 1.6	-6.5 ± 1.9
Rz	13.5 ± 0.8	1.0 ± 0.9	12.0 ± 1.2	10.8 ± 0.8	-1.6 ± 0.9	14.6 ± 0.8

Table S14: Mean ± standard deviation (in degrees) of the rotational transformations (Rx: Rotation around X, Ry: Rotation around Y, Rz: Rotation around Z) applied to the femoral and tibial segment frames as part of REFRAME$_{RMS}$, calculated across all cycles of knee 4.

	**Raw**		**REFRAME$_{IMU->OMC}$**		**REFRAME$_{RMS}$**	
	IMUs on harness	IMUs on skin	IMUs on harness	IMUs on skin	IMUs on harness	IMUs on skin
flexion/extension	3.9 ± 0.1	6.9 ± 0.1	1.3 ± 0.0	3.6 ± 0.2	1.7 ± 0.2	3.5 ± 0.2
abduction/adduction	11.5 ± 0.1	5.4 ± 0.2	1.6 ± 0.1	1.8 ± 0.1	1.7 ± 0.1	2.5 ± 0.1
external/internal rotation	7.7 ± 0.2	5.6 ± 0.3	1.4 ± 0.2	3.7 ± 0.4	1.3 ± 0.1	4.0 ± 0.4

Table S15: Mean ± standard deviation (in degrees) root-mean-square errors between tibio-femoral joint rotations estimated by the IMU-based systems and the optical reference on harness, calculated across all trials of knee 4.

2.5 Averaged plots for individual knee 5

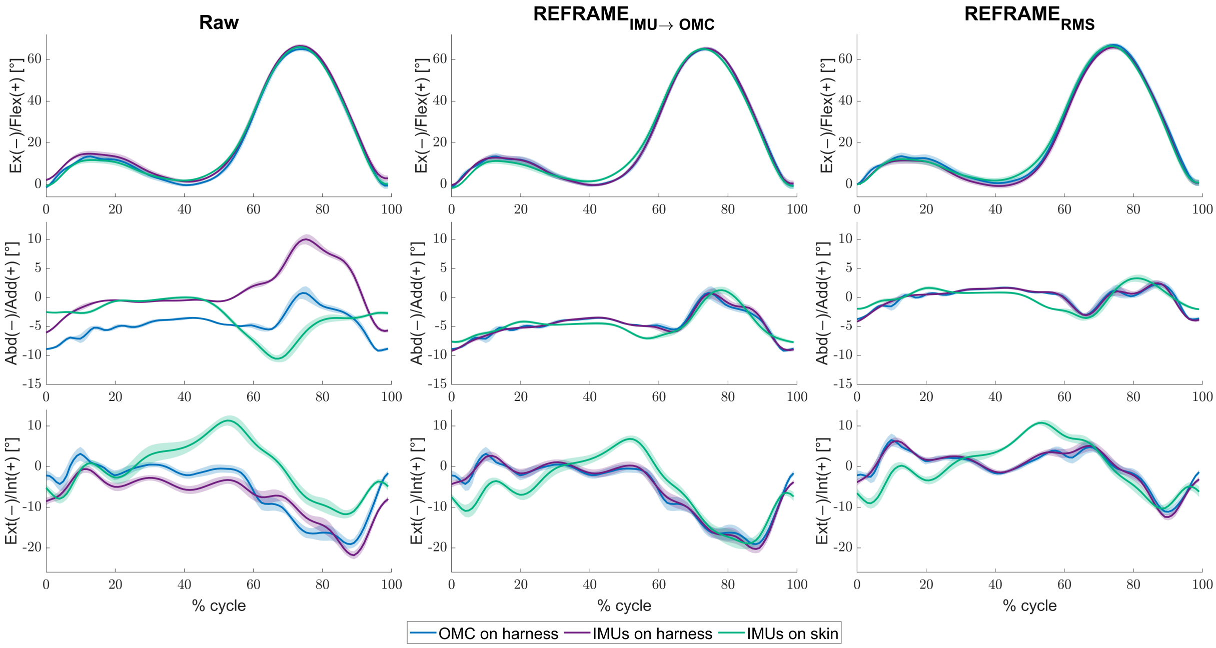

Figure S5: Mean tibio-femoral joint angles (solid lines) ± standard deviation (shaded areas), in degrees, as estimated by inertial measurement units (IMUs) on harness (purple), IMUs on skin (green), and optical motion capture (OMC) on harness (blue), averaged over all cycles for knee 5. Angles are shown as a percentage of the gait cycle under three conditions: 1) raw, i.e. in the absence of post-processing methods to correct reference frame orientation differences (left), 2) after implementation of REFRAME$_{IMU->OMC}$ (middle), and 3) after implementation of REFRAME$_{RMS}$ (right).

	Femur		Tibia	
	IMUs on harness	IMUs on skin	IMUs on harness	IMUs on skin
Rx	0.0 ± 0.0	0.0 ± 0.0	1.2 ± 0.1	1.3 ± 0.3
Ry	2.7 ± 0.3	9.9 ± 1.2	-0.3 ± 0.3	5.2 ± 1.2
Rz	-8.5 ± 0.2	6.7 ± 0.9	-4.4 ± 0.1	3.9 ± 0.4

Table S16: Mean ± standard deviation (in degrees) of the rotational transformations (Rx: Rotation around X, Ry: Rotation around Y, Rz: Rotation around Z) applied to the femoral and tibial segment frames as part of REFRAME$_{IMU->OMC}$, calculated across all cycles of knee 5.

	Femur			Tibia		
	OMC on harness	IMUs on harness	IMUs on skin	OMC on harness	IMUs on harness	IMUs on skin
Rx	0.0 ± 0.0	0.0 ± 0.0	0.0 ± 0.0	-0.7 ± 0.5	3.2 ± 0.6	-0.3 ± 0.8
Ry	-14.3 ± 0.9	-11.8 ± 0.9	-2.2 ± 1.5	-9.2 ± 0.9	-9.5 ± 1.0	-1.7 ± 1.4
Rz	3.8 ± 0.5	-4.3 ± 0.6	8.9 ± 1.0	4.1 ± 0.6	0.2 ± 0.5	7.6 ± 0.7

Table S17: Mean ± standard deviation (in degrees) of the rotational transformations (Rx: Rotation around X, Ry: Rotation around Y, Rz: Rotation around Z) applied to the femoral and tibial segment frames as part of REFRAME$_{RMS}$, calculated across all cycles of knee 5.

	**Raw**		**REFRAME$_{IMU->OMC}$**		**REFRAME$_{RMS}$**	
	IMUs on harness	IMUs on skin	IMUs on harness	IMUs on skin	IMUs on harness	IMUs on skin
flexion/extension	1.9 ± 0.1	1.7 ± 0.2	0.9 ± 0.2	2.1 ± 0.3	1.7 ± 0.2	1.9 ± 0.4
abduction/adduction	6.0 ± 0.1	4.3 ± 0.2	0.4 ± 0.1	1.2 ± 0.1	0.4 ± 0.1	1.4 ± 0.1
external/internal rotation	3.4 ± 0.2	7.4 ± 0.3	0.9 ± 0.2	4.6 ± 0.2	0.8 ± 0.1	4.8 ± 0.2

Table S18: Mean ± standard deviation (in degrees) root-mean-square errors between tibio-femoral joint rotations estimated by the IMU-based systems and the optical reference on harness, calculated across all trials of knee 5.

2.6 Averaged plots for individual knee 6

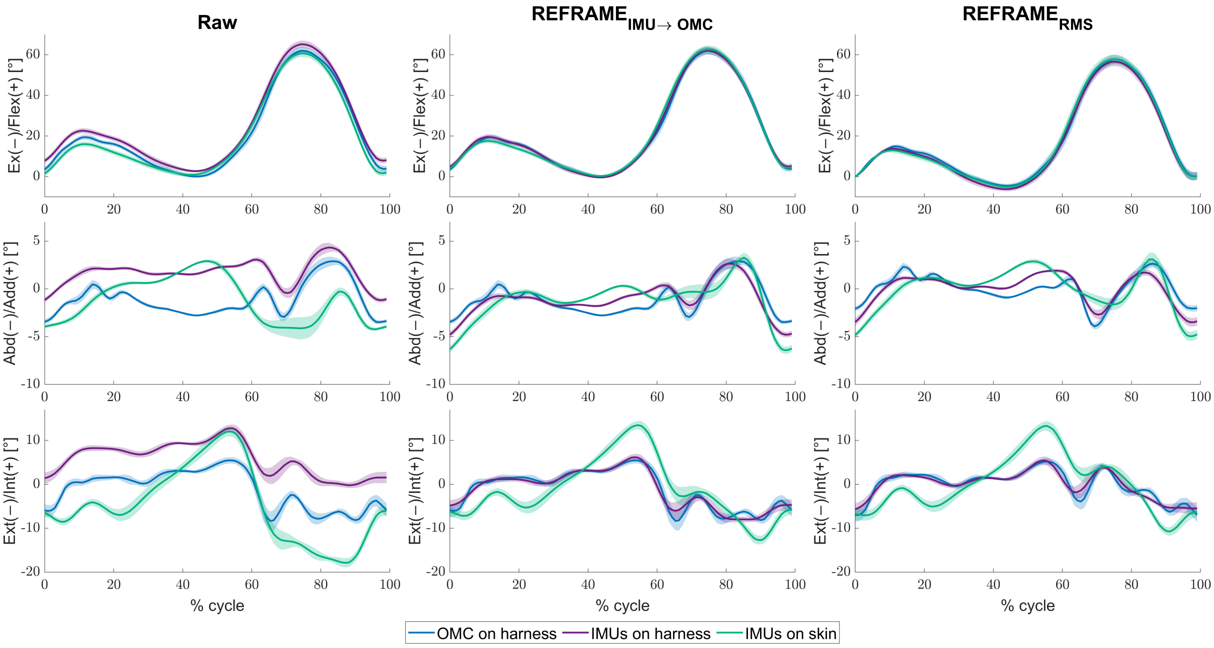

Figure S6: Mean tibio-femoral joint angles (solid lines) ± standard deviation (shaded areas), in degrees, as estimated by inertial measurement units (IMUs) on harness (purple), IMUs on skin (green), and optical motion capture (OMC) on harness (blue), averaged over all cycles for knee 6. Angles are shown as a percentage of the gait cycle under three conditions: 1) raw, i.e. in the absence of post-processing methods to correct reference frame orientation differences (left), 2) after implementation of REFRAME$_{IMU->OMC}$ (middle), and 3) after implementation of REFRAME$_{RMS}$ (right).

	Femur		Tibia	
	IMUs on harness	IMUs on skin	IMUs on harness	IMUs on skin
Rx	0.0 ± 0.0	0.0 ± 0.0	3.0 ± 0.1	-0.3 ± 0.3
Ry	3.1 ± 0.2	-6.5 ± 1.0	-0.7 ± 0.2	-9.6 ± 1.1
Rz	1.0 ± 0.1	11.4 ± 0.4	-5.0 ± 0.1	11.5 ± 0.3

Table S19: Mean ± standard deviation (in degrees) of the rotational transformations (Rx: Rotation around X, Ry: Rotation around Y, Rz: Rotation around Z) applied to the femoral and tibial segment frames as part of REFRAME$_{IMU->OMC}$, calculated across all cycles of knee 6.

	Femur			Tibia		
	OMC on harness	IMUs on harness	IMUs on skin	OMC on harness	IMUs on harness	IMUs on skin
Rx	0.0 ± 0.0	0.0 ± 0.0	0.0 ± 0.0	4.3 ± 1.1	7.7 ± 1.0	4.2 ± 1.2
Ry	-8.7 ± 0.6	-5.8 ± 0.7	-14.1 ± 1.8	-7.0 ± 0.6	-8.3 ± 0.7	-14.6 ± 1.8
Rz	1.1 ± 0.5	2.4 ± 0.4	12.1 ± 0.8	-0.2 ± 0.5	-4.8 ± 0.4	12.1 ± 0.5

Table S20: Mean ± standard deviation (in degrees) of the rotational transformations (Rx: Rotation around X, Ry: Rotation around Y, Rz: Rotation around Z) applied to the femoral and tibial segment frames as part of REFRAME$_{RMS}$, calculated across all cycles of knee 6.

	**Raw**		**REFRAME$_{IMU->OMC}$**		**REFRAME$_{RMS}$**	
	IMUs on harness	IMUs on skin	IMUs on harness	IMUs on skin	IMUs on harness	IMUs on skin
flexion/extension	3.3 ± 0.1	2.7 ± 0.4	1.1 ± 0.2	1.7 ± 0.3	1.7 ± 0.5	1.6 ± 0.3
abduction/adduction	3.0 ± 0.1	3.1 ± 0.1	1.0 ± 0.0	1.7 ± 0.1	1.0 ± 0.0	1.8 ± 0.1
external/internal rotation	7.1 ± 0.1	6.4 ± 0.3	0.9 ± 0.1	4.5 ± 0.3	1.0 ± 0.1	4.5 ± 0.3

Table S21: Mean ± standard deviation (in degrees) root-mean-square errors between tibio-femoral joint rotations estimated by the IMU-based systems and the optical reference on harness, calculated across all trials of knee 6.

2.7 Averaged plots for individual knee 7

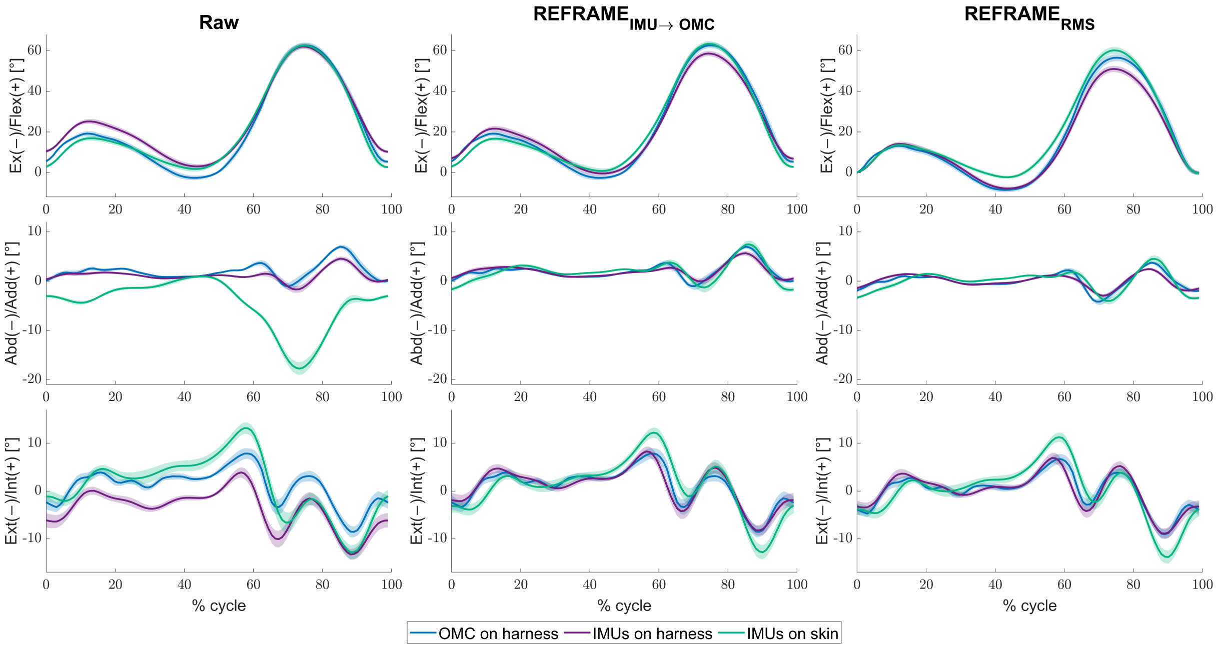

Figure S7: Mean tibio-femoral joint angles (solid lines) ± standard deviation (shaded areas), in degrees, as estimated by inertial measurement units (IMUs) on harness (purple), IMUs on skin (green), and optical motion capture (OMC) on harness (blue), averaged over all cycles for knee 7. Angles are shown as a percentage of the gait cycle under three conditions: 1) raw, i.e. in the absence of post-processing methods to correct reference frame orientation differences (left), 2) after implementation of REFRAME$_{IMU->OMC}$ (middle), and 3) after implementation of REFRAME$_{RMS}$ (right).

	Femur		Tibia	
	IMUs on harness	IMUs on skin	IMUs on harness	IMUs on skin
Rx	0.0 ± 0.0	0.0 ± 0.0	3.5 ± 0.2	0.5 ± 0.1
Ry	-1.3 ± 0.5	0.7 ± 1.0	-1.0 ± 0.5	1.3 ± 1.0
Rz	2.6 ± 0.2	17.6 ± 0.5	6.7 ± 0.3	15.6 ± 0.4

Table S22: Mean ± standard deviation (in degrees) of the rotational transformations (Rx: Rotation around X, Ry: Rotation around Y, Rz: Rotation around Z) applied to the femoral and tibial segment frames as part of $\text{REFRAME}_{IMU->OMC}$, calculated across all cycles of knee 7.

	Femur			Tibia		
	OMC on harness	IMUs on harness	IMUs on skin	OMC on harness	IMUs on harness	IMUs on skin
Rx	0.0 ± 0.0	0.0 ± 0.0	0.0 ± 0.0	5.8 ± 0.7	11.0 ± 0.9	3.9 ± 0.9
Ry	-3.0 ± 0.7	-3.9 ± 0.9	0.5 ± 1.5	-4.7 ± 0.7	-4.5 ± 0.9	0.3 ± 1.4
Rz	-0.2 ± 0.4	2.2 ± 0.4	16.1 ± 0.8	-1.7 ± 0.5	5.1 ± 0.5	13.2 ± 0.7

Table S23: Mean ± standard deviation (in degrees) of the rotational transformations (Rx: Rotation around X, Ry: Rotation around Y, Rz: Rotation around Z) applied to the femoral and tibial segment frames as part of REFRAME_{RMS}, calculated across all cycles of knee 7.

	**Raw**		**$\text{REFRAME}_{IMU->OMC}$**		**REFRAME_{RMS}**	
	IMUs on harness	IMUs on skin	IMUs on harness	IMUs on skin	IMUs on harness	IMUs on skin
flexion/extension	4.5 ± 0.1	3.0 ± 0.1	2.8 ± 0.1	2.9 ± 0.2	3.2 ± 0.2	4.1 ± 0.3
abduction/adduction	1.2 ± 0.1	8.6 ± 0.3	0.7 ± 0.1	1.0 ± 0.1	0.7 ± 0.1	1.1 ± 0.1
external/internal rotation	5.0 ± 0.2	3.5 ± 0.2	1.1 ± 0.2	2.6 ± 0.2	1.1 ± 0.2	2.7 ± 0.2

Table S24: Mean ± standard deviation (in degrees) root-mean-square errors between tibio-femoral joint rotations estimated by the IMU-based systems and the optical reference on harness, calculated across all trials of knee 7.

2.8 Averaged plots for individual knee 8

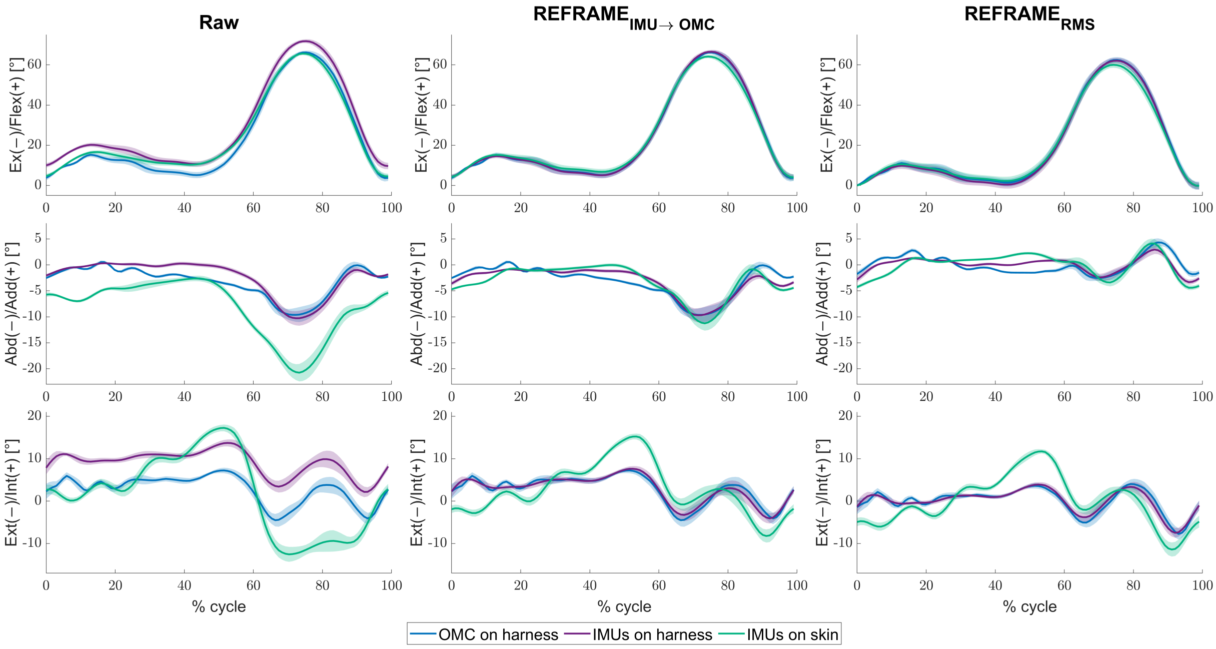

Figure S8: Mean tibio-femoral joint angles (solid lines) ± standard deviation (shaded areas), in degrees, as estimated by inertial measurement units (IMUs) on harness (purple), IMUs on skin (green), and optical motion capture (OMC) on harness (blue), averaged over all cycles for knee 8. Angles are shown as a percentage of the gait cycle under three conditions: 1) raw, i.e. in the absence of post-processing methods to correct reference frame orientation differences (left), 2) after implementation of REFRAME$_{IMU->OMC}$ (middle), and 3) after implementation of REFRAME$_{RMS}$ (right).

	Femur		Tibia	
	IMUs on harness	IMUs on skin	IMUs on harness	IMUs on skin
Rx	0.0 ± 0.0	0.0 ± 0.0	5.8 ± 0.1	1.3 ± 0.2
Ry	2.8 ± 0.2	-10.6 ± 0.9	0.4 ± 0.2	-10.7 ± 1.0
Rz	0.9 ± 0.3	17.5 ± 0.6	-4.7 ± 0.2	12.1 ± 0.5

Table S25: Mean ± standard deviation (in degrees) of the rotational transformations (Rx: Rotation around X, Ry: Rotation around Y, Rz: Rotation around Z) applied to the femoral and tibial segment frames as part of REFRAME$_{IMU->OMC}$, calculated across all cycles of knee 8.

	Femur			Tibia		
	OMC on harness	IMUs on harness	IMUs on skin	OMC on harness	IMUs on harness	IMUs on skin
Rx	0.0 ± 0.0	0.0 ± 0.0	0.0 ± 0.0	4.0 ± 1.2	10.6 ± 1.1	6.0 ± 1.0
Ry	-0.0 ± 1.0	2.9 ± 1.1	-9.2 ± 2.1	0.6 ± 0.9	0.7 ± 1.0	-7.6 ± 1.8
Rz	7.8 ± 0.9	8.7 ± 0.9	24.9 ± 1.3	3.9 ± 1.0	-0.7 ± 1.1	17.0 ± 1.3

Table S26: Mean ± standard deviation (in degrees) of the rotational transformations (Rx: Rotation around X, Ry: Rotation around Y, Rz: Rotation around Z) applied to the femoral and tibial segment frames as part of REFRAME$_{RMS}$, calculated across all cycles of knee 8.

	**Raw**		**REFRAME$_{IMU->OMC}$**		**REFRAME$_{RMS}$**	
	IMUs on harness	IMUs on skin	IMUs on harness	IMUs on skin	IMUs on harness	IMUs on skin
flexion/extension	5.6 ± 0.1	3.0 ± 0.2	0.9 ± 0.1	1.6 ± 0.2	1.2 ± 0.2	1.7 ± 0.3
abduction/adduction	1.5 ± 0.1	6.1 ± 0.2	1.2 ± 0.1	1.8 ± 0.1	1.3 ± 0.1	2.2 ± 0.1
external/internal rotation	6.3 ± 0.3	7.2 ± 0.3	0.8 ± 0.1	4.6 ± 0.2	0.8 ± 0.1	4.5 ± 0.2

Table S27: Mean ± standard deviation (in degrees) root-mean-square errors between tibio-femoral joint rotations estimated by the IMU-based systems and the optical reference on harness, calculated across all trials of knee 8.

2.9 Averaged plots for individual knee 9

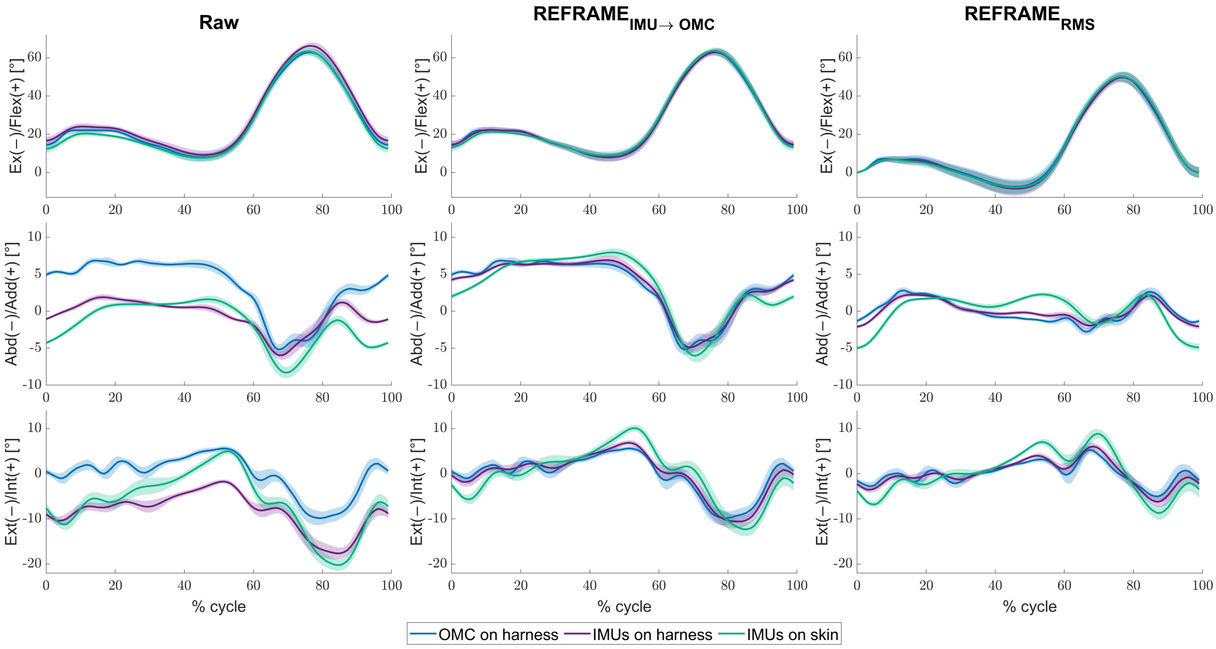

Figure S9: Mean tibio-femoral joint angles (solid lines) ± standard deviation (shaded areas), in degrees, as estimated by inertial measurement units (IMUs) on harness (purple), IMUs on skin (green), and optical motion capture (OMC) on harness (blue), averaged over all cycles for knee 9. Angles are shown as a percentage of the gait cycle under three conditions: 1) raw, i.e. in the absence of post-processing methods to correct reference frame orientation differences (left), 2) after implementation of REFRAME$_{IMU->OMC}$ (middle), and 3) after implementation of REFRAME$_{RMS}$ (right).

	Femur		Tibia	
	IMUs on harness	IMUs on skin	IMUs on harness	IMUs on skin
Rx	0.0 ± 0.0	0.0 ± 0.0	1.8 ± 0.1	-0.8 ± 0.2
Ry	-1.7 ± 0.2	-6.3 ± 0.7	5.7 ± 0.2	0.5 ± 0.7
Rz	-6.6 ± 0.2	-1.9 ± 0.6	1.7 ± 0.2	1.9 ± 0.6

Table S28: Mean ± standard deviation (in degrees) of the rotational transformations (Rx: Rotation around X, Ry: Rotation around Y, Rz: Rotation around Z) applied to the femoral and tibial segment frames as part of $\text{REFRAME}_{IMU->OMC}$, calculated across all cycles of knee 9.

	Femur			Tibia		
	OMC on harness	IMUs on harness	IMUs on skin	OMC on harness	IMUs on harness	IMUs on skin
Rx	0.0 ± 0.0	0.0 ± 0.0	0.0 ± 0.0	14.8 ± 2.1	17.8 ± 2.0	14.7 ± 2.1
Ry	-4.2 ± 1.7	-6.3 ± 1.6	-10.3 ± 1.6	-10.3 ± 1.7	-4.6 ± 1.5	-9.1 ± 1.5
Rz	16.1 ± 0.8	9.6 ± 0.8	14.3 ± 1.1	15.3 ± 0.7	15.6 ± 0.7	17.2 ± 0.9

Table S29: Mean ± standard deviation (in degrees) of the rotational transformations (Rx: Rotation around X, Ry: Rotation around Y, Rz: Rotation around Z) applied to the femoral and tibial segment frames as part of REFRAME_{RMS}, calculated across all cycles of knee 9.

	**Raw**		$\textbf{REFRAME}_{IMU->OMC}$		$\textbf{REFRAME}_{RMS}$	
	IMUs on harness	IMUs on skin	IMUs on harness	IMUs on skin	IMUs on harness	IMUs on skin
flexion/extension	2.3 ± 0.1	1.6 ± 0.2	0.7 ± 0.1	1.3 ± 0.1	1.1 ± 0.1	1.1 ± 0.2
abduction/adduction	4.5 ± 0.1	5.6 ± 0.1	0.5 ± 0.0	1.6 ± 0.1	0.6 ± 0.0	2.2 ± 0.1
external/internal rotation	8.3 ± 0.1	7.0 ± 0.3	1.1 ± 0.1	3.2 ± 0.1	1.0 ± 0.2	2.9 ± 0.1

Table S30: Mean ± standard deviation (in degrees) root-mean-square errors between tibio-femoral joint rotations estimated by the IMU-based systems and the optical reference on harness, calculated across all trials of knee 9.

2.10 Averaged plots for individual knee 10

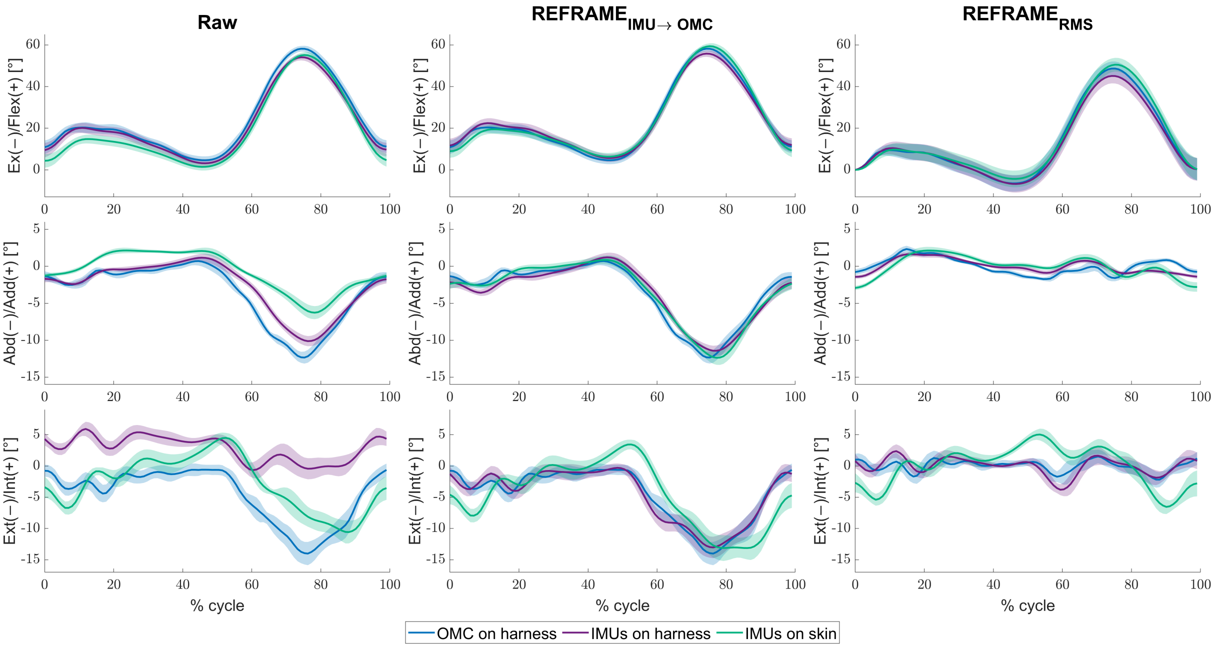

Figure S10: Mean tibio-femoral joint angles (solid lines) ± standard deviation (shaded areas), in degrees, as estimated by inertial measurement units (IMUs) on harness (purple), IMUs on skin (green), and optical motion capture (OMC) on harness (blue), averaged over all cycles for knee 10. Angles are shown as a percentage of the gait cycle under three conditions: 1) raw, i.e. in the absence of post-processing methods to correct reference frame orientation differences (left), 2) after implementation of REFRAME$_{IMU->OMC}$ (middle), and 3) after implementation of REFRAME$_{RMS}$ (right).

	Femur		Tibia	
	IMUs on harness	IMUs on skin	IMUs on harness	IMUs on skin
Rx	0.0 ± 0.0	0.0 ± 0.0	-1.9 ± 0.1	-4.8 ± 0.2
Ry	6.8 ± 0.4	-0.7 ± 1.2	7.6 ± 0.3	-0.9 ± 1.2
Rz	-6.1 ± 0.4	-7.4 ± 0.7	-10.1 ± 0.3	-8.5 ± 0.3

Table S31: Mean ± standard deviation (in degrees) of the rotational transformations (Rx: Rotation around X, Ry: Rotation around Y, Rz: Rotation around Z) applied to the femoral and tibial segment frames as part of REFRAME$_{IMU->OMC}$, calculated across all cycles of knee 10.

	Femur			Tibia		
	OMC on harness	IMUs on harness	IMUs on skin	OMC on harness	IMUs on harness	IMUs on skin
Rx	0.0 ± 0.0	0.0 ± 0.0	0.0 ± 0.0	11.1 ± 3.2	9.9 ± 3.4	5.4 ± 3.4
Ry	-5.5 ± 1.5	1.2 ± 1.6	-5.7 ± 1.7	-4.7 ± 1.6	0.7 ± 1.5	-6.9 ± 1.7
Rz	19.0 ± 1.0	13.4 ± 1.1	11.5 ± 1.2	20.5 ± 1.0	9.6 ± 0.9	12.2 ± 1.2

Table S32: Mean ± standard deviation (in degrees) of the rotational transformations (Rx: Rotation around X, Ry: Rotation around Y, Rz: Rotation around Z) applied to the femoral and tibial segment frames as part of REFRAME$_{RMS}$, calculated across all cycles of knee 10.

	**Raw**		**REFRAME$_{IMU->OMC}$**		**REFRAME$_{RMS}$**	
	IMUs on harness	IMUs on skin	IMUs on harness	IMUs on skin	IMUs on harness	IMUs on skin
flexion/extension	2.5 ± 0.1	4.9 ± 0.2	1.6 ± 0.1	1.7 ± 0.2	2.0 ± 0.2	1.9 ± 0.3
abduction/adduction	1.4 ± 0.1	3.5 ± 0.2	1.0 ± 0.1	0.9 ± 0.1	0.8 ± 0.0	1.3 ± 0.2
external/internal rotation	8.0 ± 0.3	3.9 ± 0.3	1.0 ± 0.1	3.2 ± 0.3	1.1 ± 0.1	3.1 ± 0.3

Table S33: Mean ± standard deviation (in degrees) root-mean-square errors between tibio-femoral joint rotations estimated by the IMU-based systems and the optical reference on harness, calculated across all trials of knee 10.

2.11 Averaged plots for individual knee 11

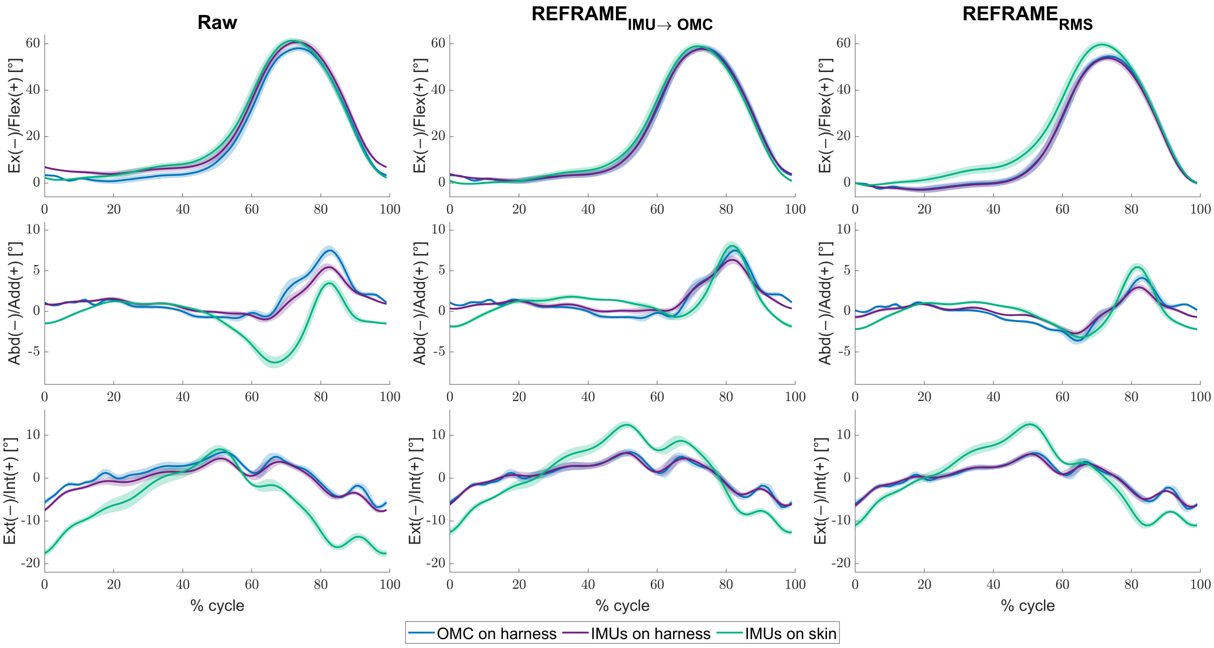

Figure S11: Mean tibio-femoral joint angles (solid lines) ± standard deviation (shaded areas), in degrees, as estimated by inertial measurement units (IMUs) on harness (purple), IMUs on skin (green), and optical motion capture (OMC) on harness (blue), averaged over all cycles for knee 11. Angles are shown as a percentage of the gait cycle under three conditions: 1) raw, i.e. in the absence of post-processing methods to correct reference frame orientation differences (left), 2) after implementation of REFRAME$_{IMU->OMC}$ (middle), and 3) after implementation of REFRAME$_{RMS}$ (right).

	Femur		Tibia	
	IMUs on harness	IMUs on skin	IMUs on harness	IMUs on skin
Rx	0.0 ± 0.0	0.0 ± 0.0	2.9 ± 0.1	2.5 ± 0.1
Ry	1.5 ± 0.2	-2.4 ± 0.5	1.2 ± 0.2	-2.2 ± 0.5
Rz	1.2 ± 0.1	8.1 ± 0.6	2.7 ± 0.1	12.9 ± 0.2

Table S34: Mean ± standard deviation (in degrees) of the rotational transformations (Rx: Rotation around X, Ry: Rotation around Y, Rz: Rotation around Z) applied to the femoral and tibial segment frames as part of REFRAME$_{IMU->OMC}$, calculated across all cycles of knee 11.

	Femur			Tibia		
	OMC on harness	IMUs on harness	IMUs on skin	OMC on harness	IMUs on harness	IMUs on skin
Rx	0.0 ± 0.0	0.0 ± 0.0	0.0 ± 0.0	3.5 ± 0.5	6.9 ± 0.4	1.6 ± 0.8
Ry	-0.6 ± 0.8	1.1 ± 0.7	3.0 ± 1.1	-1.2 ± 0.9	0.4 ± 0.8	3.0 ± 1.1
Rz	-3.6 ± 0.3	-2.4 ± 0.5	2.4 ± 0.9	-4.0 ± 0.3	-1.2 ± 0.3	8.9 ± 0.3

Table S35: Mean ± standard deviation (in degrees) of the rotational transformations (Rx: Rotation around X, Ry: Rotation around Y, Rz: Rotation around Z) applied to the femoral and tibial segment frames as part of REFRAME$_{RMS}$, calculated across all cycles of knee 11.

	**Raw**		**REFRAME$_{IMU->OMC}$**		**REFRAME$_{RMS}$**	
	IMUs on harness	IMUs on skin	IMUs on harness	IMUs on skin	IMUs on harness	IMUs on skin
flexion/extension	3.0 ± 0.1	3.6 ± 0.1	0.7 ± 0.1	2.3 ± 0.1	0.9 ± 0.1	5.1 ± 0.4
abduction/adduction	0.9 ± 0.1	3.4 ± 0.2	0.6 ± 0.0	1.6 ± 0.1	0.6 ± 0.0	1.3 ± 0.1
external/internal rotation	1.3 ± 0.1	7.8 ± 0.3	0.5 ± 0.1	4.4 ± 0.2	0.6 ± 0.1	4.1 ± 0.2

Table S36: Mean ± standard deviation (in degrees) root-mean-square errors between tibio-femoral joint rotations estimated by the IMU-based systems and the optical reference on harness, calculated across all trials of knee 11.

2.12 Averaged plots for individual knee 12

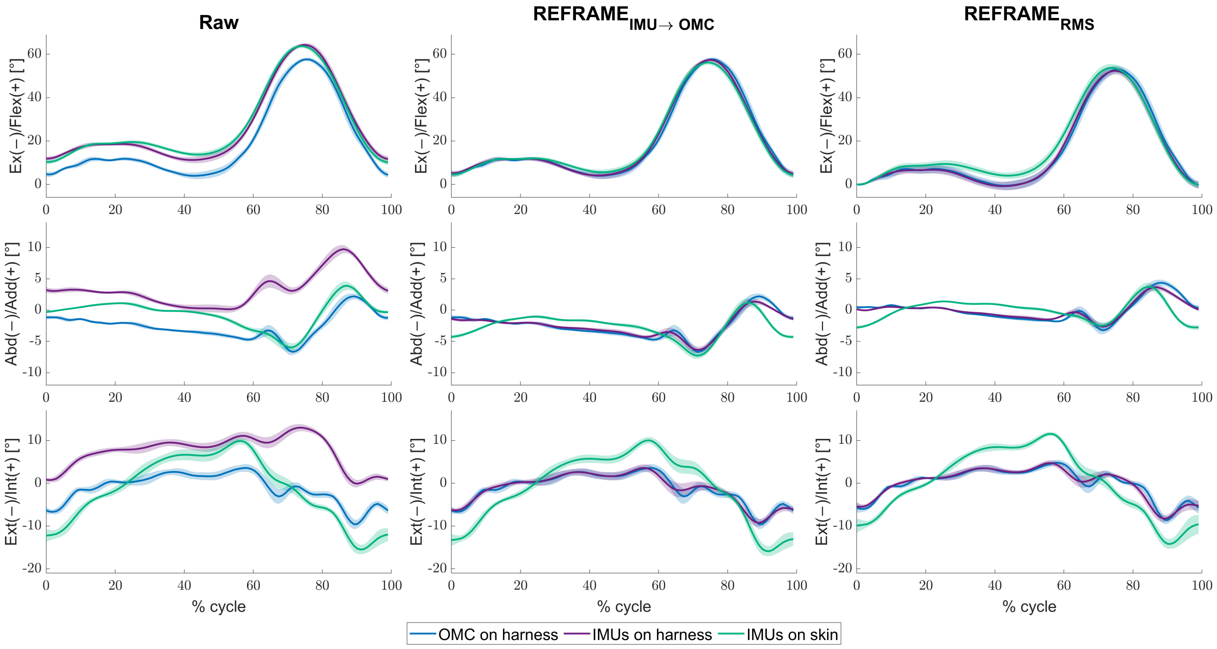

Figure S12: Mean tibio-femoral joint angles (solid lines) ± standard deviation (shaded areas), in degrees, as estimated by inertial measurement units (IMUs) on harness (purple), IMUs on skin (green), and optical motion capture (OMC) on harness (blue), averaged over all cycles for knee 12. Angles are shown as a percentage of the gait cycle under three conditions: 1) raw, i.e. in the absence of post-processing methods to correct reference frame orientation differences (left), 2) after implementation of $\text{REFRAME}_{IMU->OMC}$ (middle), and 3) after implementation of REFRAME_{RMS} (right).

	Femur		Tibia	
	IMUs on harness	IMUs on skin	IMUs on harness	IMUs on skin
Rx	0.0 ± 0.0	0.0 ± 0.0	6.5 ± 0.1	7.5 ± 0.2
Ry	2.6 ± 0.2	-3.8 ± 1.1	-1.1 ± 0.2	-6.7 ± 1.4
Rz	-10.0 ± 0.1	3.9 ± 1.0	-16.5 ± 0.1	2.6 ± 0.4

Table S37: Mean ± standard deviation (in degrees) of the rotational transformations (Rx: Rotation around X, Ry: Rotation around Y, Rz: Rotation around Z) applied to the femoral and tibial segment frames as part of $\text{REFRAME}_{IMU->OMC}$, calculated across all cycles of knee 12.

	Femur			Tibia		
	OMC on harness	IMUs on harness	IMUs on skin	OMC on harness	IMUs on harness	IMUs on skin
Rx	0.0 ± 0.0	0.0 ± 0.0	0.0 ± 0.0	4.6 ± 1.3	11.9 ± 1.1	10.0 ± 1.2
Ry	-1.3 ± 1.3	1.9 ± 1.3	3.3 ± 1.9	0.9 ± 1.2	-1.3 ± 1.3	3.0 ± 2.1
Rz	2.3 ± 0.5	-7.6 ± 0.6	3.2 ± 1.3	2.9 ± 0.6	-13.1 ± 0.6	5.3 ± 0.5

Table S38: Mean ± standard deviation (in degrees) of the rotational transformations (Rx: Rotation around X, Ry: Rotation around Y, Rz: Rotation around Z) applied to the femoral and tibial segment frames as part of REFRAME_{RMS}, calculated across all cycles of knee 12.

	**Raw**		**REFRAME$_{IMU->OMC}$**		**REFRAME$_{RMS}$**	
	IMUs on harness	IMUs on skin	IMUs on harness	IMUs on skin	IMUs on harness	IMUs on skin
flexion/extension	7.1 ± 0.1	8.0 ± 0.2	1.3 ± 0.2	2.1 ± 0.2	1.4 ± 0.2	3.8 ± 0.4
abduction/adduction	6.2 ± 0.1	2.3 ± 0.1	0.4 ± 0.0	1.7 ± 0.0	0.4 ± 0.0	1.9 ± 0.1
external/internal rotation	9.0 ± 0.1	4.5 ± 0.2	0.7 ± 0.1	4.9 ± 0.3	0.7 ± 0.1	4.6 ± 0.2

Table S39: Mean ± standard deviation (in degrees) root-mean-square errors between tibio-femoral joint rotations estimated by the IMU-based systems and the optical reference on harness, calculated across all trials of knee 12.

2.13 Averaged plots for individual knee 13

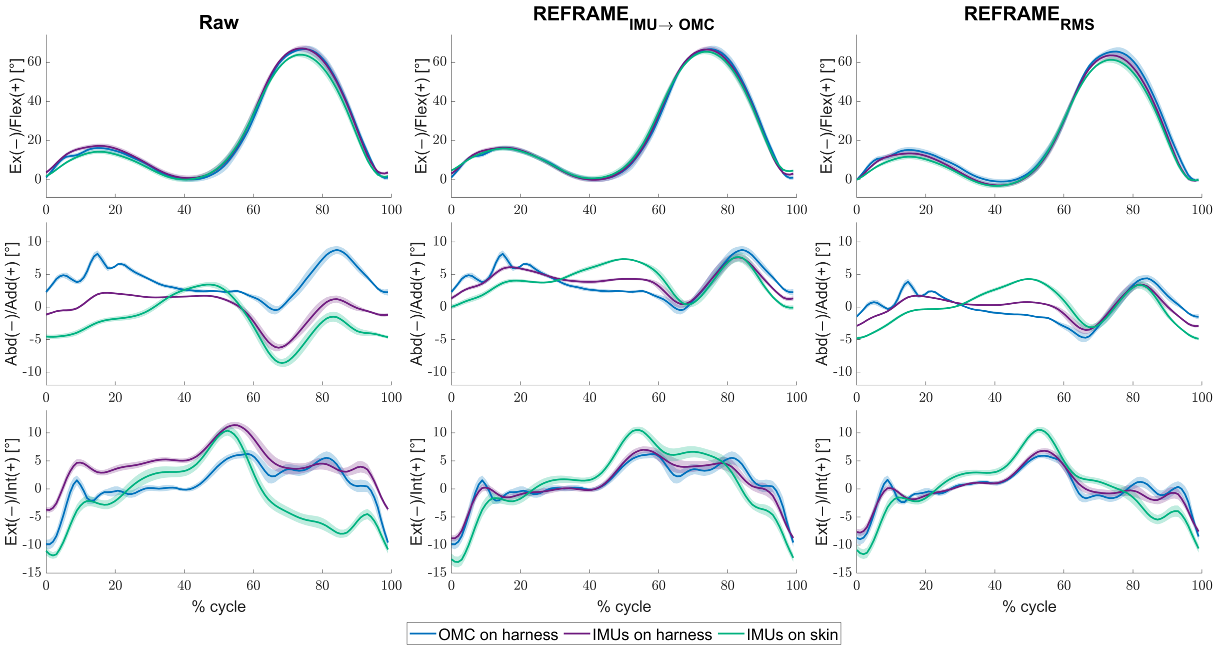

Figure S13: Mean tibio-femoral joint angles (solid lines) ± standard deviation (shaded areas), in degrees, as estimated by inertial measurement units (IMUs) on harness (purple), IMUs on skin (green), and optical motion capture (OMC) on harness (blue), averaged over all cycles for knee 13. Angles are shown as a percentage of the gait cycle under three conditions: 1) raw, i.e. in the absence of post-processing methods to correct reference frame orientation differences (left), 2) after implementation of REFRAME$_{IMU->OMC}$ (middle), and 3) after implementation of REFRAME$_{RMS}$ (right).

	Femur		Tibia	
	IMUs on harness	IMUs on skin	IMUs on harness	IMUs on skin
Rx	0.0 ± 0.0	0.0 ± 0.0	0.4 ± 0.1	-2.0 ± 0.2
Ry	-2.1 ± 0.2	-7.8 ± 1.0	0.1 ± 0.2	-4.6 ± 0.7
Rz	6.4 ± 0.1	11.0 ± 0.5	1.1 ± 0.2	9.1 ± 0.4

Table S40: Mean ± standard deviation (in degrees) of the rotational transformations (Rx: Rotation around X, Ry: Rotation around Y, Rz: Rotation around Z) applied to the femoral and tibial segment frames as part of $\text{REFRAME}_{IMU->OMC}$, calculated across all cycles of knee 13.

	Femur			Tibia		
	OMC on harness	IMUs on harness	IMUs on skin	OMC on harness	IMUs on harness	IMUs on skin
Rx	0.0 ± 0.0	0.0 ± 0.0	0.0 ± 0.0	1.0 ± 0.8	3.8 ± 0.8	2.8 ± 0.5
Ry	4.5 ± 0.9	2.7 ± 0.8	-1.8 ± 1.1	0.9 ± 0.9	1.2 ± 0.8	-1.5 ± 1.1
Rz	-3.2 ± 0.5	3.3 ± 0.5	7.4 ± 0.7	-2.1 ± 0.4	-0.7 ± 0.4	7.4 ± 0.6

Table S41: Mean ± standard deviation (in degrees) of the rotational transformations (Rx: Rotation around X, Ry: Rotation around Y, Rz: Rotation around Z) applied to the femoral and tibial segment frames as part of REFRAME_{RMS}, calculated across all cycles of knee 13.

	**Raw**		**$\text{REFRAME}_{IMU->OMC}$**		**REFRAME_{RMS}**	
	IMUs on harness	IMUs on skin	IMUs on harness	IMUs on skin	IMUs on harness	IMUs on skin
flexion/extension	1.5 ± 0.1	2.5 ± 0.1	1.3 ± 0.2	1.8 ± 0.1	2.2 ± 0.6	3.5 ± 0.6
abduction/adduction	4.7 ± 0.1	7.3 ± 0.3	1.3 ± 0.1	2.8 ± 0.1	1.4 ± 0.1	3.2 ± 0.1
external/internal rotation	4.2 ± 0.1	5.6 ± 0.2	1.0 ± 0.1	3.3 ± 0.2	1.0 ± 0.1	3.1 ± 0.2

Table S42: Mean ± standard deviation (in degrees) root-mean-square errors between tibio-femoral joint rotations estimated by the IMU-based systems and the optical reference on harness, calculated across all trials of knee 13.

2.14 Averaged plots for individual knee 14

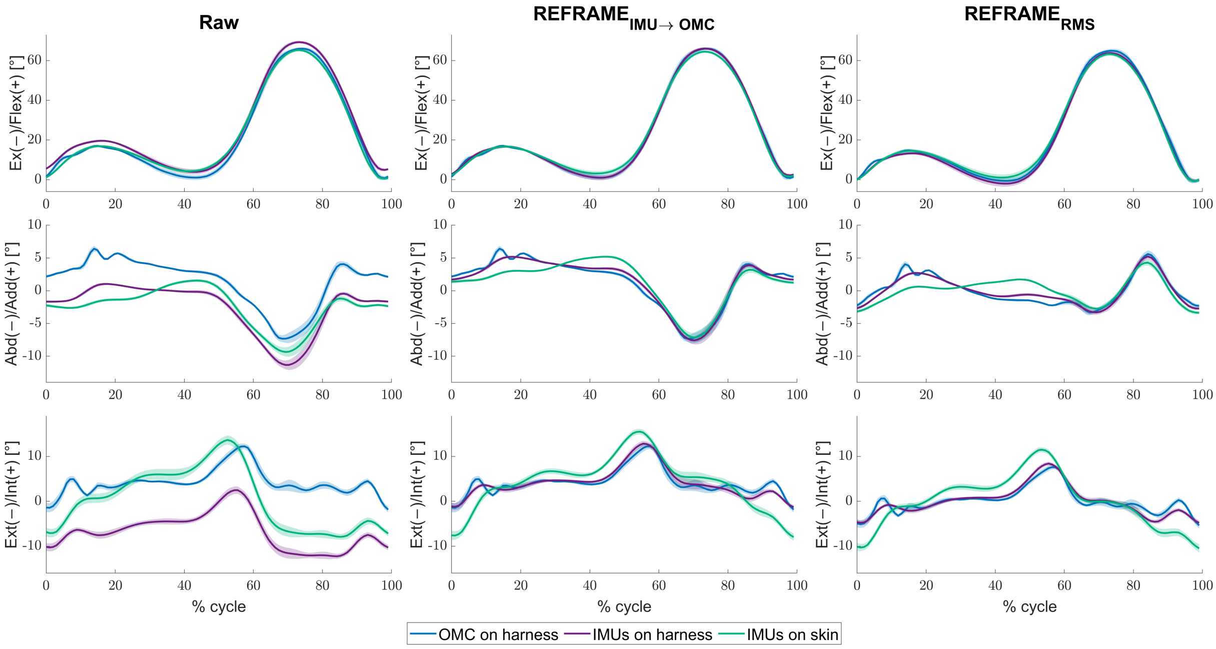

Figure S14: Mean tibio-femoral joint angles (solid lines) ± standard deviation (shaded areas), in degrees, as estimated by inertial measurement units (IMUs) on harness (purple), IMUs on skin (green), and optical motion capture (OMC) on harness (blue), averaged over all cycles for knee 14. Angles are shown as a percentage of the gait cycle under three conditions: 1) raw, i.e. in the absence of post-processing methods to correct reference frame orientation differences (left), 2) after implementation of REFRAME$_{IMU->OMC}$ (middle), and 3) after implementation of REFRAME$_{RMS}$ (right).

	Femur		Tibia	
	IMUs on harness	IMUs on skin	IMUs on harness	IMUs on skin
Rx	0.0 ± 0.0	0.0 ± 0.0	2.8 ± 0.1	-0.3 ± 0.1
Ry	-4.5 ± 0.2	-11.3 ± 0.5	-0.8 ± 0.2	-8.0 ± 0.5
Rz	3.5 ± 0.4	5.7 ± 0.7	12.2 ± 0.1	4.6 ± 0.2

Table S43: Mean ± standard deviation (in degrees) of the rotational transformations (Rx: Rotation around X, Ry: Rotation around Y, Rz: Rotation around Z) applied to the femoral and tibial segment frames as part of $\text{REFRAME}_{IMU->OMC}$, calculated across all cycles of knee 14.

	Femur			Tibia		
	OMC on harness	IMUs on harness	IMUs on skin	OMC on harness	IMUs on harness	IMUs on skin
Rx	0.0 ± 0.0	0.0 ± 0.0	0.0 ± 0.0	1.8 ± 0.6	6.0 ± 0.5	2.4 ± 0.5
Ry	4.6 ± 0.6	0.2 ± 0.7	-4.4 ± 0.9	0.3 ± 0.6	0.3 ± 0.6	-5.0 ± 0.9
Rz	6.8 ± 0.5	10.2 ± 0.6	11.1 ± 0.7	3.3 ± 0.7	15.5 ± 0.7	7.9 ± 0.6

Table S44: Mean ± standard deviation (in degrees) of the rotational transformations (Rx: Rotation around X, Ry: Rotation around Y, Rz: Rotation around Z) applied to the femoral and tibial segment frames as part of REFRAME_{RMS}, calculated across all cycles of knee 14.

	**Raw**		**$\text{REFRAME}_{IMU->OMC}$**		**REFRAME_{RMS}**	
	IMUs on harness	IMUs on skin	IMUs on harness	IMUs on skin	IMUs on harness	IMUs on skin
flexion/extension	3.1 ± 0.1	2.3 ± 0.1	0.9 ± 0.1	1.7 ± 0.1	1.6 ± 0.4	1.8 ± 0.1
abduction/adduction	4.0 ± 0.1	4.3 ± 0.1	0.5 ± 0.1	1.6 ± 0.1	0.6 ± 0.1	1.8 ± 0.1
external/internal rotation	11.2 ± 0.3	6.4 ± 0.1	1.0 ± 0.0	3.7 ± 0.2	1.0 ± 0.0	3.6 ± 0.2

Table S45: Mean ± standard deviation (in degrees) root-mean-square errors between tibio-femoral joint rotations estimated by the IMU-based systems and the optical reference on harness, calculated across all trials of knee 14.

2.15 Averaged plots for individual knee 15

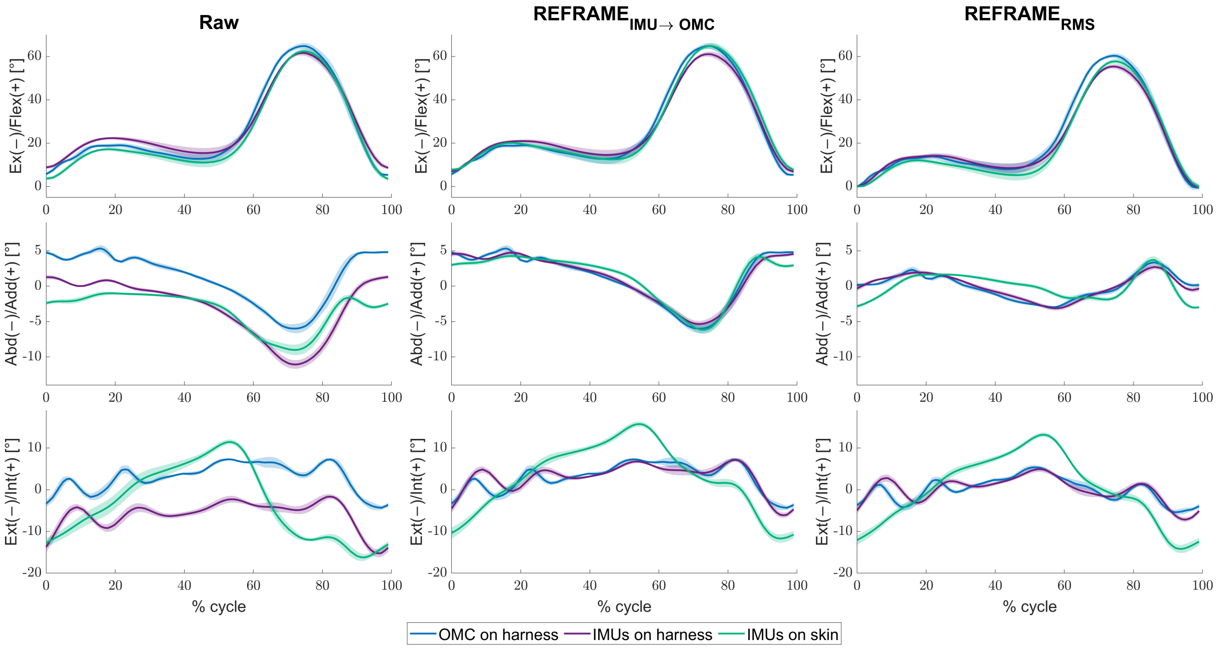

Figure S15: Mean tibio-femoral joint angles (solid lines) ± standard deviation (shaded areas), in degrees, as estimated by inertial measurement units (IMUs) on harness (purple), IMUs on skin (green), and optical motion capture (OMC) on harness (blue), averaged over all cycles for knee 15. Angles are shown as a percentage of the gait cycle under three conditions: 1) raw, i.e. in the absence of post-processing methods to correct reference frame orientation differences (left), 2) after implementation of REFRAME$_{IMU->OMC}$ (middle), and 3) after implementation of REFRAME$_{RMS}$ (right).

	Femur		Tibia	
	IMUs on harness	IMUs on skin	IMUs on harness	IMUs on skin
Rx	0.0 ± 0.0	0.0 ± 0.0	0.4 ± 0.1	-2.9 ± 0.2
Ry	1.9 ± 0.3	-11.3 ± 1.2	5.1 ± 0.3	-7.3 ± 1.5
Rz	1.8 ± 0.6	5.1 ± 1.2	11.3 ± 0.3	6.1 ± 0.5

Table S46: Mean ± standard deviation (in degrees) of the rotational transformations (Rx: Rotation around X, Ry: Rotation around Y, Rz: Rotation around Z) applied to the femoral and tibial segment frames as part of $\text{REFRAME}_{IMU->OMC}$, calculated across all cycles of knee 15.

	Femur			Tibia		
	OMC on harness	IMUs on harness	IMUs on skin	OMC on harness	IMUs on harness	IMUs on skin
Rx	0.0 ± 0.0	0.0 ± 0.0	0.0 ± 0.0	5.3 ± 0.8	6.0 ± 0.9	5.3 ± 0.9
Ry	10.2 ± 0.8	12.4 ± 0.9	-4.8 ± 1.2	6.0 ± 0.7	12.6 ± 0.8	-4.1 ± 1.3
Rz	4.6 ± 0.4	6.9 ± 0.6	11.4 ± 1.2	4.8 ± 0.4	16.0 ± 0.4	12.0 ± 0.7

Table S47: Mean ± standard deviation (in degrees) of the rotational transformations (Rx: Rotation around X, Ry: Rotation around Y, Rz: Rotation around Z) applied to the femoral and tibial segment frames as part of REFRAME_{RMS}, calculated across all cycles of knee 15.

	**Raw**		**REFRAME**$_{IMU->OMC}$		**REFRAME**$_{RMS}$	
	IMUs on harness	IMUs on skin	IMUs on harness	IMUs on skin	IMUs on harness	IMUs on skin
flexion/extension	3.2 ± 0.2	3.0 ± 0.2	2.7 ± 0.2	2.5 ± 0.4	3.1 ± 0.2	3.4 ± 0.5
abduction/adduction	4.3 ± 0.1	4.9 ± 0.1	0.5 ± 0.1	1.0 ± 0.2	0.5 ± 0.1	1.7 ± 0.1
external/internal rotation	9.1 ± 0.4	10.0 ± 0.5	1.7 ± 0.2	5.9 ± 0.2	1.6 ± 0.2	5.8 ± 0.2

Table S48: Mean ± standard deviation (in degrees) root-mean-square errors between tibio-femoral joint rotations estimated by the IMU-based systems and the optical reference on harness, calculated across all trials of knee 15.

2.16 Averaged plots for individual knee 16

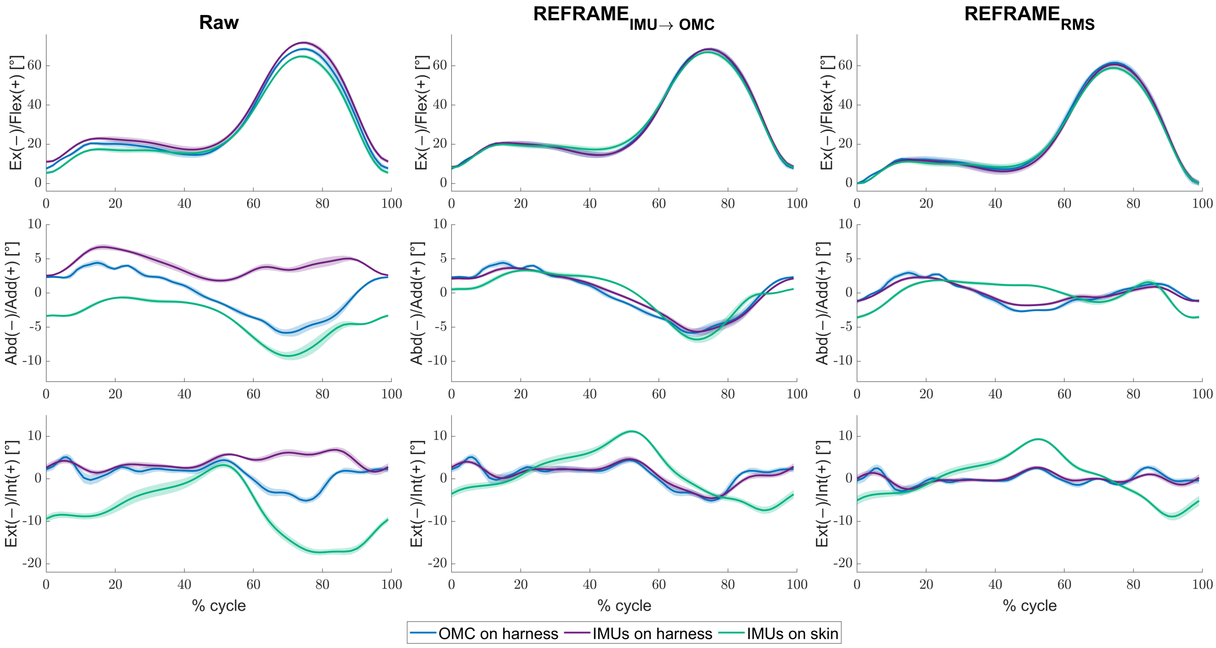

Figure S16: Mean tibio-femoral joint angles (solid lines) ± standard deviation (shaded areas), in degrees, as estimated by inertial measurement units (IMUs) on harness (purple), IMUs on skin (green), and optical motion capture (OMC) on harness (blue), averaged over all cycles for knee 16. Angles are shown as a percentage of the gait cycle under three conditions: 1) raw, i.e. in the absence of post-processing methods to correct reference frame orientation differences (left), 2) after implementation of REFRAME$_{IMU->OMC}$ (middle), and 3) after implementation of REFRAME$_{RMS}$ (right).

	Femur		Tibia	
	IMUs on harness	IMUs on skin	IMUs on harness	IMUs on skin
Rx	0.0 ± 0.0	0.0 ± 0.0	3.5 ± 0.1	-2.1 ± 0.2
Ry	2.6 ± 0.2	-7.2 ± 1.0	3.8 ± 0.2	-4.1 ± 1.1
Rz	-13.3 ± 0.2	3.1 ± 0.7	-12.6 ± 0.2	8.2 ± 0.4

Table S49: Mean ± standard deviation (in degrees) of the rotational transformations (Rx: Rotation around X, Ry: Rotation around Y, Rz: Rotation around Z) applied to the femoral and tibial segment frames as part of $\text{REFRAME}_{IMU->OMC}$, calculated across all cycles of knee 16.

	Femur			Tibia		
	OMC on harness	IMUs on harness	IMUs on skin	OMC on harness	IMUs on harness	IMUs on skin
Rx	0.0 ± 0.0	0.0 ± 0.0	0.0 ± 0.0	7.7 ± 0.7	10.9 ± 0.7	6.9 ± 0.6
Ry	0.2 ± 0.9	2.7 ± 1.0	-5.1 ± 1.4	-3.6 ± 0.9	-1.5 ± 0.9	-4.2 ± 1.5
Rz	10.8 ± 0.5	-2.6 ± 0.5	13.0 ± 1.1	8.5 ± 0.6	-4.5 ± 0.5	17.1 ± 0.7

Table S50: Mean ± standard deviation (in degrees) of the rotational transformations (Rx: Rotation around X, Ry: Rotation around Y, Rz: Rotation around Z) applied to the femoral and tibial segment frames as part of REFRAME_{RMS}, calculated across all cycles of knee 16.

	**Raw**		**REFRAME**$_{IMU->OMC}$		**REFRAME**$_{RMS}$	
	IMUs on harness	IMUs on skin	IMUs on harness	IMUs on skin	IMUs on harness	IMUs on skin
flexion/extension	3.0 ± 0.1	2.9 ± 0.1	1.0 ± 0.2	1.7 ± 0.2	1.3 ± 0.2	1.8 ± 0.2
abduction/adduction	5.1 ± 0.1	4.0 ± 0.1	0.6 ± 0.1	1.4 ± 0.1	0.5 ± 0.1	2.1 ± 0.1
external/internal rotation	4.6 ± 0.2	10.3 ± 0.3	0.8 ± 0.1	5.2 ± 0.2	0.7 ± 0.1	5.0 ± 0.2

Table S51: Mean ± standard deviation (in degrees) root-mean-square errors between tibio-femoral joint rotations estimated by the IMU-based systems and the optical reference on harness, calculated across all trials of knee 16.

2.17 Averaged plots for individual knee 18

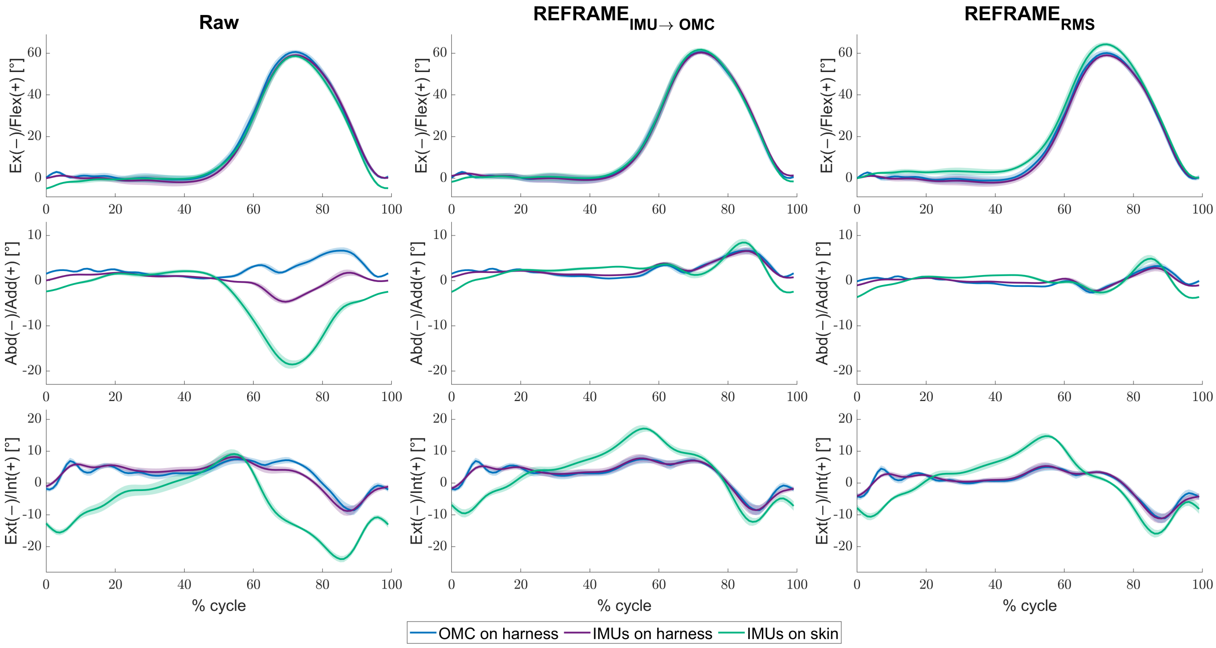

Figure S17: Mean tibio-femoral joint angles (solid lines) ± standard deviation (shaded areas), in degrees, as estimated by inertial measurement units (IMUs) on harness (purple), IMUs on skin (green), and optical motion capture (OMC) on harness (blue), averaged over all cycles for knee 18. Angles are shown as a percentage of the gait cycle under three conditions: 1) raw, i.e. in the absence of post-processing methods to correct reference frame orientation differences (left), 2) after implementation of REFRAME$_{IMU->OMC}$ (middle), and 3) after implementation of REFRAME$_{RMS}$ (right).

	Femur		Tibia	
	IMUs on harness	IMUs on skin	IMUs on harness	IMUs on skin
Rx	0.0 ± 0.0	0.0 ± 0.0	-1.2 ± 0.1	-1.5 ± 0.2
Ry	0.0 ± 0.1	-4.2 ± 0.8	0.5 ± 0.1	-3.8 ± 0.8
Rz	7.5 ± 0.1	24.1 ± 0.6	6.9 ± 0.1	30.3 ± 0.4

Table S52: Mean ± standard deviation (in degrees) of the rotational transformations (Rx: Rotation around X, Ry: Rotation around Y, Rz: Rotation around Z) applied to the femoral and tibial segment frames as part of $\text{REFRAME}_{IMU->OMC}$, calculated across all cycles of knee 18.

	Femur			Tibia		
	OMC on harness	IMUs on harness	IMUs on skin	OMC on harness	IMUs on harness	IMUs on skin
Rx	0.0 ± 0.0	0.0 ± 0.0	0.0 ± 0.0	0.3 ± 0.6	0.1 ± 0.4	-3.5 ± 0.4
Ry	-0.2 ± 1.0	-0.4 ± 0.9	0.1 ± 0.6	-1.9 ± 1.0	-1.5 ± 0.9	-1.8 ± 0.6
Rz	-3.2 ± 0.4	4.3 ± 0.4	18.9 ± 0.7	-5.6 ± 0.3	1.3 ± 0.3	23.9 ± 0.3

Table S53: Mean ± standard deviation (in degrees) of the rotational transformations (Rx: Rotation around X, Ry: Rotation around Y, Rz: Rotation around Z) applied to the femoral and tibial segment frames as part of REFRAME_{RMS}, calculated across all cycles of knee 18.

	**Raw**		**$\text{REFRAME}_{IMU->OMC}$**		**REFRAME_{RMS}**	
	IMUs on harness	IMUs on skin	IMUs on harness	IMUs on skin	IMUs on harness	IMUs on skin
flexion/extension	1.5 ± 0.1	2.5 ± 0.2	1.0 ± 0.1	1.2 ± 0.2	1.3 ± 0.1	3.3 ± 0.6
abduction/adduction	3.5 ± 0.1	10.1 ± 0.2	0.5 ± 0.0	1.8 ± 0.1	0.5 ± 0.0	1.6 ± 0.1
external/internal rotation	1.5 ± 0.1	12.1 ± 0.4	0.7 ± 0.1	5.9 ± 0.2	0.7 ± 0.1	5.8 ± 0.2

Table S54: Mean ± standard deviation (in degrees) root-mean-square errors between tibio-femoral joint rotations estimated by the IMU-based systems and the optical reference on harness, calculated across all trials of knee 18.

2.18 Averaged plots for individual knee 19

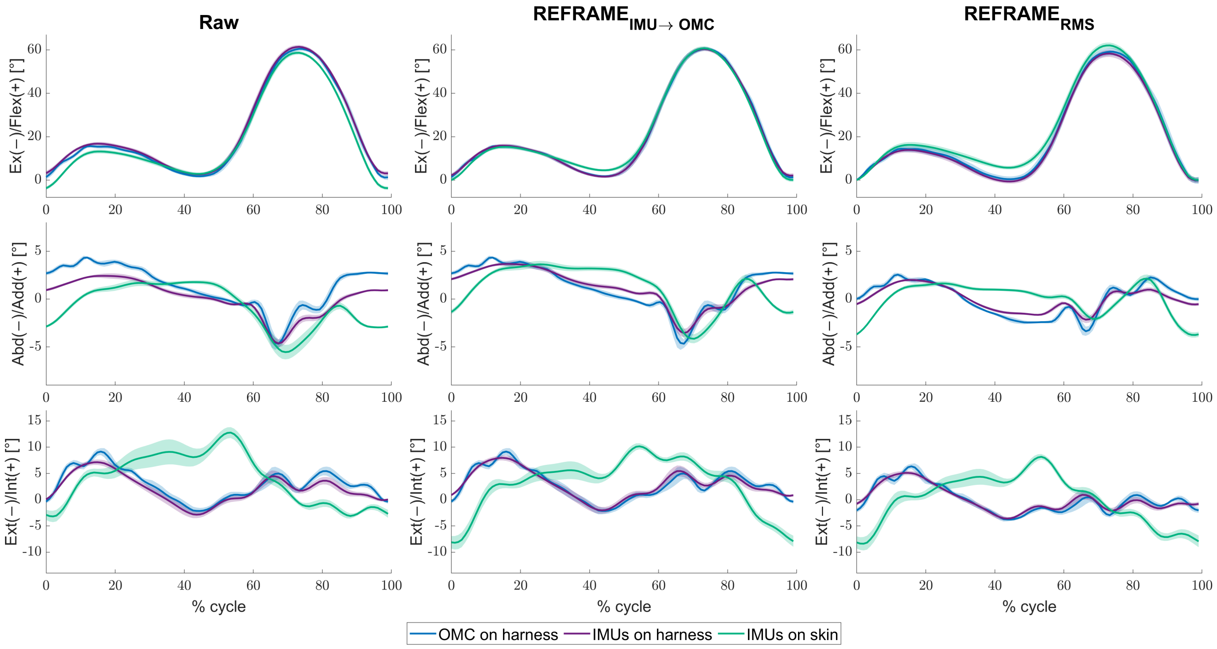

Figure S18: Mean tibio-femoral joint angles (solid lines) ± standard deviation (shaded areas), in degrees, as estimated by inertial measurement units (IMUs) on harness (purple), IMUs on skin (green), and optical motion capture (OMC) on harness (blue), averaged over all cycles for knee 19. Angles are shown as a percentage of the gait cycle under three conditions: 1) raw, i.e. in the absence of post-processing methods to correct reference frame orientation differences (left), 2) after implementation of REFRAME$_{IMU->OMC}$ (middle), and 3) after implementation of REFRAME$_{RMS}$ (right).

	Femur		Tibia	
	IMUs on harness	IMUs on skin	IMUs on harness	IMUs on skin
Rx	0.0 ± 0.0	0.0 ± 0.0	1.0 ± 0.1	-3.2 ± 0.2
Ry	-0.2 ± 0.1	-9.5 ± 1.3	1.0 ± 0.1	-8.2 ± 1.4
Rz	-0.1 ± 0.1	5.6 ± 0.7	0.7 ± 0.1	0.6 ± 0.5

Table S55: Mean ± standard deviation (in degrees) of the rotational transformations (Rx: Rotation around X, Ry: Rotation around Y, Rz: Rotation around Z) applied to the femoral and tibial segment frames as part of $\text{REFRAME}_{IMU->OMC}$, calculated across all cycles of knee 19.

	Femur			Tibia		
	OMC on harness	IMUs on harness	IMUs on skin	OMC on harness	IMUs on harness	IMUs on skin
Rx	0.0 ± 0.0	0.0 ± 0.0	0.0 ± 0.0	1.4 ± 1.2	3.2 ± 1.0	-3.3 ± 0.8
Ry	5.0 ± 0.6	4.9 ± 0.6	-1.1 ± 1.0	2.3 ± 0.6	3.5 ± 0.6	-2.1 ± 0.9
Rz	2.4 ± 0.4	2.2 ± 0.3	5.9 ± 0.7	0.8 ± 0.3	1.4 ± 0.3	0.8 ± 0.9

Table S56: Mean ± standard deviation (in degrees) of the rotational transformations (Rx: Rotation around X, Ry: Rotation around Y, Rz: Rotation around Z) applied to the femoral and tibial segment frames as part of REFRAME_{RMS}, calculated across all cycles of knee 19.

	**Raw**		**REFRAME**$_{IMU->OMC}$		**REFRAME**$_{RMS}$	
	IMUs on harness	IMUs on skin	IMUs on harness	IMUs on skin	IMUs on harness	IMUs on skin
flexion/extension	1.3 ± 0.1	3.1 ± 0.2	0.9 ± 0.2	1.7 ± 0.1	1.3 ± 0.4	3.2 ± 0.5
abduction/adduction	1.3 ± 0.0	3.0 ± 0.1	0.7 ± 0.0	2.1 ± 0.0	0.7 ± 0.0	2.1 ± 0.1
external/internal rotation	1.2 ± 0.1	6.5 ± 0.5	0.8 ± 0.1	6.0 ± 0.4	0.7 ± 0.1	5.8 ± 0.4

Table S57: Mean ± standard deviation (in degrees) root-mean-square errors between tibio-femoral joint rotations estimated by the IMU-based systems and the optical reference on harness, calculated across all trials of knee 19.

2.19 Averaged plots for individual knee 20

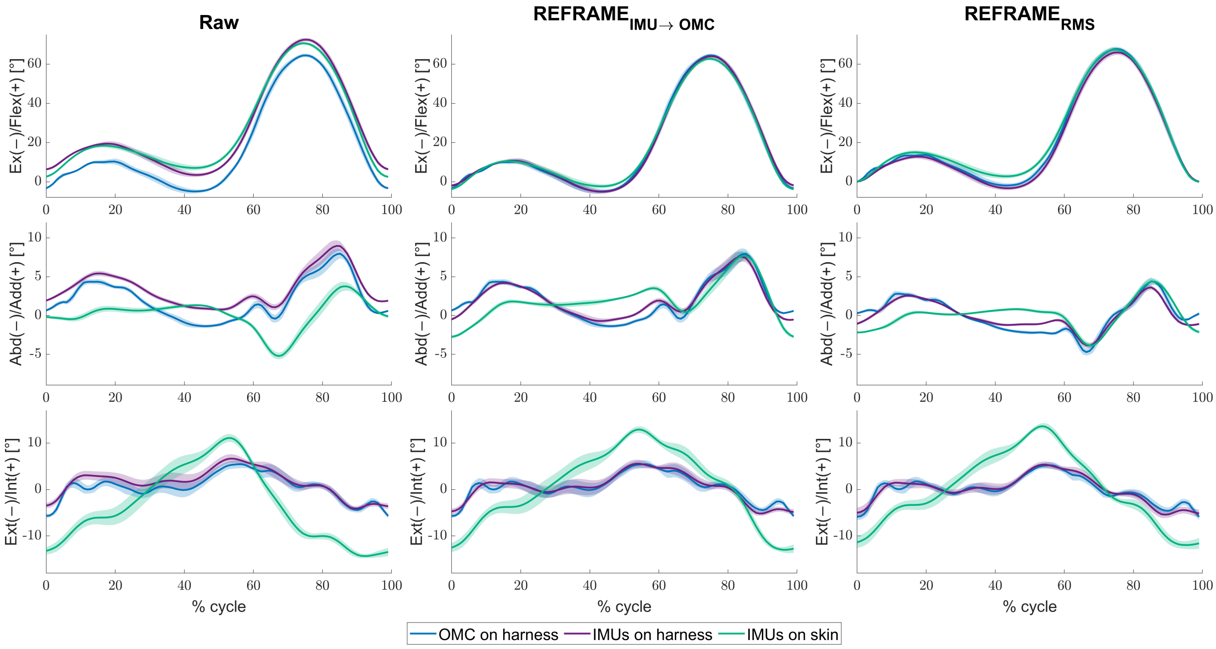

Figure S19: Mean tibio-femoral joint angles (solid lines) ± standard deviation (shaded areas), in degrees, as estimated by inertial measurement units (IMUs) on harness (purple), IMUs on skin (green), and optical motion capture (OMC) on harness (blue), averaged over all cycles for knee 20. Angles are shown as a percentage of the gait cycle under three conditions: 1) raw, i.e. in the absence of post-processing methods to correct reference frame orientation differences (left), 2) after implementation of REFRAME$_{IMU->OMC}$ (middle), and 3) after implementation of REFRAME$_{RMS}$ (right).

	Femur		Tibia	
	IMUs on harness	IMUs on skin	IMUs on harness	IMUs on skin
Rx	0.0 ± 0.0	0.0 ± 0.0	8.5 ± 0.1	8.4 ± 0.1
Ry	-1.2 ± 0.2	-5.9 ± 0.7	-3.0 ± 0.2	-6.0 ± 0.7
Rz	1.5 ± 0.1	10.7 ± 0.3	0.5 ± 0.1	11.8 ± 0.3

Table S58: Mean ± standard deviation (in degrees) of the rotational transformations (Rx: Rotation around X, Ry: Rotation around Y, Rz: Rotation around Z) applied to the femoral and tibial segment frames as part of $\text{REFRAME}_{IMU->OMC}$, calculated across all cycles of knee 20.

	Femur			Tibia		
	OMC on harness	IMUs on harness	IMUs on skin	OMC on harness	IMUs on harness	IMUs on skin
Rx	0.0 ± 0.0	0.0 ± 0.0	0.0 ± 0.0	-3.2 ± 0.4	6.6 ± 0.6	3.8 ± 0.5
Ry	-0.5 ± 0.8	-2.0 ± 0.9	-2.8 ± 1.3	-1.2 ± 0.8	-4.6 ± 0.9	-4.0 ± 1.2
Rz	-3.9 ± 0.5	-2.5 ± 0.5	4.8 ± 0.5	-4.1 ± 0.6	-3.9 ± 0.7	6.7 ± 0.9

Table S59: Mean ± standard deviation (in degrees) of the rotational transformations (Rx: Rotation around X, Ry: Rotation around Y, Rz: Rotation around Z) applied to the femoral and tibial segment frames as part of REFRAME_{RMS}, calculated across all cycles of knee 20.

	**Raw**		**REFRAME**$_{IMU->OMC}$		**REFRAME**$_{RMS}$	
	IMUs on harness	IMUs on skin	IMUs on harness	IMUs on skin	IMUs on harness	IMUs on skin
flexion/extension	8.7 ± 0.1	8.7 ± 0.2	1.1 ± 0.2	1.6 ± 0.1	1.7 ± 0.2	2.5 ± 0.2
abduction/adduction	1.5 ± 0.1	3.3 ± 0.1	0.7 ± 0.1	2.3 ± 0.1	0.8 ± 0.1	1.9 ± 0.1
external/internal rotation	1.3 ± 0.1	7.4 ± 0.2	0.7 ± 0.1	5.6 ± 0.2	0.8 ± 0.1	5.6 ± 0.2

Table S60: Mean ± standard deviation (in degrees) root-mean-square errors between tibio-femoral joint rotations estimated by the IMU-based systems and the optical reference on harness, calculated across all trials of knee 20.

2.20 Averaged plots for individual knee 21

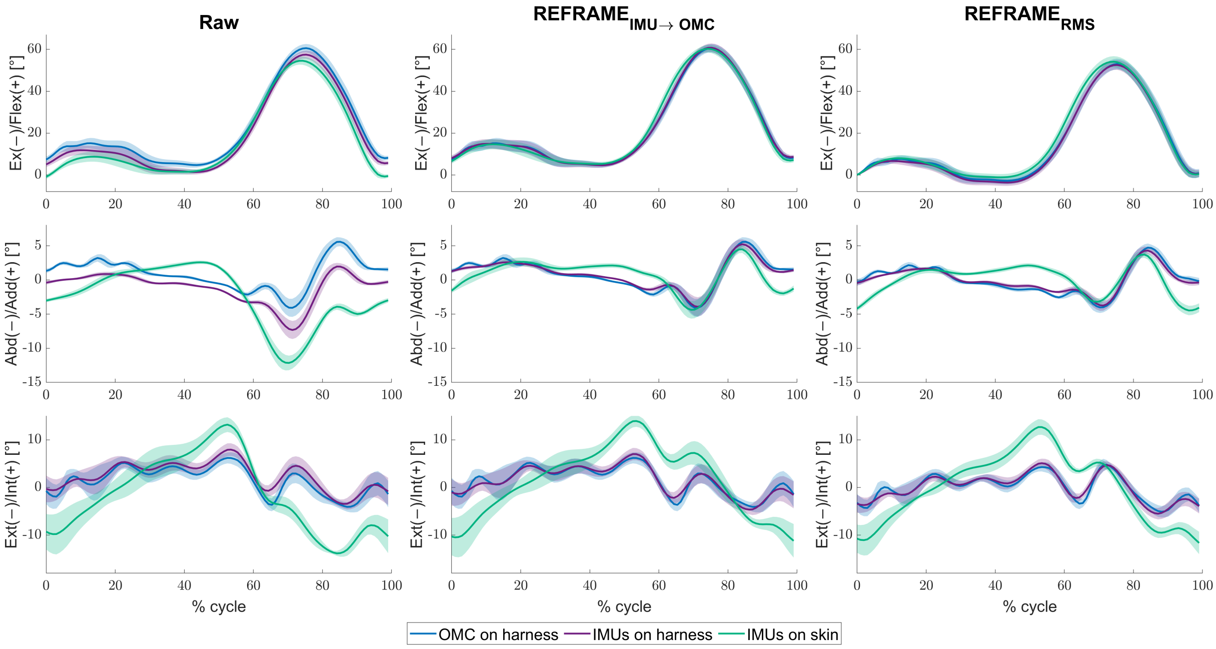

Figure S20: Mean tibio-femoral joint angles (solid lines) ± standard deviation (shaded areas), in degrees, as estimated by inertial measurement units (IMUs) on harness (purple), IMUs on skin (green), and optical motion capture (OMC) on harness (blue), averaged over all cycles for knee 21. Angles are shown as a percentage of the gait cycle under three conditions: 1) raw, i.e. in the absence of post-processing methods to correct reference frame orientation differences (left), 2) after implementation of REFRAME$_{IMU->OMC}$ (middle), and 3) after implementation of REFRAME$_{RMS}$ (right).

	Femur		Tibia	
	IMUs on harness	IMUs on skin	IMUs on harness	IMUs on skin
Rx	0.0 ± 0.0	0.0 ± 0.0	-3.0 ± 0.1	-5.4 ± 0.3
Ry	1.8 ± 0.2	-8.8 ± 1.1	3.3 ± 0.2	-10.0 ± 1.3
Rz	1.6 ± 0.2	14.9 ± 0.9	1.1 ± 0.2	13.3 ± 0.7

Table S61: Mean ± standard deviation (in degrees) of the rotational transformations (Rx: Rotation around X, Ry: Rotation around Y, Rz: Rotation around Z) applied to the femoral and tibial segment frames as part of $\text{REFRAME}_{IMU->OMC}$, calculated across all cycles of knee 21.

	Femur			Tibia		
	OMC on harness	IMUs on harness	IMUs on skin	OMC on harness	IMUs on harness	IMUs on skin
Rx	0.0 ± 0.0	0.0 ± 0.0	0.0 ± 0.0	7.3 ± 1.4	5.0 ± 1.3	1.3 ± 1.1
Ry	-3.2 ± 3.0	-1.2 ± 2.9	-6.2 ± 4.5	-4.8 ± 3.1	-1.2 ± 3.1	-7.1 ± 4.6
Rz	3.1 ± 1.9	4.7 ± 1.7	15.8 ± 2.4	0.8 ± 0.8	1.6 ± 0.7	14.5 ± 0.9

Table S62: Mean ± standard deviation (in degrees) of the rotational transformations (Rx: Rotation around X, Ry: Rotation around Y, Rz: Rotation around Z) applied to the femoral and tibial segment frames as part of REFRAME_{RMS}, calculated across all cycles of knee 21.

	**Raw**		**REFRAME**$_{IMU->OMC}$		**REFRAME**$_{RMS}$	
	IMUs on harness	IMUs on skin	IMUs on harness	IMUs on skin	IMUs on harness	IMUs on skin
flexion/extension	3.2 ± 0.1	5.9 ± 0.3	0.7 ± 0.1	2.0 ± 0.2	1.1 ± 0.3	2.7 ± 0.7
abduction/adduction	2.3 ± 0.1	5.4 ± 0.2	0.4 ± 0.0	1.8 ± 0.2	0.4 ± 0.0	2.5 ± 0.2
external/internal rotation	1.3 ± 0.1	6.8 ± 0.5	0.8 ± 0.1	5.9 ± 0.5	0.7 ± 0.1	5.5 ± 0.4

Table S63: Mean ± standard deviation (in degrees) root-mean-square errors between tibio-femoral joint rotations estimated by the IMU-based systems and the optical reference on harness, calculated across all trials of knee 21.

2.21 Averaged plots for individual knee 22

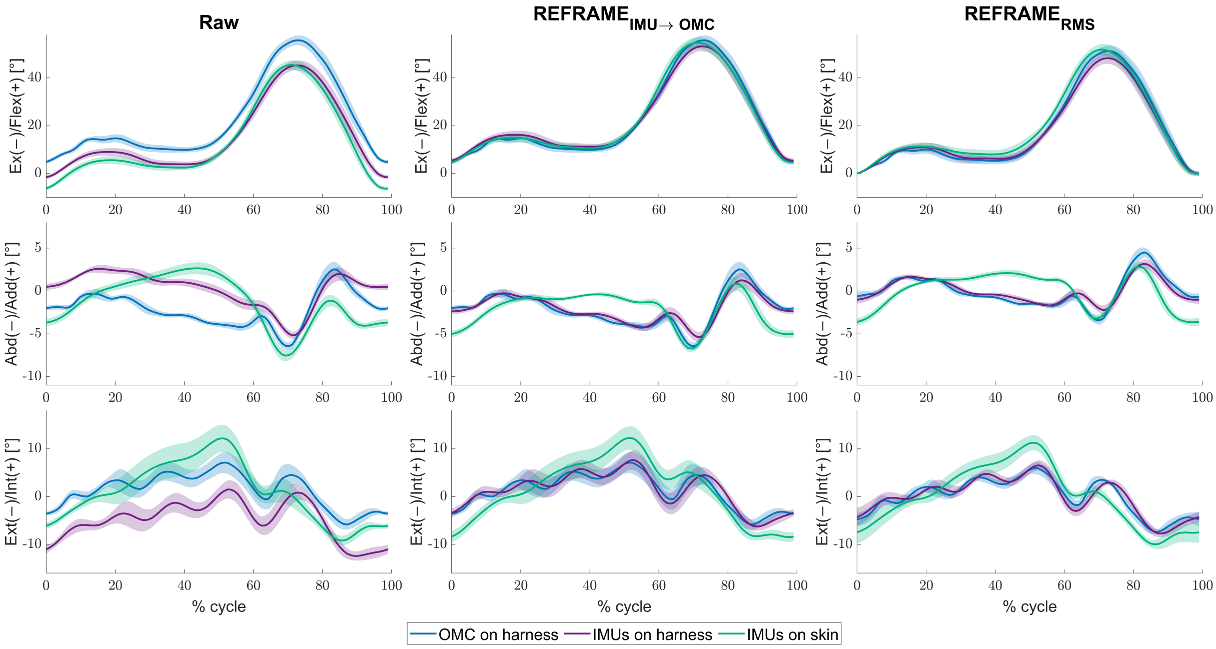

Figure S21: Mean tibio-femoral joint angles (solid lines) ± standard deviation (shaded areas), in degrees, as estimated by inertial measurement units (IMUs) on harness (purple), IMUs on skin (green), and optical motion capture (OMC) on harness (blue), averaged over all cycles for knee 22. Angles are shown as a percentage of the gait cycle under three conditions: 1) raw, i.e. in the absence of post-processing methods to correct reference frame orientation differences (left), 2) after implementation of REFRAME$_{IMU->OMC}$ (middle), and 3) after implementation of REFRAME$_{RMS}$ (right).

	Femur		Tibia	
	IMUs on harness	IMUs on skin	IMUs on harness	IMUs on skin
Rx	0.0 ± 0.0	0.0 ± 0.0	-7.3 ± 0.1	-9.4 ± 0.4
Ry	5.9 ± 0.7	-5.1 ± 2.0	1.5 ± 0.6	-8.1 ± 2.0
Rz	2.5 ± 0.4	7.4 ± 0.8	10.5 ± 0.3	5.4 ± 0.6

Table S64: Mean ± standard deviation (in degrees) of the rotational transformations (Rx: Rotation around X, Ry: Rotation around Y, Rz: Rotation around Z) applied to the femoral and tibial segment frames as part of REFRAME$_{IMU->OMC}$, calculated across all cycles of knee 22.

	Femur			Tibia		
	OMC on harness	IMUs on harness	IMUs on skin	OMC on harness	IMUs on harness	IMUs on skin
Rx	0.0 ± 0.0	0.0 ± 0.0	0.0 ± 0.0	4.7 ± 0.8	-2.6 ± 0.7	-5.9 ± 0.8
Ry	0.7 ± 2.3	7.6 ± 2.7	0.8 ± 3.3	2.4 ± 2.5	5.6 ± 3.0	0.2 ± 3.5
Rz	1.0 ± 1.1	3.3 ± 1.2	6.0 ± 1.6	-0.3 ± 1.2	10.1 ± 1.2	5.0 ± 1.6

Table S65: Mean ± standard deviation (in degrees) of the rotational transformations (Rx: Rotation around X, Ry: Rotation around Y, Rz: Rotation around Z) applied to the femoral and tibial segment frames as part of REFRAME$_{RMS}$, calculated across all cycles of knee 22.

	**Raw**		**REFRAME$_{IMU->OMC}$**		**REFRAME$_{RMS}$**	
	IMUs on harness	IMUs on skin	IMUs on harness	IMUs on skin	IMUs on harness	IMUs on skin
flexion/extension	7.5 ± 0.1	9.5 ± 0.2	1.7 ± 0.1	1.6 ± 0.2	1.8 ± 0.1	2.5 ± 0.4
abduction/adduction	2.8 ± 0.1	3.4 ± 0.2	0.7 ± 0.1	2.1 ± 0.2	0.7 ± 0.1	2.2 ± 0.1
external/internal rotation	6.4 ± 0.3	3.5 ± 0.5	1.1 ± 0.1	3.4 ± 0.5	1.1 ± 0.1	3.3 ± 0.5

Table S66: Mean ± standard deviation (in degrees) root-mean-square errors between tibio-femoral joint rotations estimated by the IMU-based systems and the optical reference on harness, calculated across all trials of knee 22.

2.22 Averaged plots for individual knee 23

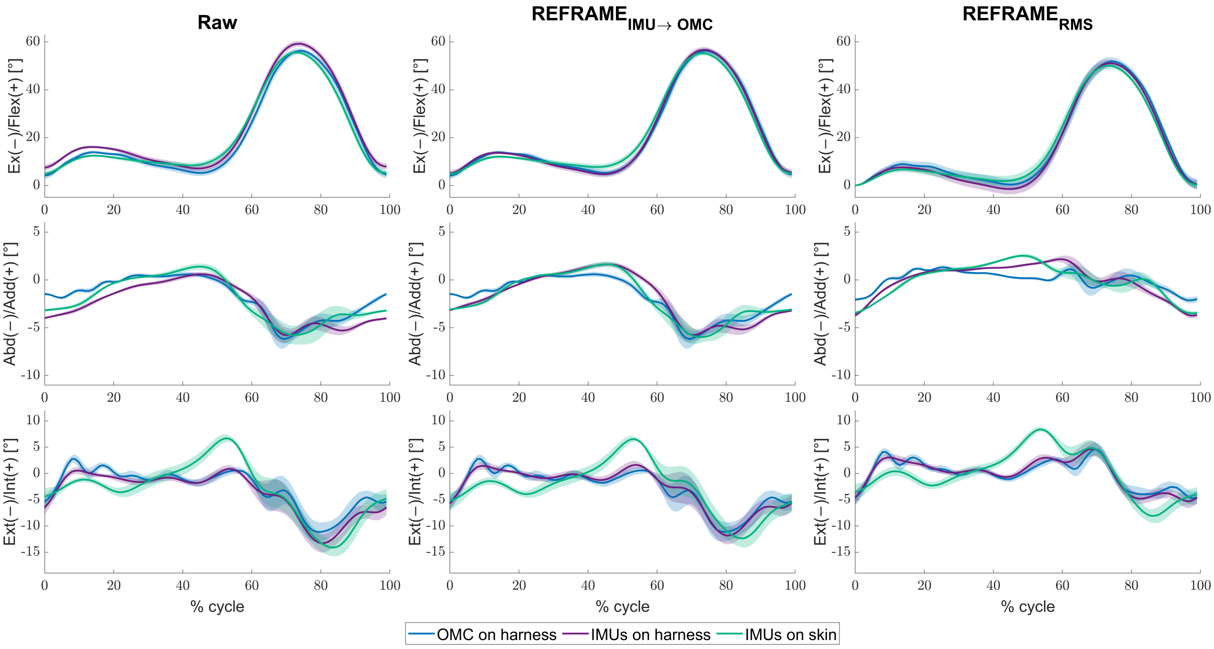

Figure S22: Mean tibio-femoral joint angles (solid lines) ± standard deviation (shaded areas), in degrees, as estimated by inertial measurement units (IMUs) on harness (purple), IMUs on skin (green), and optical motion capture (OMC) on harness (blue), averaged over all cycles for knee 23. Angles are shown as a percentage of the gait cycle under three conditions: 1) raw, i.e. in the absence of post-processing methods to correct reference frame orientation differences (left), 2) after implementation of REFRAME$_{IMU->OMC}$ (middle), and 3) after implementation of REFRAME$_{RMS}$ (right).

	Femur		Tibia	
	IMUs on harness	IMUs on skin	IMUs on harness	IMUs on skin
Rx	0.0 ± 0.0	0.0 ± 0.0	2.4 ± 0.1	0.5 ± 0.1
Ry	-1.2 ± 0.3	-3.8 ± 1.3	-0.0 ± 0.3	-3.8 ± 1.3
Rz	-0.5 ± 0.2	1.8 ± 0.3	0.0 ± 0.2	0.7 ± 0.6

Table S67: Mean ± standard deviation (in degrees) of the rotational transformations (Rx: Rotation around X, Ry: Rotation around Y, Rz: Rotation around Z) applied to the femoral and tibial segment frames as part of $\text{REFRAME}_{IMU->OMC}$, calculated across all cycles of knee 23.

	Femur			Tibia		
	OMC on harness	IMUs on harness	IMUs on skin	OMC on harness	IMUs on harness	IMUs on skin
Rx	0.0 ± 0.0	0.0 ± 0.0	0.0 ± 0.0	4.9 ± 1.6	8.5 ± 1.4	6.0 ± 1.3
Ry	-4.6 ± 2.3	-5.6 ± 2.2	-6.4 ± 2.4	-4.7 ± 2.3	-4.3 ± 2.2	-6.2 ± 2.3
Rz	9.8 ± 0.9	9.3 ± 0.9	10.4 ± 1.0	10.5 ± 0.8	10.7 ± 1.0	11.0 ± 0.9

Table S68: Mean ± standard deviation (in degrees) of the rotational transformations (Rx: Rotation around X, Ry: Rotation around Y, Rz: Rotation around Z) applied to the femoral and tibial segment frames as part of REFRAME_{RMS}, calculated across all cycles of knee 23.

	**Raw**		**$\text{REFRAME}_{IMU->OMC}$**		**REFRAME_{RMS}**	
	IMUs on harness	IMUs on skin	IMUs on harness	IMUs on skin	IMUs on harness	IMUs on skin
flexion/extension	2.7 ± 0.1	2.7 ± 0.2	1.0 ± 0.2	2.3 ± 0.2	1.5 ± 0.2	2.3 ± 0.2
abduction/adduction	1.2 ± 0.0	0.9 ± 0.1	0.9 ± 0.0	0.8 ± 0.0	1.0 ± 0.0	1.1 ± 0.1
external/internal rotation	1.4 ± 0.1	3.1 ± 0.1	0.9 ± 0.1	3.1 ± 0.1	0.8 ± 0.1	3.2 ± 0.1

Table S69: Mean ± standard deviation (in degrees) root-mean-square errors between tibio-femoral joint rotations estimated by the IMU-based systems and the optical reference on harness, calculated across all trials of knee 23.

2.23 Averaged plots for individual knee 24

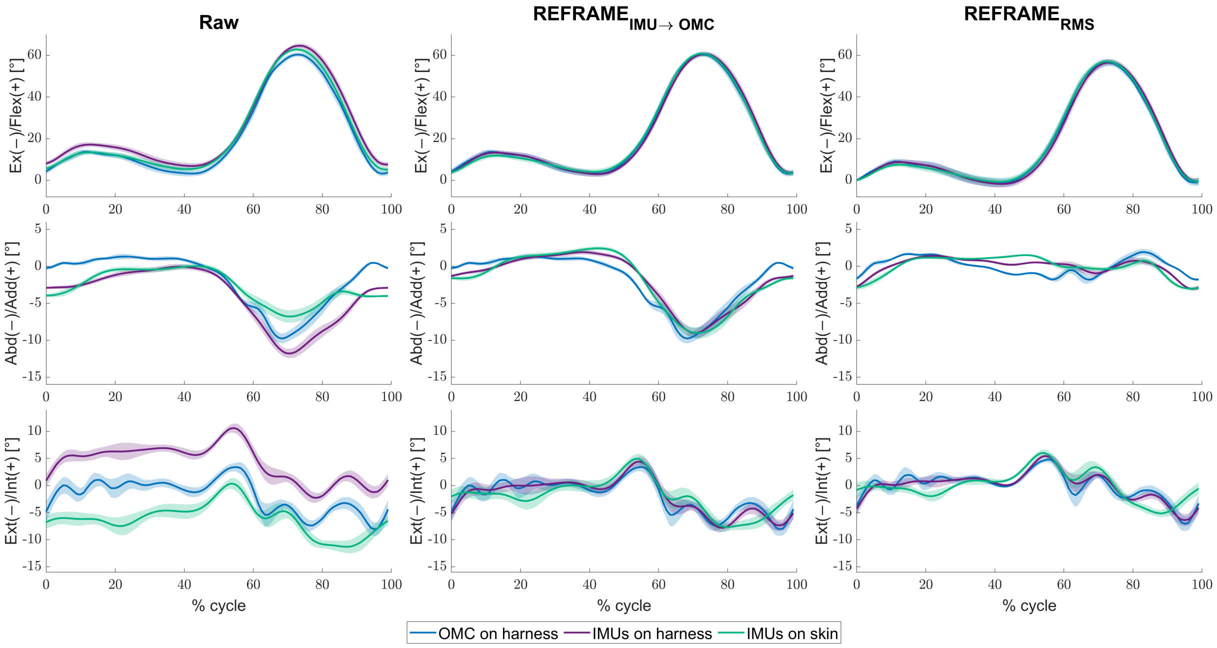

Figure S23: Mean tibio-femoral joint angles (solid lines) ± standard deviation (shaded areas), in degrees, as estimated by inertial measurement units (IMUs) on harness (purple), IMUs on skin (green), and optical motion capture (OMC) on harness (blue), averaged over all cycles for knee 24. Angles are shown as a percentage of the gait cycle under three conditions: 1) raw, i.e. in the absence of post-processing methods to correct reference frame orientation differences (left), 2) after implementation of REFRAME$_{IMU->OMC}$ (middle), and 3) after implementation of REFRAME$_{RMS}$ (right).

	Femur		Tibia	
	IMUs on harness	IMUs on skin	IMUs on harness	IMUs on skin
Rx	0.0 ± 0.0	0.0 ± 0.0	4.1 ± 0.1	1.4 ± 0.2
Ry	0.5 ± 0.3	-0.6 ± 0.4	2.0 ± 0.3	2.3 ± 0.5
Rz	1.1 ± 0.1	-5.4 ± 0.4	-5.2 ± 0.2	-0.8 ± 0.3

Table S70: Mean ± standard deviation (in degrees) of the rotational transformations (Rx: Rotation around X, Ry: Rotation around Y, Rz: Rotation around Z) applied to the femoral and tibial segment frames as part of $\text{REFRAME}_{IMU->OMC}$, calculated across all cycles of knee 24.

	Femur			Tibia		
	OMC on harness	IMUs on harness	IMUs on skin	OMC on harness	IMUs on harness	IMUs on skin
Rx	0.0 ± 0.0	0.0 ± 0.0	0.0 ± 0.0	4.5 ± 1.3	8.7 ± 1.0	5.7 ± 1.1
Ry	1.2 ± 1.1	1.0 ± 1.0	0.6 ± 1.2	0.1 ± 1.2	1.0 ± 1.1	2.4 ± 1.3
Rz	11.2 ± 0.7	12.4 ± 0.7	5.8 ± 0.7	12.2 ± 0.5	6.8 ± 0.5	11.3 ± 0.6

Table S71: Mean ± standard deviation (in degrees) of the rotational transformations (Rx: Rotation around X, Ry: Rotation around Y, Rz: Rotation around Z) applied to the femoral and tibial segment frames as part of REFRAME_{RMS}, calculated across all cycles of knee 24.

	**Raw**		**REFRAME$_{IMU->OMC}$**		**REFRAME$_{RMS}$**	
	IMUs on harness	IMUs on skin	IMUs on harness	IMUs on skin	IMUs on harness	IMUs on skin
flexion/extension	4.2 ± 0.1	2.1 ± 0.2	1.4 ± 0.2	1.2 ± 0.1	1.6 ± 0.2	1.3 ± 0.2
abduction/adduction	2.4 ± 0.0	2.3 ± 0.1	1.2 ± 0.1	1.4 ± 0.1	1.1 ± 0.1	1.5 ± 0.1
external/internal rotation	6.2 ± 0.1	4.9 ± 0.3	1.0 ± 0.1	2.2 ± 0.2	0.9 ± 0.1	2.2 ± 0.2

Table S72: Mean ± standard deviation (in degrees) root-mean-square errors between tibio-femoral joint rotations estimated by the IMU-based systems and the optical reference on harness, calculated across all trials of knee 24.

2.24 Averaged plots for individual knee 25

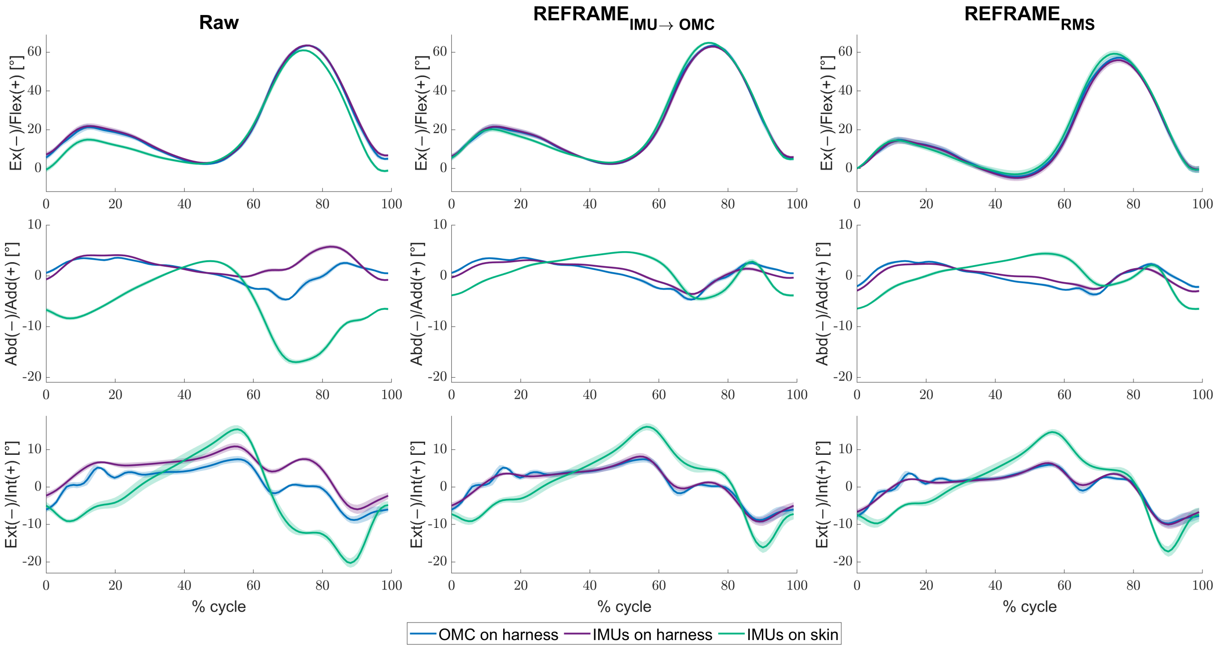

Figure S24: Mean tibio-femoral joint angles (solid lines) ± standard deviation (shaded areas), in degrees, as estimated by inertial measurement units (IMUs) on harness (purple), IMUs on skin (green), and optical motion capture (OMC) on harness (blue), averaged over all cycles for knee 25. Angles are shown as a percentage of the gait cycle under three conditions: 1) raw, i.e. in the absence of post-processing methods to correct reference frame orientation differences (left), 2) after implementation of $\text{REFRAME}_{IMU->OMC}$ (middle), and 3) after implementation of REFRAME_{RMS} (right).

	Femur		Tibia	
	IMUs on harness	IMUs on skin	IMUs on harness	IMUs on skin
Rx	0.0 ± 0.0	0.0 ± 0.0	0.9 ± 0.1	-3.7 ± 0.3
Ry	-0.5 ± 0.2	-11.2 ± 0.9	0.7 ± 0.1	-10.2 ± 0.9
Rz	-7.2 ± 0.2	18.8 ± 0.4	-9.9 ± 0.2	16.3 ± 0.6

Table S73: Mean ± standard deviation (in degrees) of the rotational transformations (Rx: Rotation around X, Ry: Rotation around Y, Rz: Rotation around Z) applied to the femoral and tibial segment frames as part of REFRAME$_{IMU->OMC}$, calculated across all cycles of knee 25.

	Femur			Tibia		
	OMC on harness	IMUs on harness	IMUs on skin	OMC on harness	IMUs on harness	IMUs on skin
Rx	0.0 ± 0.0	0.0 ± 0.0	0.0 ± 0.0	6.2 ± 1.3	7.2 ± 1.3	2.5 ± 1.3
Ry	-1.6 ± 0.8	-2.5 ± 0.9	-9.2 ± 2.3	-3.6 ± 0.8	-4.4 ± 1.0	-8.3 ± 2.2
Rz	4.7 ± 0.4	-2.4 ± 0.4	22.3 ± 0.9	3.1 ± 0.4	-6.8 ± 0.4	20.2 ± 0.4

Table S74: Mean ± standard deviation (in degrees) of the rotational transformations (Rx: Rotation around X, Ry: Rotation around Y, Rz: Rotation around Z) applied to the femoral and tibial segment frames as part of REFRAME$_{RMS}$, calculated across all cycles of knee 25.

	**Raw**		**REFRAME$_{IMU->OMC}$**		**REFRAME$_{RMS}$**	
	IMUs on harness	IMUs on skin	IMUs on harness	IMUs on skin	IMUs on harness	IMUs on skin
flexion/extension	1.1 ± 0.1	4.6 ± 0.2	0.9 ± 0.1	1.7 ± 0.2	1.2 ± 0.2	1.9 ± 0.4
abduction/adduction	2.6 ± 0.1	8.8 ± 0.1	0.8 ± 0.0	3.2 ± 0.1	0.9 ± 0.1	3.8 ± 0.1
external/internal rotation	3.7 ± 0.1	7.6 ± 0.3	0.8 ± 0.0	5.8 ± 0.2	0.8 ± 0.0	5.3 ± 0.2

Table S75: Mean ± standard deviation (in degrees) root-mean-square errors between tibio-femoral joint rotations estimated by the IMU-based systems and the optical reference on harness, calculated across all trials of knee 25.

2.25 Averaged plots for individual knee 26

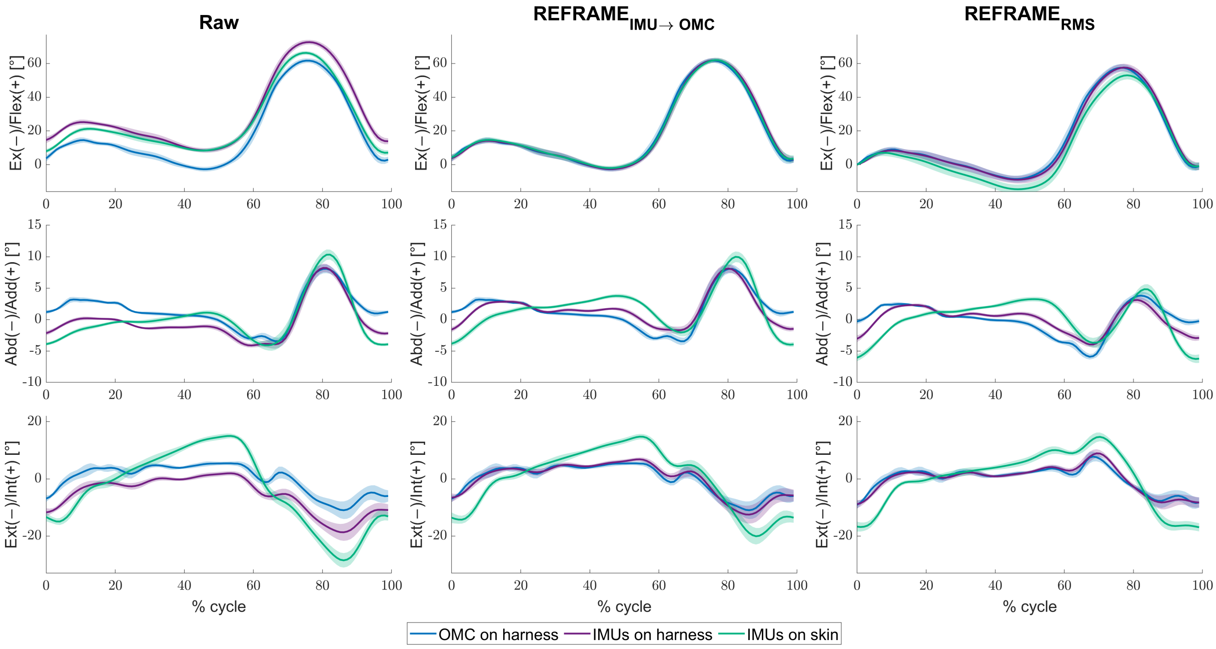

Figure S25: Mean tibio-femoral joint angles (solid lines) ± standard deviation (shaded areas), in degrees, as estimated by inertial measurement units (IMUs) on harness (purple), IMUs on skin (green), and optical motion capture (OMC) on harness (blue), averaged over all cycles for knee 26. Angles are shown as a percentage of the gait cycle under three conditions: 1) raw, i.e. in the absence of post-processing methods to correct reference frame orientation differences (left), 2) after implementation of REFRAME$_{IMU->OMC}$ (middle), and 3) after implementation of REFRAME$_{RMS}$ (right).

	Femur		Tibia	
	IMUs on harness	IMUs on skin	IMUs on harness	IMUs on skin
Rx	0.0 ± 0.0	0.0 ± 0.0	11.0 ± 0.1	7.1 ± 0.5
Ry	-2.6 ± 0.4	-13.6 ± 0.6	-0.0 ± 0.4	-12.8 ± 0.7
Rz	2.0 ± 0.2	8.0 ± 0.7	6.3 ± 0.2	6.8 ± 0.6

Table S76: Mean ± standard deviation (in degrees) of the rotational transformations (Rx: Rotation around X, Ry: Rotation around Y, Rz: Rotation around Z) applied to the femoral and tibial segment frames as part of $\text{REFRAME}_{IMU->OMC}$, calculated across all cycles of knee 26.

	Femur			Tibia		
	OMC on harness	IMUs on harness	IMUs on skin	OMC on harness	IMUs on harness	IMUs on skin
Rx	0.0 ± 0.0	0.0 ± 0.0	0.0 ± 0.0	5.0 ± 2.0	17.2 ± 1.9	16.9 ± 2.3
Ry	-9.4 ± 1.5	-13.3 ± 1.2	-30.3 ± 2.0	-10.3 ± 1.5	-11.0 ± 1.2	-29.3 ± 1.9
Rz	1.2 ± 0.7	3.5 ± 0.9	11.7 ± 1.0	-0.8 ± 0.8	5.7 ± 1.1	10.3 ± 1.4

Table S77: Mean ± standard deviation (in degrees) of the rotational transformations (Rx: Rotation around X, Ry: Rotation around Y, Rz: Rotation around Z) applied to the femoral and tibial segment frames as part of REFRAME_{RMS}, calculated across all cycles of knee 26.

	**Raw**		**REFRAME**$_{IMU->OMC}$		**REFRAME**$_{RMS}$	
	IMUs on harness	IMUs on skin	IMUs on harness	IMUs on skin	IMUs on harness	IMUs on skin
flexion/extension	11.2 ± 0.1	7.4 ± 0.3	1.6 ± 0.2	1.3 ± 0.2	1.8 ± 0.2	5.3 ± 0.8
abduction/adduction	2.1 ± 0.0	2.7 ± 0.1	1.3 ± 0.1	2.8 ± 0.1	1.4 ± 0.1	3.2 ± 0.2
external/internal rotation	5.7 ± 0.2	9.6 ± 0.3	1.2 ± 0.1	6.7 ± 0.3	1.0 ± 0.1	6.3 ± 0.3

Table S78: Mean ± standard deviation (in degrees) root-mean-square errors between tibio-femoral joint rotations estimated by the IMU-based systems and the optical reference on harness, calculated across all trials of knee 26.

2.26 Averaged plots for individual knee 27

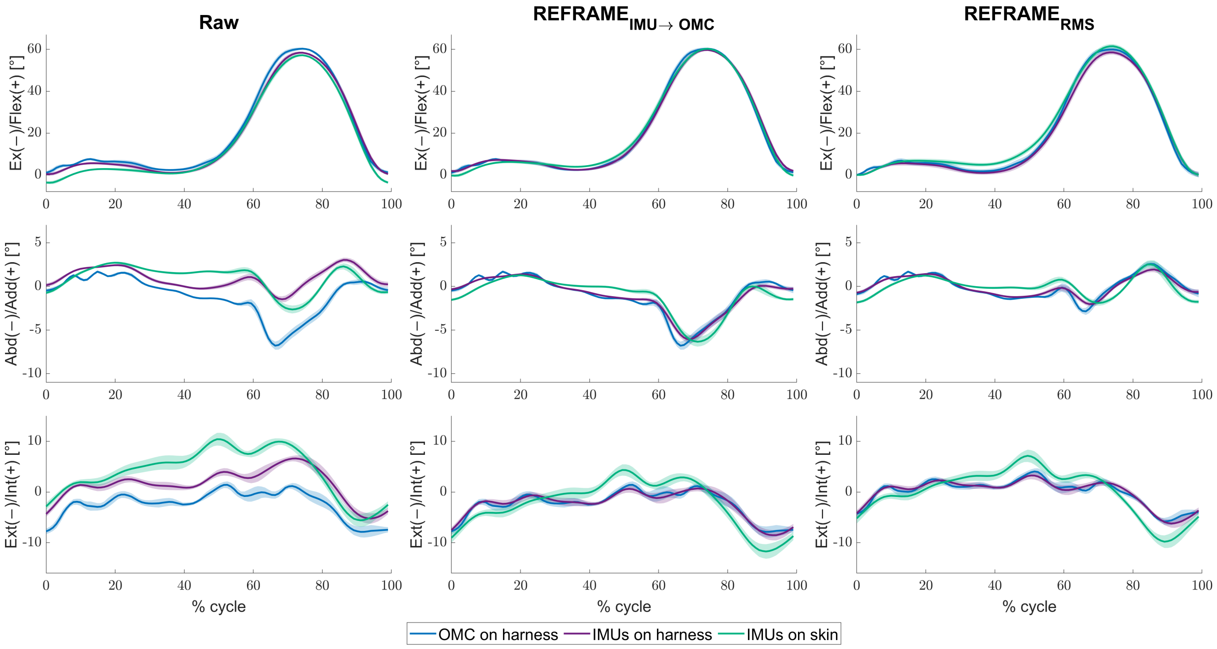

Figure S26: Mean tibio-femoral joint angles (solid lines) ± standard deviation (shaded areas), in degrees, as estimated by inertial measurement units (IMUs) on harness (purple), IMUs on skin (green), and optical motion capture (OMC) on harness (blue), averaged over all cycles for knee 27. Angles are shown as a percentage of the gait cycle under three conditions: 1) raw, i.e. in the absence of post-processing methods to correct reference frame orientation differences (left), 2) after implementation of REFRAME$_{IMU->OMC}$ (middle), and 3) after implementation of REFRAME$_{RMS}$ (right).

	Femur		Tibia	
	IMUs on harness	IMUs on skin	IMUs on harness	IMUs on skin
Rx	0.0 ± 0.0	0.0 ± 0.0	-1.4 ± 0.1	-3.4 ± 0.2
Ry	0.3 ± 0.2	-0.2 ± 0.6	-0.1 ± 0.2	-1.2 ± 0.6
Rz	-5.0 ± 0.1	-2.4 ± 0.3	-8.2 ± 0.1	-8.6 ± 0.4

Table S79: Mean ± standard deviation (in degrees) of the rotational transformations (Rx: Rotation around X, Ry: Rotation around Y, Rz: Rotation around Z) applied to the femoral and tibial segment frames as part of REFRAME$_{IMU->OMC}$, calculated across all cycles of knee 27.

	Femur			Tibia		
	OMC on harness	IMUs on harness	IMUs on skin	OMC on harness	IMUs on harness	IMUs on skin
Rx	0.0 ± 0.0	0.0 ± 0.0	0.0 ± 0.0	0.5 ± 0.8	0.1 ± 0.7	-3.8 ± 0.7
Ry	4.8 ± 0.7	4.7 ± 0.8	5.2 ± 0.8	4.5 ± 0.7	3.8 ± 0.8	3.9 ± 0.8
Rz	2.9 ± 0.4	-2.0 ± 0.4	0.1 ± 0.5	6.4 ± 0.4	-1.9 ± 0.4	-2.2 ± 0.4

Table S80: Mean ± standard deviation (in degrees) of the rotational transformations (Rx: Rotation around X, Ry: Rotation around Y, Rz: Rotation around Z) applied to the femoral and tibial segment frames as part of REFRAME$_{RMS}$, calculated across all cycles of knee 27.

	**Raw**		**REFRAME$_{IMU->OMC}$**		**REFRAME$_{RMS}$**	
	IMUs on harness	IMUs on skin	IMUs on harness	IMUs on skin	IMUs on harness	IMUs on skin
flexion/extension	1.9 ± 0.1	3.5 ± 0.1	1.3 ± 0.1	1.5 ± 0.1	1.6 ± 0.1	2.1 ± 0.2
abduction/adduction	2.6 ± 0.1	2.5 ± 0.1	0.4 ± 0.0	1.0 ± 0.1	0.4 ± 0.0	1.0 ± 0.1
external/internal rotation	4.0 ± 0.1	6.8 ± 0.3	0.5 ± 0.0	2.4 ± 0.4	0.5 ± 0.0	2.4 ± 0.4

Table S81: Mean ± standard deviation (in degrees) root-mean-square errors between tibio-femoral joint rotations estimated by the IMU-based systems and the optical reference on harness, calculated across all trials of knee 27.

2.27 Averaged plots for individual knee 28

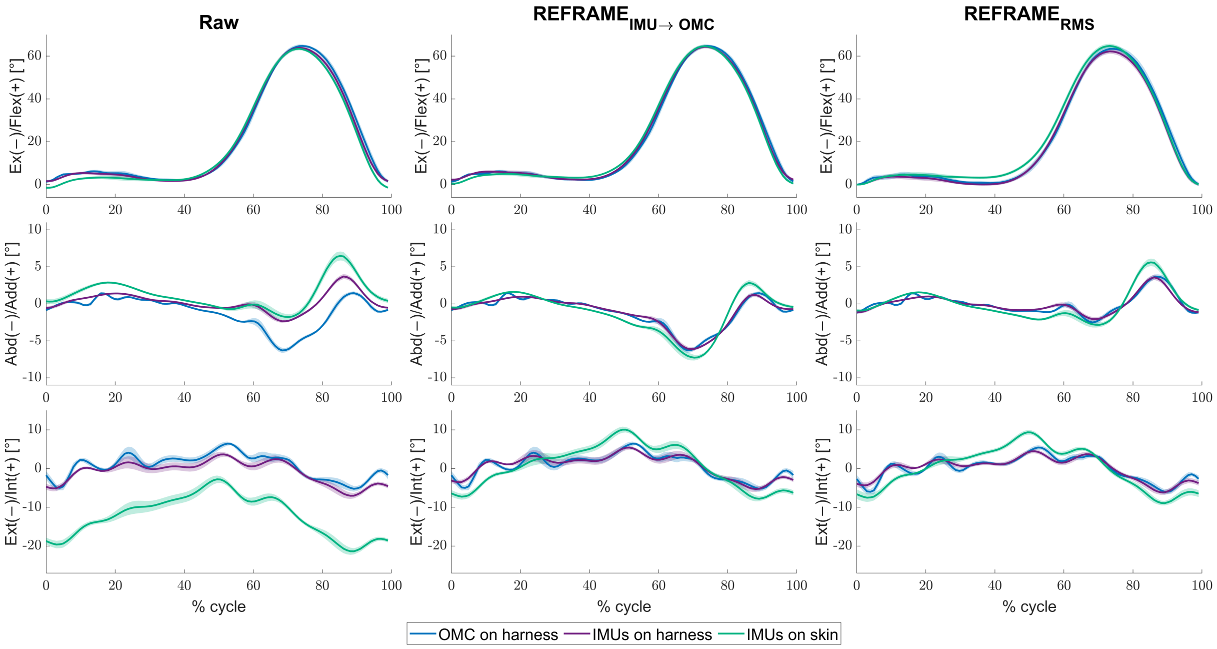

Figure S27: Mean tibio-femoral joint angles (solid lines) ± standard deviation (shaded areas), in degrees, as estimated by inertial measurement units (IMUs) on harness (purple), IMUs on skin (green), and optical motion capture (OMC) on harness (blue), averaged over all cycles for knee 28. Angles are shown as a percentage of the gait cycle under three conditions: 1) raw, i.e. in the absence of post-processing methods to correct reference frame orientation differences (left), 2) after implementation of REFRAME$_{IMU->OMC}$ (middle), and 3) after implementation of REFRAME$_{RMS}$ (right).

	Femur		Tibia	
	IMUs on harness	IMUs on skin	IMUs on harness	IMUs on skin
Rx	0.0 ± 0.0	0.0 ± 0.0	-0.6 ± 0.1	-0.6 ± 0.1
Ry	-1.3 ± 0.2	-3.0 ± 0.6	-1.4 ± 0.2	-4.3 ± 0.5
Rz	-3.4 ± 0.2	-3.1 ± 0.6	-1.8 ± 0.1	9.2 ± 0.3

Table S82: Mean ± standard deviation (in degrees) of the rotational transformations (Rx: Rotation around X, Ry: Rotation around Y, Rz: Rotation around Z) applied to the femoral and tibial segment frames as part of $REFRAME_{IMU->OMC}$, calculated across all cycles of knee 28.

	Femur			Tibia		
	OMC on harness	IMUs on harness	IMUs on skin	OMC on harness	IMUs on harness	IMUs on skin
Rx	0.0 ± 0.0	0.0 ± 0.0	0.0 ± 0.0	1.5 ± 0.8	1.6 ± 0.6	-0.9 ± 0.5
Ry	0.8 ± 0.5	-0.1 ± 0.4	-0.2 ± 0.6	0.4 ± 0.5	-0.6 ± 0.5	-1.9 ± 0.6
Rz	4.3 ± 0.3	0.9 ± 0.4	0.6 ± 0.6	3.3 ± 0.4	1.7 ± 0.4	12.7 ± 0.5

Table S83: Mean ± standard deviation (in degrees) of the rotational transformations (Rx: Rotation around X, Ry: Rotation around Y, Rz: Rotation around Z) applied to the femoral and tibial segment frames as part of $REFRAME_{RMS}$, calculated across all cycles of knee 28.

	**Raw**		**$REFRAME_{IMU->OMC}$**		**$REFRAME_{RMS}$**	
	IMUs on harness	IMUs on skin	IMUs on harness	IMUs on skin	IMUs on harness	IMUs on skin
flexion/extension	1.5 ± 0.2	2.9 ± 0.2	1.3 ± 0.2	2.1 ± 0.2	1.5 ± 0.4	2.7 ± 0.2
abduction/adduction	1.9 ± 0.1	2.9 ± 0.2	0.3 ± 0.1	1.0 ± 0.1	0.4 ± 0.1	0.9 ± 0.1
external/internal rotation	1.8 ± 0.1	13.2 ± 0.4	0.9 ± 0.1	2.9 ± 0.2	0.9 ± 0.1	2.9 ± 0.2

Table S84: Mean ± standard deviation (in degrees) root-mean-square errors between tibio-femoral joint rotations estimated by the IMU-based systems and the optical reference on harness, calculated across all trials of knee 28.

2.28 Averaged plots for individual knee 29

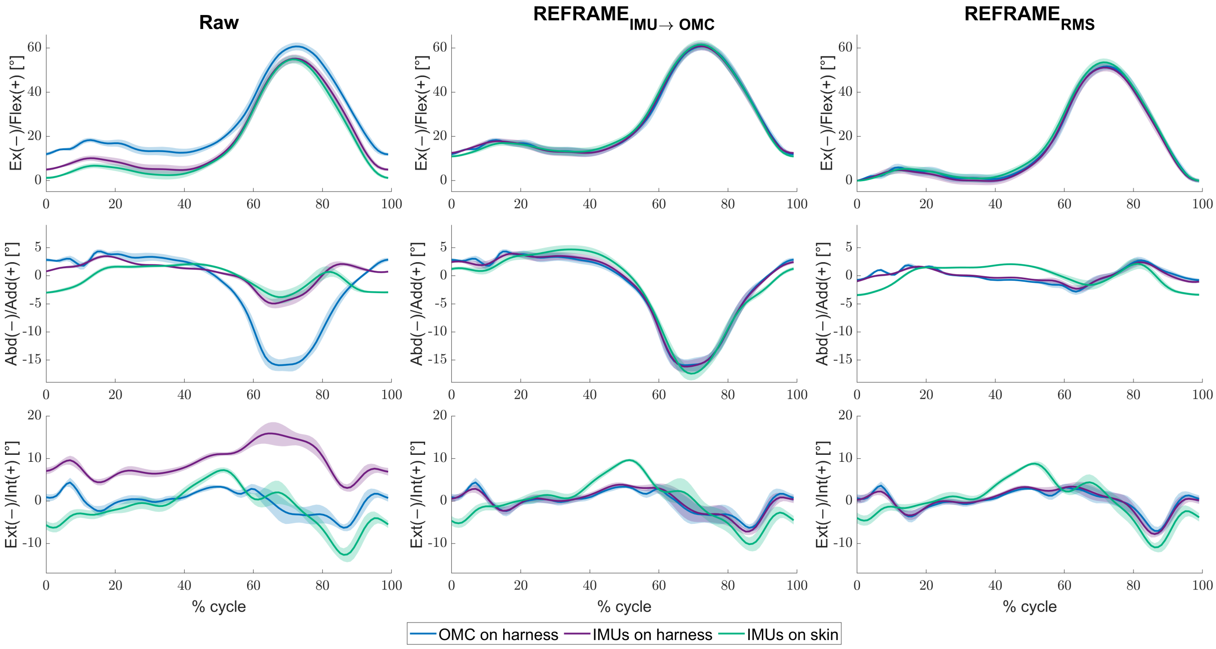

Figure S28: Mean tibio-femoral joint angles (solid lines) ± standard deviation (shaded areas), in degrees, as estimated by inertial measurement units (IMUs) on harness (purple), IMUs on skin (green), and optical motion capture (OMC) on harness (blue), averaged over all cycles for knee 29. Angles are shown as a percentage of the gait cycle under three conditions: 1) raw, i.e. in the absence of post-processing methods to correct reference frame orientation differences (left), 2) after implementation of REFRAME$_{IMU->OMC}$ (middle), and 3) after implementation of REFRAME$_{RMS}$ (right).

	Femur		Tibia	
	IMUs on harness	IMUs on skin	IMUs on harness	IMUs on skin
Rx	0.0 ± 0.0	0.0 ± 0.0	-5.1 ± 0.0	-8.6 ± 0.2
Ry	1.1 ± 0.3	-10.1 ± 1.7	7.4 ± 0.3	-3.5 ± 1.8
Rz	-19.5 ± 0.2	-17.0 ± 0.7	-25.6 ± 0.1	-17.1 ± 0.7

Table S85: Mean ± standard deviation (in degrees) of the rotational transformations (Rx: Rotation around X, Ry: Rotation around Y, Rz: Rotation around Z) applied to the femoral and tibial segment frames as part of REFRAME$_{IMU->OMC}$, calculated across all cycles of knee 29.

	Femur			Tibia		
	OMC on harness	IMUs on harness	IMUs on skin	OMC on harness	IMUs on harness	IMUs on skin
Rx	0.0 ± 0.0	0.0 ± 0.0	0.0 ± 0.0	12.2 ± 0.8	6.0 ± 0.6	1.4 ± 0.7
Ry	9.5 ± 1.7	10.1 ± 1.5	0.4 ± 1.7	5.8 ± 1.7	7.7 ± 1.5	0.2 ± 1.6
Rz	20.8 ± 1.0	1.5 ± 0.9	3.0 ± 1.0	21.1 ± 1.1	-5.3 ± 1.0	4.5 ± 1.1

Table S86: Mean ± standard deviation (in degrees) of the rotational transformations (Rx: Rotation around X, Ry: Rotation around Y, Rz: Rotation around Z) applied to the femoral and tibial segment frames as part of REFRAME$_{RMS}$, calculated across all cycles of knee 29.

	**Raw**		**REFRAME$_{IMU->OMC}$**		**REFRAME$_{RMS}$**	
	IMUs on harness	IMUs on skin	IMUs on harness	IMUs on skin	IMUs on harness	IMUs on skin
flexion/extension	7.1 ± 0.1	9.4 ± 0.2	0.8 ± 0.1	1.3 ± 0.2	1.0 ± 0.2	1.4 ± 0.3
abduction/adduction	5.5 ± 0.2	6.3 ± 0.3	0.4 ± 0.0	1.4 ± 0.1	0.4 ± 0.0	2.0 ± 0.1
external/internal rotation	10.0 ± 0.2	4.1 ± 0.3	0.7 ± 0.0	3.7 ± 0.2	0.7 ± 0.1	3.4 ± 0.2

Table S87: Mean ± standard deviation (in degrees) root-mean-square errors between tibio-femoral joint rotations estimated by the IMU-based systems and the optical reference on harness, calculated across all trials of knee 29.

2.29 Averaged plots for individual knee 30

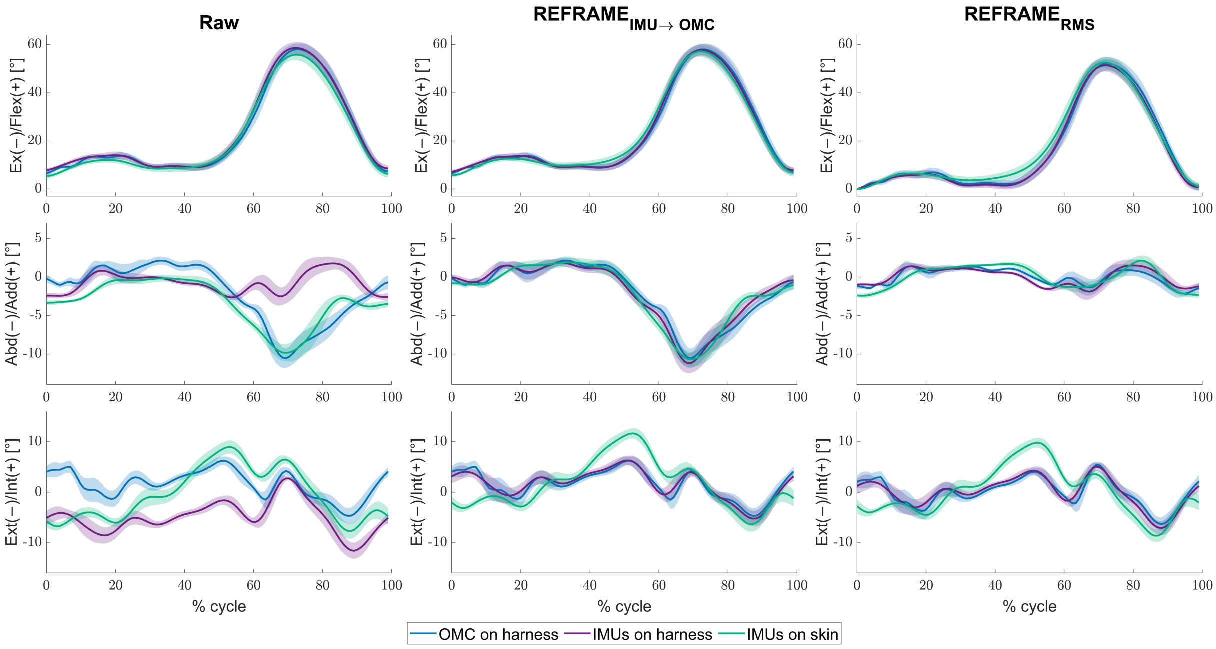

Figure S29: Mean tibio-femoral joint angles (solid lines) ± standard deviation (shaded areas), in degrees, as estimated by inertial measurement units (IMUs) on harness (purple), IMUs on skin (green), and optical motion capture (OMC) on harness (blue), averaged over all cycles for knee 30. Angles are shown as a percentage of the gait cycle under three conditions: 1) raw, i.e. in the absence of post-processing methods to correct reference frame orientation differences (left), 2) after implementation of REFRAME$_{IMU->OMC}$ (middle), and 3) after implementation of REFRAME$_{RMS}$ (right).

	Femur		Tibia	
	IMUs on harness	IMUs on skin	IMUs on harness	IMUs on skin
Rx	0.0 ± 0.0	0.0 ± 0.0	0.5 ± 0.1	-1.1 ± 0.2
Ry	-0.1 ± 0.2	3.6 ± 0.8	4.3 ± 0.2	6.6 ± 0.9
Rz	-15.9 ± 0.1	-6.4 ± 0.4	-7.6 ± 0.1	-2.3 ± 0.4

Table S88: Mean ± standard deviation (in degrees) of the rotational transformations (Rx: Rotation around X, Ry: Rotation around Y, Rz: Rotation around Z) applied to the femoral and tibial segment frames as part of REFRAME$_{IMU->OMC}$, calculated across all cycles of knee 30.

	Femur			Tibia		
	OMC on harness	IMUs on harness	IMUs on skin	OMC on harness	IMUs on harness	IMUs on skin
Rx	0.0 ± 0.0	0.0 ± 0.0	0.0 ± 0.0	6.9 ± 1.0	7.3 ± 0.8	4.5 ± 1.1
Ry	3.0 ± 1.0	3.3 ± 1.1	10.0 ± 1.0	1.6 ± 1.1	5.4 ± 1.0	11.4 ± 1.0
Rz	12.2 ± 0.6	-3.8 ± 0.6	4.2 ± 0.7	10.2 ± 0.6	2.2 ± 0.7	6.8 ± 0.9

Table S89: Mean ± standard deviation (in degrees) of the rotational transformations (Rx: Rotation around X, Ry: Rotation around Y, Rz: Rotation around Z) applied to the femoral and tibial segment frames as part of REFRAME$_{RMS}$, calculated across all cycles of knee 30.

	**Raw**		**REFRAME$_{IMU->OMC}$**		**REFRAME$_{RMS}$**	
	IMUs on harness	IMUs on skin	IMUs on harness	IMUs on skin	IMUs on harness	IMUs on skin
flexion/extension	1.3 ± 0.2	2.0 ± 0.2	1.0 ± 0.2	2.0 ± 0.3	1.3 ± 0.3	2.7 ± 0.3
abduction/adduction	4.3 ± 0.1	2.0 ± 0.1	0.7 ± 0.1	1.1 ± 0.2	0.5 ± 0.0	0.8 ± 0.1
external/internal rotation	7.0 ± 0.2	4.9 ± 0.3	0.8 ± 0.1	3.7 ± 0.4	0.8 ± 0.1	3.6 ± 0.3

Table S90: Mean ± standard deviation (in degrees) root-mean-square errors between tibio-femoral joint rotations estimated by the IMU-based systems and the optical reference on harness, calculated across all trials of knee 30.

2.30 Averaged plots for individual knee 31

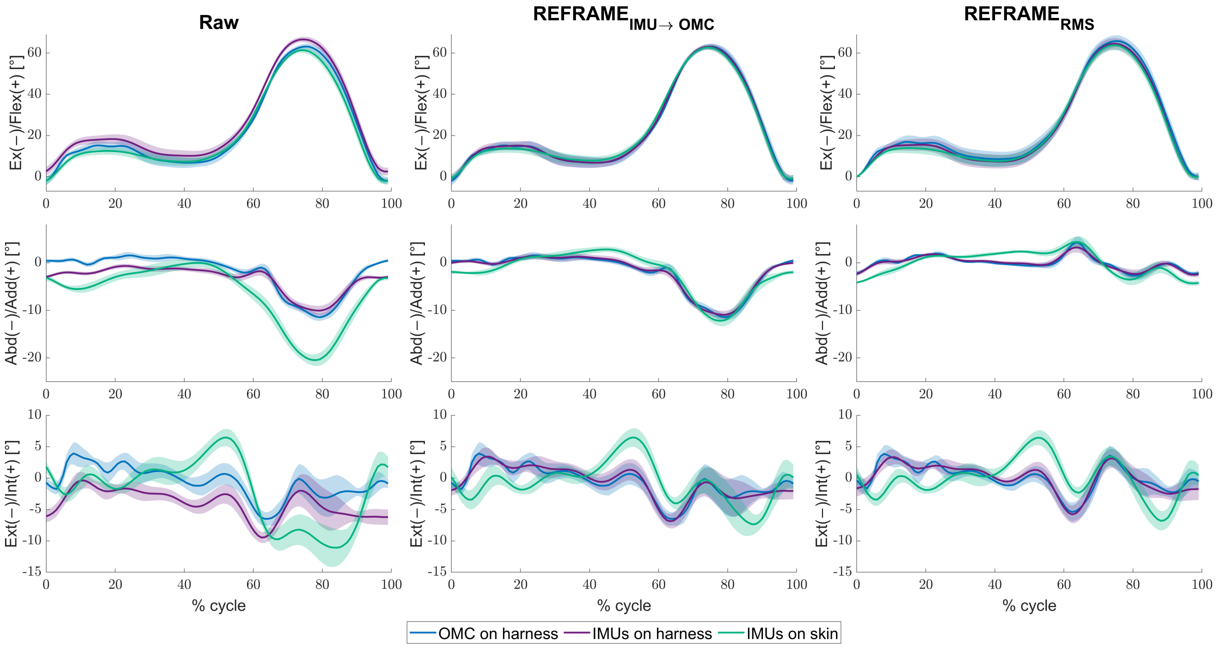

Figure S30: Mean tibio-femoral joint angles (solid lines) ± standard deviation (shaded areas), in degrees, as estimated by inertial measurement units (IMUs) on harness (purple), IMUs on skin (green), and optical motion capture (OMC) on harness (blue), averaged over all cycles for knee 31. Angles are shown as a percentage of the gait cycle under three conditions: 1) raw, i.e. in the absence of post-processing methods to correct reference frame orientation differences (left), 2) after implementation of REFRAME$_{IMU->OMC}$ (middle), and 3) after implementation of REFRAME$_{RMS}$ (right).

	Femur		Tibia	
	IMUs on harness	IMUs on skin	IMUs on harness	IMUs on skin
Rx	0.0 ± 0.0	0.0 ± 0.0	3.1 ± 0.1	-1.0 ± 0.3
Ry	0.6 ± 0.4	-4.9 ± 1.1	3.8 ± 0.4	-3.5 ± 1.1
Rz	-4.8 ± 0.4	10.5 ± 1.1	-0.9 ± 0.2	9.0 ± 0.9

Table S91: Mean ± standard deviation (in degrees) of the rotational transformations (Rx: Rotation around X, Ry: Rotation around Y, Rz: Rotation around Z) applied to the femoral and tibial segment frames as part of $\text{REFRAME}_{IMU->OMC}$, calculated across all cycles of knee 31.

	Femur			Tibia		
	OMC on harness	IMUs on harness	IMUs on skin	OMC on harness	IMUs on harness	IMUs on skin
Rx	0.0 ± 0.0	0.0 ± 0.0	0.0 ± 0.0	-1.6 ± 2.1	2.5 ± 2.0	-0.6 ± 1.8
Ry	2.9 ± 2.0	3.4 ± 2.2	-2.0 ± 2.2	0.3 ± 2.3	4.3 ± 2.5	-2.7 ± 2.5
Rz	11.0 ± 1.1	6.1 ± 1.2	21.2 ± 1.2	11.3 ± 1.0	10.5 ± 0.9	20.1 ± 1.2

Table S92: Mean ± standard deviation (in degrees) of the rotational transformations (Rx: Rotation around X, Ry: Rotation around Y, Rz: Rotation around Z) applied to the femoral and tibial segment frames as part of REFRAME_{RMS}, calculated across all cycles of knee 31.

	**Raw**		**REFRAME**$_{IMU->OMC}$		**REFRAME**$_{RMS}$	
	IMUs on harness	IMUs on skin	IMUs on harness	IMUs on skin	IMUs on harness	IMUs on skin
flexion/extension	3.6 ± 0.1	2.4 ± 0.2	1.0 ± 0.1	1.7 ± 0.2	1.6 ± 0.3	2.3 ± 0.5
abduction/adduction	2.0 ± 0.1	5.3 ± 0.4	0.4 ± 0.1	1.6 ± 0.2	0.4 ± 0.0	1.8 ± 0.1
external/internal rotation	3.5 ± 0.4	4.9 ± 0.4	0.7 ± 0.1	3.5 ± 0.4	0.7 ± 0.1	3.5 ± 0.3

Table S93: Mean ± standard deviation (in degrees) root-mean-square errors between tibio-femoral joint rotations estimated by the IMU-based systems and the optical reference on harness, calculated across all trials of knee 31.

2.31 Averaged plots for individual knee 32

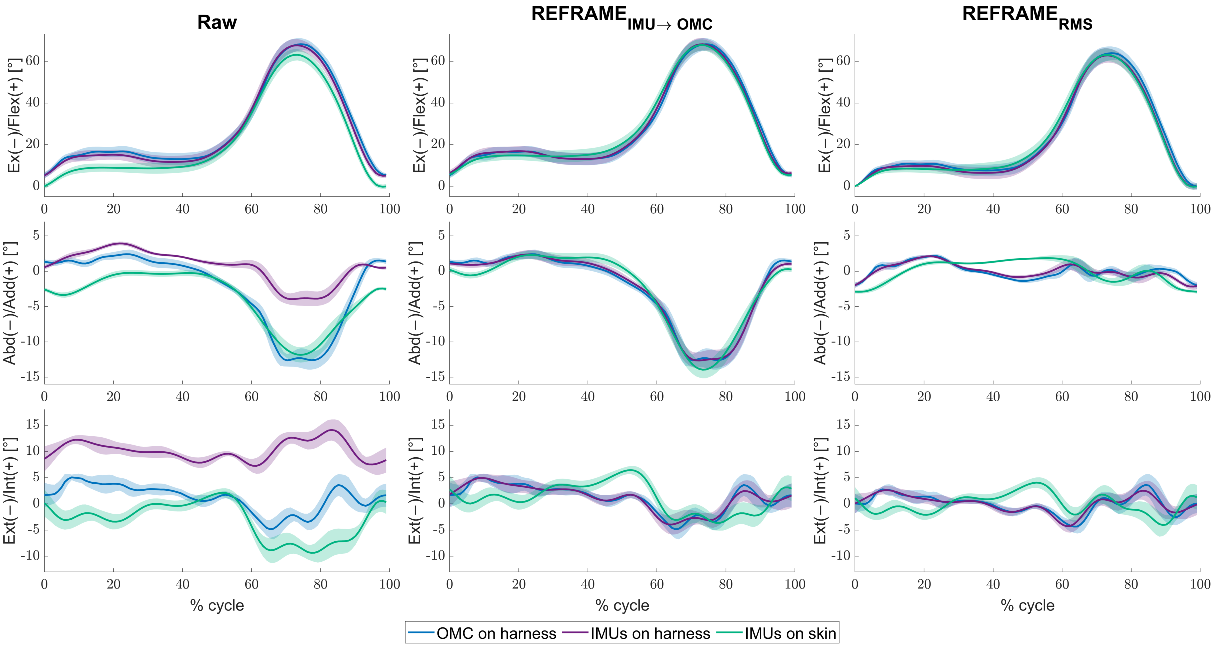

Figure S31: Mean tibio-femoral joint angles (solid lines) ± standard deviation (shaded areas), in degrees, as estimated by inertial measurement units (IMUs) on harness (purple), IMUs on skin (green), and optical motion capture (OMC) on harness (blue), averaged over all cycles for knee 32. Angles are shown as a percentage of the gait cycle under three conditions: 1) raw, i.e. in the absence of post-processing methods to correct reference frame orientation differences (left), 2) after implementation of REFRAME$_{IMU->OMC}$ (middle), and 3) after implementation of REFRAME$_{RMS}$ (right).

	Femur		Tibia	
	IMUs on harness	IMUs on skin	IMUs on harness	IMUs on skin
Rx	0.0 ± 0.0	0.0 ± 0.0	-0.4 ± 0.1	-5.7 ± 0.3
Ry	1.2 ± 0.2	-6.4 ± 1.2	3.1 ± 0.2	-3.3 ± 1.4
Rz	-12.1 ± 0.3	-2.2 ± 1.1	-18.7 ± 0.2	0.1 ± 0.6

Table S94: Mean ± standard deviation (in degrees) of the rotational transformations (Rx: Rotation around X, Ry: Rotation around Y, Rz: Rotation around Z) applied to the femoral and tibial segment frames as part of REFRAME$_{IMU->OMC}$, calculated across all cycles of knee 32.

	Femur			Tibia		
	OMC on harness	IMUs on harness	IMUs on skin	OMC on harness	IMUs on harness	IMUs on skin
Rx	0.0 ± 0.0	0.0 ± 0.0	0.0 ± 0.0	5.8 ± 1.4	5.7 ± 1.4	0.6 ± 1.1
Ry	5.1 ± 1.5	6.0 ± 1.4	-1.6 ± 2.1	1.6 ± 1.5	2.5 ± 1.4	-1.9 ± 2.2
Rz	14.9 ± 0.6	3.1 ± 0.7	13.0 ± 1.2	13.4 ± 0.6	-5.4 ± 0.6	14.0 ± 0.7

Table S95: Mean ± standard deviation (in degrees) of the rotational transformations (Rx: Rotation around X, Ry: Rotation around Y, Rz: Rotation around Z) applied to the femoral and tibial segment frames as part of REFRAME$_{RMS}$, calculated across all cycles of knee 32.

	**Raw**		**REFRAME$_{IMU->OMC}$**		**REFRAME$_{RMS}$**	
	IMUs on harness	IMUs on skin	IMUs on harness	IMUs on skin	IMUs on harness	IMUs on skin
flexion/extension	1.8 ± 0.3	6.1 ± 0.3	1.4 ± 0.3	2.7 ± 0.2	1.8 ± 0.4	2.6 ± 0.4
abduction/adduction	4.4 ± 0.2	2.5 ± 0.2	0.4 ± 0.1	1.2 ± 0.1	0.5 ± 0.1	1.7 ± 0.1
external/internal rotation	9.5 ± 0.2	5.3 ± 0.4	0.7 ± 0.1	3.0 ± 0.3	0.7 ± 0.1	3.0 ± 0.3

Table S96: Mean ± standard deviation (in degrees) root-mean-square errors between tibio-femoral joint rotations estimated by the IMU-based systems and the optical reference on harness, calculated across all trials of knee 32.

2.32 Averaged plots for individual knee 33

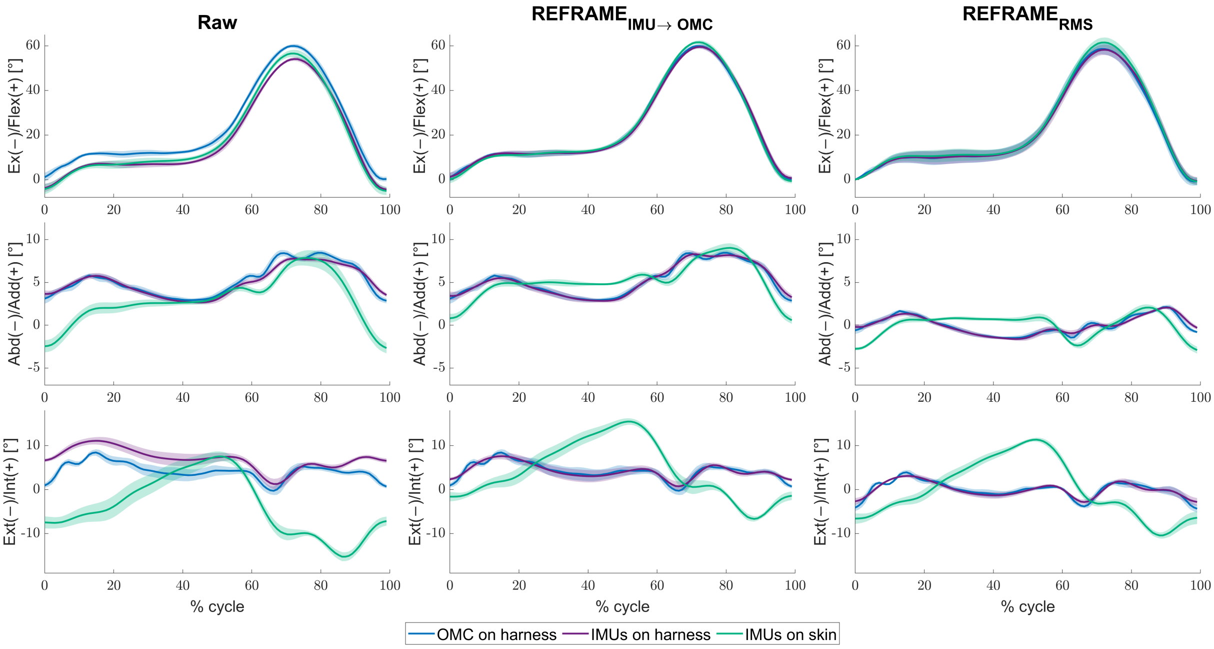

Figure S32: Mean tibio-femoral joint angles (solid lines) ± standard deviation (shaded areas), in degrees, as estimated by inertial measurement units (IMUs) on harness (purple), IMUs on skin (green), and optical motion capture (OMC) on harness (blue), averaged over all cycles for knee 33. Angles are shown as a percentage of the gait cycle under three conditions: 1) raw, i.e. in the absence of post-processing methods to correct reference frame orientation differences (left), 2) after implementation of REFRAME$_{IMU->OMC}$ (middle), and 3) after implementation of REFRAME$_{RMS}$ (right).

	Femur		Tibia	
	IMUs on harness	IMUs on skin	IMUs on harness	IMUs on skin
Rx	0.0 ± 0.0	0.0 ± 0.0	-5.5 ± 0.1	-4.1 ± 1.3
Ry	-3.1 ± 0.2	-5.0 ± 1.0	-2.8 ± 0.3	-2.2 ± 0.9
Rz	2.4 ± 0.2	-0.3 ± 0.6	-2.3 ± 0.1	5.7 ± 0.8

Table S97: Mean ± standard deviation (in degrees) of the rotational transformations (Rx: Rotation around X, Ry: Rotation around Y, Rz: Rotation around Z) applied to the femoral and tibial segment frames as part of $\text{REFRAME}_{IMU->OMC}$, calculated across all cycles of knee 33.

	Femur			Tibia		
	OMC on harness	IMUs on harness	IMUs on skin	OMC on harness	IMUs on harness	IMUs on skin
Rx	0.0 ± 0.0	0.0 ± 0.0	0.0 ± 0.0	0.9 ± 2.0	-5.0 ± 2.0	-3.2 ± 2.5
Ry	-3.6 ± 0.8	-6.7 ± 1.0	-8.4 ± 1.4	-7.3 ± 0.7	-10.1 ± 0.8	-9.2 ± 1.2
Rz	-3.3 ± 0.4	-1.1 ± 0.5	-3.9 ± 0.5	-8.3 ± 0.6	-10.7 ± 0.7	-2.8 ± 1.3

Table S98: Mean ± standard deviation (in degrees) of the rotational transformations (Rx: Rotation around X, Ry: Rotation around Y, Rz: Rotation around Z) applied to the femoral and tibial segment frames as part of REFRAME_{RMS}, calculated across all cycles of knee 33.

	**Raw**		**REFRAME$_{IMU->OMC}$**		**REFRAME$_{RMS}$**	
	IMUs on harness	IMUs on skin	IMUs on harness	IMUs on skin	IMUs on harness	IMUs on skin
flexion/extension	5.2 ± 0.1	4.5 ± 1.3	1.1 ± 0.1	1.1 ± 0.3	1.2 ± 0.2	1.6 ± 0.3
abduction/adduction	0.5 ± 0.1	3.0 ± 0.3	0.3 ± 0.0	1.4 ± 0.1	0.3 ± 0.0	1.5 ± 0.2
external/internal rotation	3.1 ± 0.2	10.7 ± 0.6	0.6 ± 0.1	6.9 ± 0.3	0.6 ± 0.1	6.8 ± 0.2

Table S99: Mean ± standard deviation (in degrees) root-mean-square errors between tibio-femoral joint rotations estimated by the IMU-based systems and the optical reference on harness, calculated across all trials of knee 33.

2.33 Averaged plots for individual knee 34

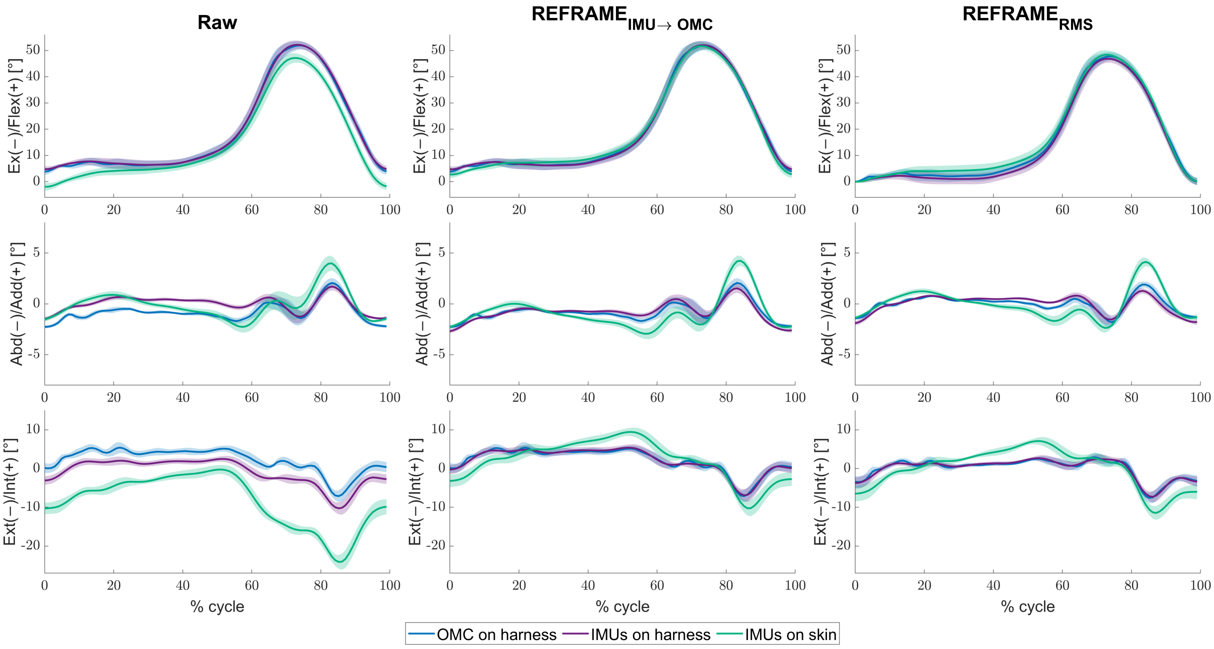

Figure S33: Mean tibio-femoral joint angles (solid lines) ± standard deviation (shaded areas), in degrees, as estimated by inertial measurement units (IMUs) on harness (purple), IMUs on skin (green), and optical motion capture (OMC) on harness (blue), averaged over all cycles for knee 34. Angles are shown as a percentage of the gait cycle under three conditions: 1) raw, i.e. in the absence of post-processing methods to correct reference frame orientation differences (left), 2) after implementation of REFRAME$_{IMU->OMC}$ (middle), and 3) after implementation of REFRAME$_{RMS}$ (right).

	Femur		Tibia	
	IMUs on harness	IMUs on skin	IMUs on harness	IMUs on skin
Rx	0.0 ± 0.0	0.0 ± 0.0	0.2 ± 0.1	-2.2 ± 0.3
Ry	-0.4 ± 0.3	-11.4 ± 1.4	-1.7 ± 0.3	-13.0 ± 1.3
Rz	2.0 ± 0.3	4.1 ± 0.6	4.7 ± 0.1	11.0 ± 0.8

Table S100: Mean ± standard deviation (in degrees) of the rotational transformations (Rx: Rotation around X, Ry: Rotation around Y, Rz: Rotation around Z) applied to the femoral and tibial segment frames as part of REFRAME$_{IMU->OMC}$, calculated across all cycles of knee 34.

	Femur			Tibia		
	OMC on harness	IMUs on harness	IMUs on skin	OMC on harness	IMUs on harness	IMUs on skin
Rx	0.0 ± 0.0	0.0 ± 0.0	0.0 ± 0.0	3.8 ± 1.3	5.2 ± 1.2	1.1 ± 1.6
Ry	-6.1 ± 0.9	-6.7 ± 1.0	-16.2 ± 2.0	-5.2 ± 0.9	-6.9 ± 1.0	-16.1 ± 2.0
Rz	1.0 ± 0.5	3.1 ± 0.5	4.3 ± 0.9	-2.7 ± 0.5	2.3 ± 0.5	8.6 ± 1.0

Table S101: Mean ± standard deviation (in degrees) of the rotational transformations (Rx: Rotation around X, Ry: Rotation around Y, Rz: Rotation around Z) applied to the femoral and tibial segment frames as part of REFRAME$_{RMS}$, calculated across all cycles of knee 34.

	**Raw**		**REFRAME$_{IMU->OMC}$**		**REFRAME$_{RMS}$**	
	IMUs on harness	IMUs on skin	IMUs on harness	IMUs on skin	IMUs on harness	IMUs on skin
flexion/extension	0.6 ± 0.0	4.4 ± 0.1	0.6 ± 0.0	1.2 ± 0.1	1.1 ± 0.1	1.4 ± 0.2
abduction/adduction	1.0 ± 0.1	1.1 ± 0.2	0.3 ± 0.0	1.0 ± 0.1	0.4 ± 0.0	0.9 ± 0.1
external/internal rotation	3.1 ± 0.2	11.4 ± 0.3	0.5 ± 0.1	3.0 ± 0.3	0.5 ± 0.1	3.1 ± 0.3

Table S102: Mean ± standard deviation (in degrees) root-mean-square errors between tibio-femoral joint rotations estimated by the IMU-based systems and the optical reference on harness, calculated across all trials of knee 34.

2.34 Averaged plots for individual knee 35

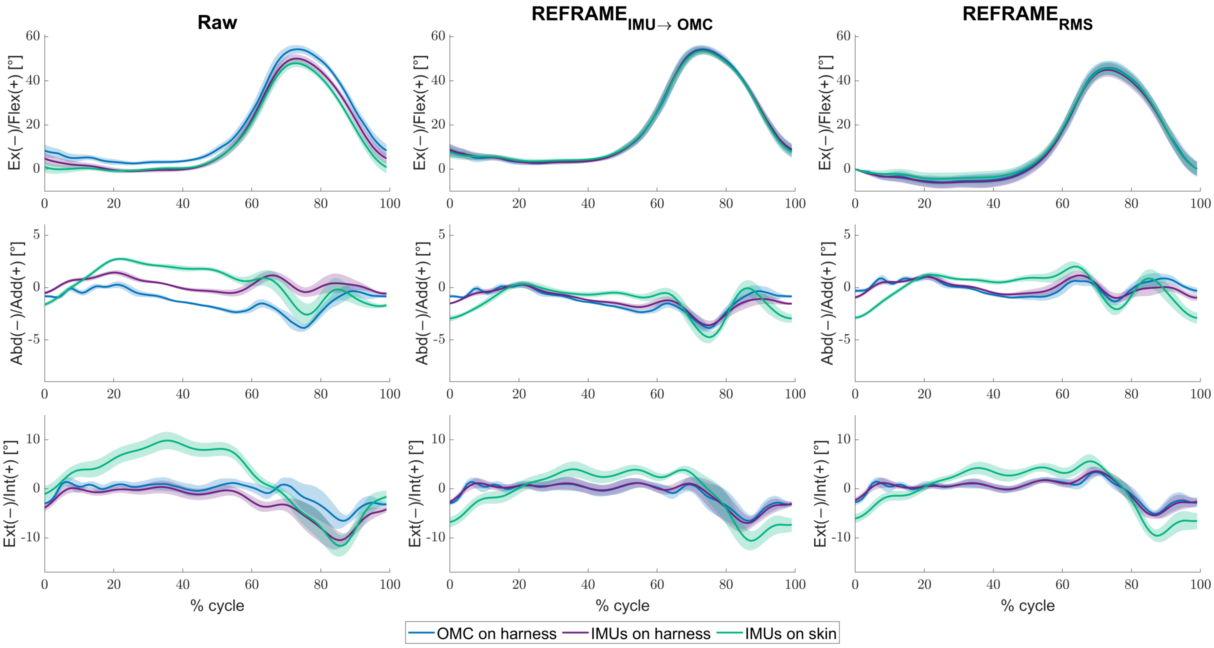

Figure S34: Mean tibio-femoral joint angles (solid lines) ± standard deviation (shaded areas), in degrees, as estimated by inertial measurement units (IMUs) on harness (purple), IMUs on skin (green), and optical motion capture (OMC) on harness (blue), averaged over all cycles for knee 35. Angles are shown as a percentage of the gait cycle under three conditions: 1) raw, i.e. in the absence of post-processing methods to correct reference frame orientation differences (left), 2) after implementation of REFRAME$_{IMU->OMC}$ (middle), and 3) after implementation of REFRAME$_{RMS}$ (right).

	Femur		Tibia	
	IMUs on harness	IMUs on skin	IMUs on harness	IMUs on skin
Rx	0.0 ± 0.0	0.0 ± 0.0	-3.7 ± 0.1	-6.4 ± 0.3
Ry	-5.1 ± 0.2	-12.5 ± 0.8	-6.2 ± 0.1	-14.6 ± 0.9
Rz	-0.5 ± 0.2	5.4 ± 0.8	-0.0 ± 0.2	-1.8 ± 0.8

Table S103: Mean ± standard deviation (in degrees) of the rotational transformations (Rx: Rotation around X, Ry: Rotation around Y, Rz: Rotation around Z) applied to the femoral and tibial segment frames as part of REFRAME$_{IMU->OMC}$, calculated across all cycles of knee 35.

	Femur			Tibia		
	OMC on harness	IMUs on harness	IMUs on skin	OMC on harness	IMUs on harness	IMUs on skin
Rx	0.0 ± 0.0	0.0 ± 0.0	0.0 ± 0.0	8.5 ± 2.9	5.3 ± 2.7	1.7 ± 2.9
Ry	-1.6 ± 2.2	-6.8 ± 2.1	-13.3 ± 2.4	-0.7 ± 2.2	-6.8 ± 2.1	-14.5 ± 2.4
Rz	3.2 ± 1.3	2.8 ± 1.4	8.2 ± 1.9	3.3 ± 1.4	4.3 ± 1.5	3.3 ± 2.1

Table S104: Mean ± standard deviation (in degrees) of the rotational transformations (Rx: Rotation around X, Ry: Rotation around Y, Rz: Rotation around Z) applied to the femoral and tibial segment frames as part of REFRAME$_{RMS}$, calculated across all cycles of knee 35.

	**Raw**		**REFRAME$_{IMU->OMC}$**		**REFRAME$_{RMS}$**	
	IMUs on harness	IMUs on skin	IMUs on harness	IMUs on skin	IMUs on harness	IMUs on skin
flexion/extension	3.9 ± 0.1	5.7 ± 0.2	0.6 ± 0.1	0.9 ± 0.2	0.8 ± 0.2	1.1 ± 0.4
abduction/adduction	1.8 ± 0.2	2.3 ± 0.2	0.4 ± 0.0	1.1 ± 0.1	0.4 ± 0.0	1.4 ± 0.1
external/internal rotation	2.4 ± 0.1	5.5 ± 0.4	0.5 ± 0.1	3.1 ± 0.4	0.5 ± 0.1	3.0 ± 0.4

Table S105: Mean ± standard deviation (in degrees) root-mean-square errors between tibio-femoral joint rotations estimated by the IMU-based systems and the optical reference on harness, calculated across all trials of knee 35.

2.35 Averaged plots for individual knee 36

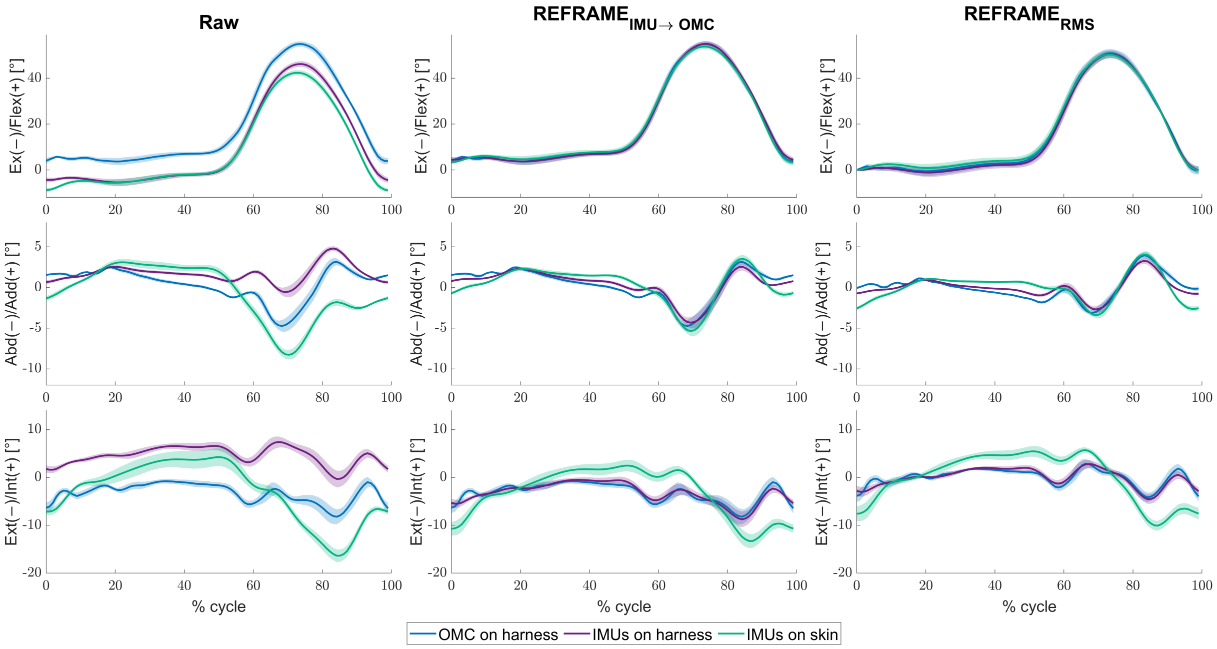

Figure S35: Mean tibio-femoral joint angles (solid lines) ± standard deviation (shaded areas), in degrees, as estimated by inertial measurement units (IMUs) on harness (purple), IMUs on skin (green), and optical motion capture (OMC) on harness (blue), averaged over all cycles for knee 36. Angles are shown as a percentage of the gait cycle under three conditions: 1) raw, i.e. in the absence of post-processing methods to correct reference frame orientation differences (left), 2) after implementation of REFRAME$_{IMU->OMC}$ (middle), and 3) after implementation of REFRAME$_{RMS}$ (right).

	Femur		Tibia	
	IMUs on harness	IMUs on skin	IMUs on harness	IMUs on skin
Rx	0.0 ± 0.0	0.0 ± 0.0	-8.7 ± 0.1	-10.8 ± 0.2
Ry	1.4 ± 0.2	-9.5 ± 1.5	2.2 ± 0.2	-10.8 ± 1.5
Rz	-5.0 ± 0.1	8.4 ± 0.5	-12.1 ± 0.1	4.5 ± 0.6

Table S106: Mean ± standard deviation (in degrees) of the rotational transformations (Rx: Rotation around X, Ry: Rotation around Y, Rz: Rotation around Z) applied to the femoral and tibial segment frames as part of REFRAME$_{IMU->OMC}$, calculated across all cycles of knee 36.

	Femur			Tibia		
	OMC on harness	IMUs on harness	IMUs on skin	OMC on harness	IMUs on harness	IMUs on skin
Rx	0.0 ± 0.0	0.0 ± 0.0	0.0 ± 0.0	4.3 ± 1.3	-4.4 ± 1.1	-6.9 ± 0.7
Ry	-1.5 ± 1.4	-0.3 ± 1.5	-9.2 ± 2.0	-2.6 ± 1.5	-1.5 ± 1.5	-11.3 ± 2.1
Rz	4.7 ± 0.5	-0.3 ± 0.5	12.4 ± 0.8	7.3 ± 0.6	-5.0 ± 0.6	12.3 ± 0.8

Table S107: Mean ± standard deviation (in degrees) of the rotational transformations (Rx: Rotation around X, Ry: Rotation around Y, Rz: Rotation around Z) applied to the femoral and tibial segment frames as part of REFRAME$_{RMS}$, calculated across all cycles of knee 36.

	**Raw**		**REFRAME**$_{IMU->OMC}$		**REFRAME**$_{RMS}$	
	IMUs on harness	IMUs on skin	IMUs on harness	IMUs on skin	IMUs on harness	IMUs on skin
flexion/extension	9.0 ± 0.1	11.0 ± 0.1	0.7 ± 0.1	1.1 ± 0.1	0.9 ± 0.2	1.4 ± 0.4
abduction/adduction	1.9 ± 0.1	2.7 ± 0.2	0.5 ± 0.0	1.1 ± 0.1	0.6 ± 0.0	1.3 ± 0.1
external/internal rotation	7.9 ± 0.1	4.9 ± 0.5	0.7 ± 0.1	4.1 ± 0.4	0.7 ± 0.1	4.1 ± 0.4

Table S108: Mean ± standard deviation (in degrees) root-mean-square errors between tibio-femoral joint rotations estimated by the IMU-based systems and the optical reference on harness, calculated across all trials of knee 36.

2.36 Averaged plots for individual knee 37

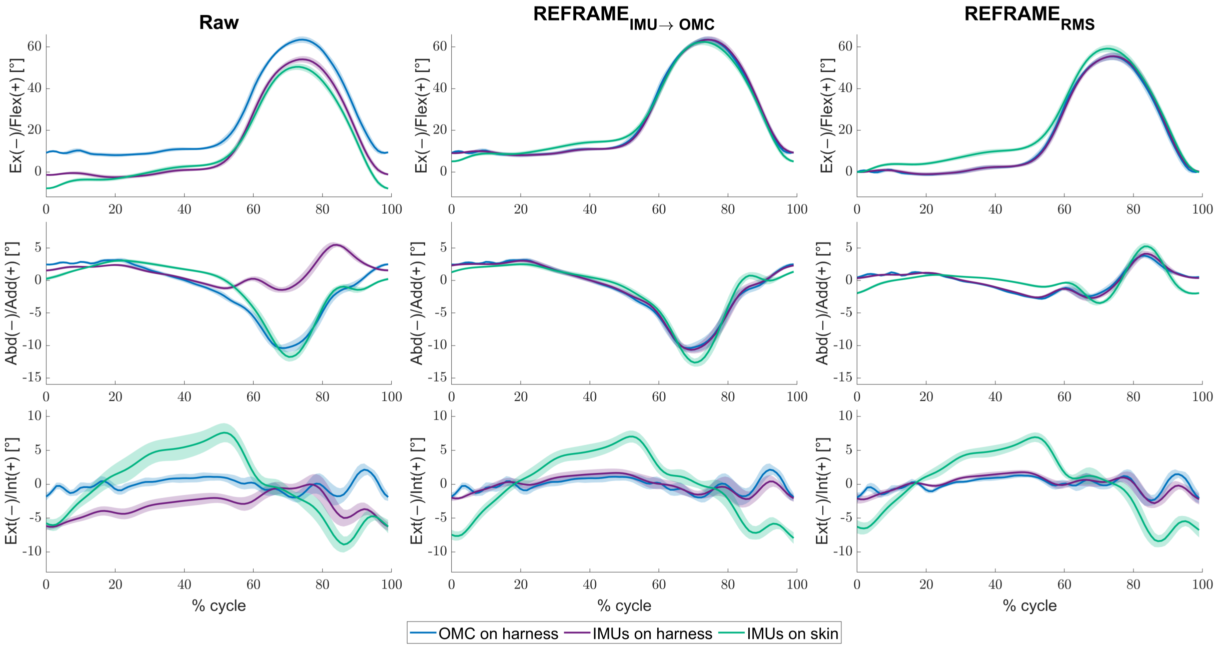

Figure S36: Mean tibio-femoral joint angles (solid lines) ± standard deviation (shaded areas), in degrees, as estimated by inertial measurement units (IMUs) on harness (purple), IMUs on skin (green), and optical motion capture (OMC) on harness (blue), averaged over all cycles for knee 37. Angles are shown as a percentage of the gait cycle under three conditions: 1) raw, i.e. in the absence of post-processing methods to correct reference frame orientation differences (left), 2) after implementation of REFRAME$_{IMU->OMC}$ (middle), and 3) after implementation of REFRAME$_{RMS}$ (right).

	Femur		Tibia	
	IMUs on harness	IMUs on skin	IMUs on harness	IMUs on skin
Rx	0.0 ± 0.0	0.0 ± 0.0	-9.8 ± 0.1	-12.5 ± 0.2
Ry	-1.9 ± 0.2	-5.9 ± 0.9	-0.4 ± 0.1	-6.3 ± 0.9
Rz	-11.3 ± 0.2	2.4 ± 0.8	-7.7 ± 0.2	0.0 ± 0.7

Table S109: Mean ± standard deviation (in degrees) of the rotational transformations (Rx: Rotation around X, Ry: Rotation around Y, Rz: Rotation around Z) applied to the femoral and tibial segment frames as part of $\text{REFRAME}_{IMU->OMC}$, calculated across all cycles of knee 37.

	Femur			Tibia		
	OMC on harness	IMUs on harness	IMUs on skin	OMC on harness	IMUs on harness	IMUs on skin
Rx	0.0 ± 0.0	0.0 ± 0.0	0.0 ± 0.0	9.0 ± 0.4	-1.6 ± 0.4	-7.3 ± 0.4
Ry	5.2 ± 0.9	3.0 ± 1.0	1.5 ± 0.9	3.5 ± 0.9	1.7 ± 1.0	-1.4 ± 0.9
Rz	10.1 ± 0.4	-1.4 ± 0.4	12.0 ± 0.8	10.3 ± 0.6	2.7 ± 0.5	11.4 ± 0.8

Table S110: Mean ± standard deviation (in degrees) of the rotational transformations (Rx: Rotation around X, Ry: Rotation around Y, Rz: Rotation around Z) applied to the femoral and tibial segment frames as part of REFRAME_{RMS}, calculated across all cycles of knee 37.

	**Raw**		**REFRAME**$_{IMU->OMC}$		**REFRAME**$_{RMS}$	
	IMUs on harness	IMUs on skin	IMUs on harness	IMUs on skin	IMUs on harness	IMUs on skin
flexion/extension	10.1 ± 0.1	12.4 ± 0.2	1.0 ± 0.1	2.3 ± 0.1	1.0 ± 0.1	5.0 ± 0.2
abduction/adduction	4.7 ± 0.1	1.5 ± 0.1	0.3 ± 0.0	1.1 ± 0.1	0.3 ± 0.0	1.4 ± 0.1
external/internal rotation	3.6 ± 0.2	4.5 ± 0.3	0.7 ± 0.1	4.3 ± 0.3	0.7 ± 0.1	4.1 ± 0.3

Table S111: Mean ± standard deviation (in degrees) root-mean-square errors between tibio-femoral joint rotations estimated by the IMU-based systems and the optical reference on harness, calculated across all trials of knee 37.

2.37 Averaged plots for individual knee 38

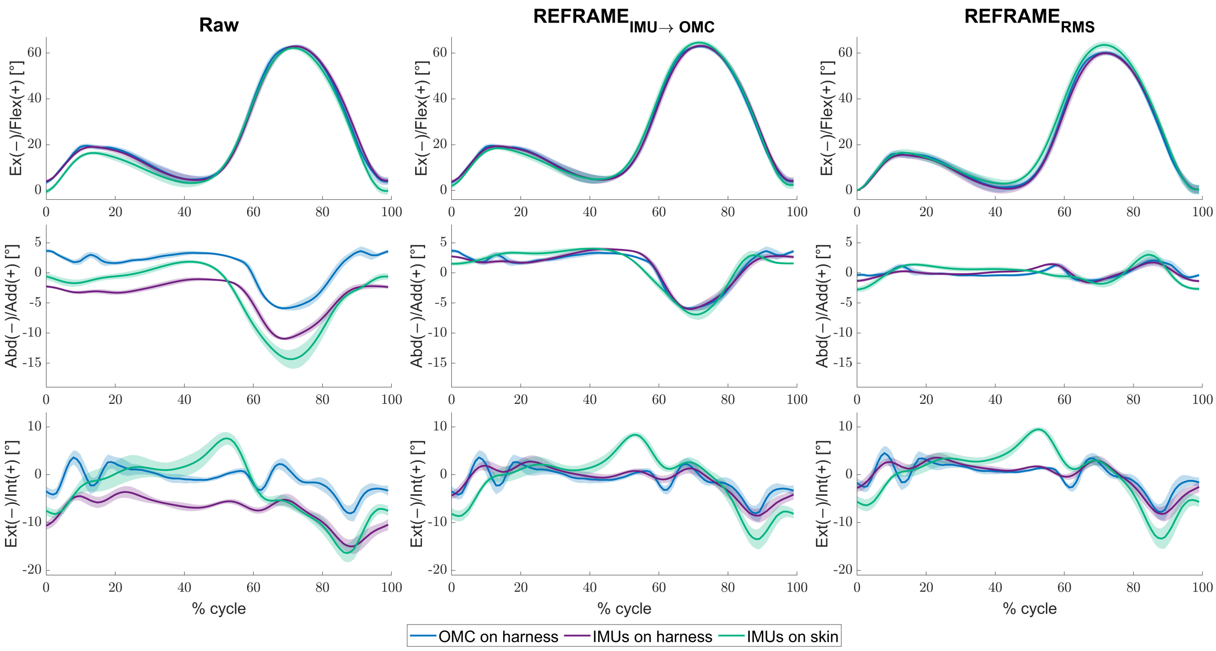

Figure S37: Mean tibio-femoral joint angles (solid lines) ± standard deviation (shaded areas), in degrees, as estimated by inertial measurement units (IMUs) on harness (purple), IMUs on skin (green), and optical motion capture (OMC) on harness (blue), averaged over all cycles for knee 38. Angles are shown as a percentage of the gait cycle under three conditions: 1) raw, i.e. in the absence of post-processing methods to correct reference frame orientation differences (left), 2) after implementation of REFRAME$_{IMU->OMC}$ (middle), and 3) after implementation of REFRAME$_{RMS}$ (right).

	Femur		Tibia	
	IMUs on harness	IMUs on skin	IMUs on harness	IMUs on skin
Rx	0.0 ± 0.0	0.0 ± 0.0	-0.7 ± 0.1	-2.4 ± 0.3
Ry	-0.3 ± 0.3	-4.9 ± 1.0	4.6 ± 0.3	-3.5 ± 0.9
Rz	0.1 ± 0.2	9.4 ± 1.2	6.4 ± 0.2	8.6 ± 0.8

Table S112: Mean ± standard deviation (in degrees) of the rotational transformations (Rx: Rotation around X, Ry: Rotation around Y, Rz: Rotation around Z) applied to the femoral and tibial segment frames as part of $\text{REFRAME}_{IMU->OMC}$, calculated across all cycles of knee 38.

	Femur			Tibia		
	OMC on harness	IMUs on harness	IMUs on skin	OMC on harness	IMUs on harness	IMUs on skin
Rx	0.0 ± 0.0	0.0 ± 0.0	0.0 ± 0.0	3.4 ± 0.9	3.1 ± 0.8	0.2 ± 1.2
Ry	5.1 ± 0.7	4.3 ± 0.6	1.7 ± 0.6	1.3 ± 0.7	5.9 ± 0.6	-0.4 ± 0.7
Rz	6.9 ± 0.6	7.2 ± 0.4	15.4 ± 1.5	8.8 ± 0.5	15.0 ± 0.4	17.2 ± 0.7

Table S113: Mean ± standard deviation (in degrees) of the rotational transformations (Rx: Rotation around X, Ry: Rotation around Y, Rz: Rotation around Z) applied to the femoral and tibial segment frames as part of REFRAME_{RMS}, calculated across all cycles of knee 38.

	**Raw**		**REFRAME**$_{IMU->OMC}$		**REFRAME**$_{RMS}$	
	IMUs on harness	IMUs on skin	IMUs on harness	IMUs on skin	IMUs on harness	IMUs on skin
flexion/extension	1.2 ± 0.1	2.7 ± 0.3	1.0 ± 0.1	1.5 ± 0.1	1.2 ± 0.3	2.3 ± 0.4
abduction/adduction	5.0 ± 0.1	5.1 ± 0.5	0.5 ± 0.1	1.2 ± 0.2	0.5 ± 0.1	1.2 ± 0.2
external/internal rotation	6.6 ± 0.2	5.6 ± 0.3	1.4 ± 0.1	4.3 ± 0.3	1.4 ± 0.1	4.2 ± 0.3

Table S114: Mean ± standard deviation (in degrees) root-mean-square errors between tibio-femoral joint rotations estimated by the IMU-based systems and the optical reference on harness, calculated across all trials of knee 38.

2.38 Averaged plots for individual knee 39

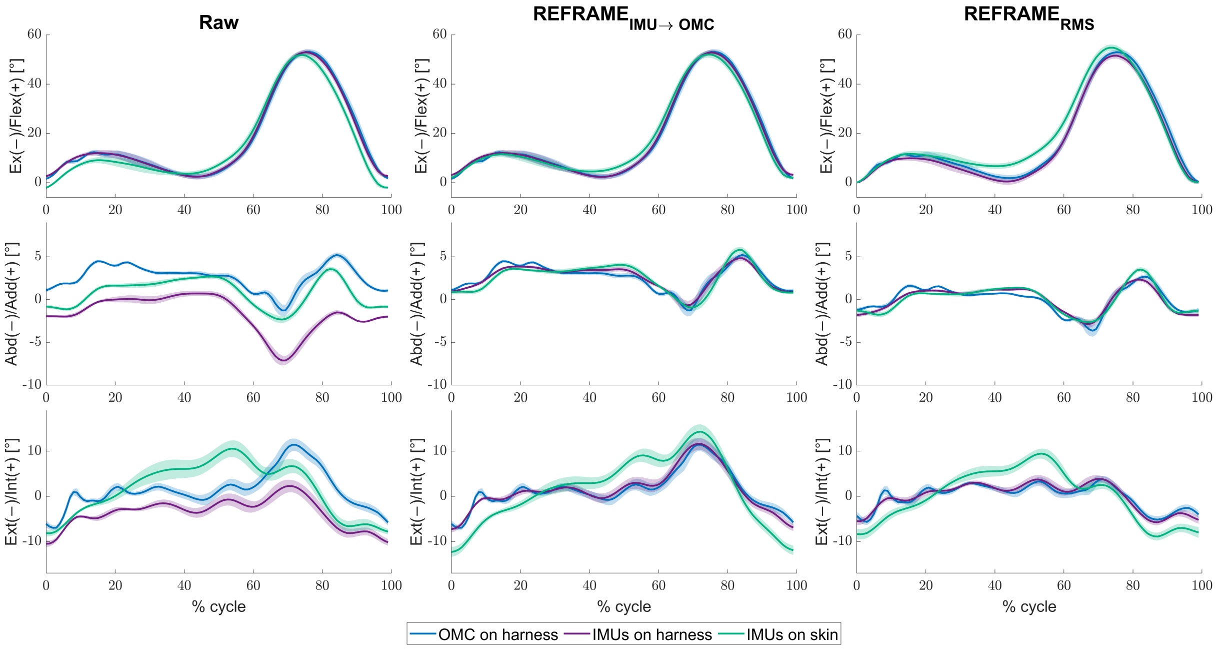

Figure S38: Mean tibio-femoral joint angles (solid lines) ± standard deviation (shaded areas), in degrees, as estimated by inertial measurement units (IMUs) on harness (purple), IMUs on skin (green), and optical motion capture (OMC) on harness (blue), averaged over all cycles for knee 39. Angles are shown as a percentage of the gait cycle under three conditions: 1) raw, i.e. in the absence of post-processing methods to correct reference frame orientation differences (left), 2) after implementation of REFRAME$_{IMU->OMC}$ (middle), and 3) after implementation of REFRAME$_{RMS}$ (right).

	Femur		Tibia	
	IMUs on harness	IMUs on skin	IMUs on harness	IMUs on skin
Rx	0.0 ± 0.0	0.0 ± 0.0	-0.2 ± 0.1	-2.6 ± 0.2
Ry	-4.7 ± 0.3	-12.2 ± 1.2	-2.3 ± 0.2	-11.2 ± 1.2
Rz	7.7 ± 0.1	6.3 ± 0.6	10.7 ± 0.1	1.9 ± 0.6

Table S115: Mean ± standard deviation (in degrees) of the rotational transformations (Rx: Rotation around X, Ry: Rotation around Y, Rz: Rotation around Z) applied to the femoral and tibial segment frames as part of $\text{REFRAME}_{IMU->OMC}$, calculated across all cycles of knee 39.

	Femur			Tibia		
	OMC on harness	IMUs on harness	IMUs on skin	OMC on harness	IMUs on harness	IMUs on skin
Rx	0.0 ± 0.0	0.0 ± 0.0	0.0 ± 0.0	0.6 ± 0.6	1.5 ± 0.5	-2.7 ± 0.5
Ry	10.4 ± 1.5	6.1 ± 1.5	4.5 ± 1.5	8.2 ± 1.5	6.7 ± 1.5	3.7 ± 1.5
Rz	-4.5 ± 0.4	3.1 ± 0.4	-0.9 ± 0.6	-2.6 ± 0.5	8.1 ± 0.6	-1.0 ± 1.0

Table S116: Mean ± standard deviation (in degrees) of the rotational transformations (Rx: Rotation around X, Ry: Rotation around Y, Rz: Rotation around Z) applied to the femoral and tibial segment frames as part of REFRAME_{RMS}, calculated across all cycles of knee 39.

	**Raw**		**REFRAME**$_{IMU->OMC}$		**REFRAME**$_{RMS}$	
	IMUs on harness	IMUs on skin	IMUs on harness	IMUs on skin	IMUs on harness	IMUs on skin
flexion/extension	1.0 ± 0.2	3.8 ± 0.2	1.0 ± 0.2	2.5 ± 0.3	1.6 ± 0.2	3.9 ± 0.2
abduction/adduction	4.5 ± 0.1	2.0 ± 0.1	0.4 ± 0.1	0.8 ± 0.1	0.6 ± 0.1	0.7 ± 0.1
external/internal rotation	5.5 ± 0.1	5.0 ± 0.3	0.9 ± 0.1	4.3 ± 0.3	0.8 ± 0.1	3.7 ± 0.3

Table S117: Mean ± standard deviation (in degrees) root-mean-square errors between tibio-femoral joint rotations estimated by the IMU-based systems and the optical reference on harness, calculated across all trials of knee 39.

2.39 Averaged plots for individual knee 40

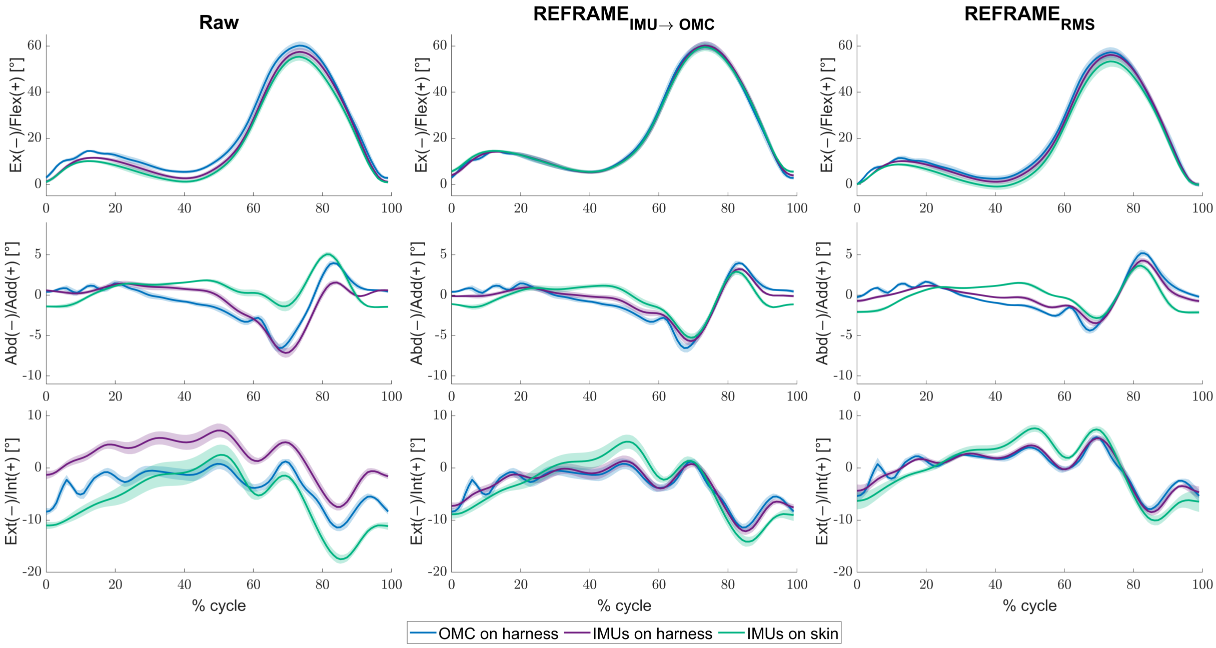

Figure S39: Mean tibio-femoral joint angles (solid lines) ± standard deviation (shaded areas), in degrees, as estimated by inertial measurement units (IMUs) on harness (purple), IMUs on skin (green), and optical motion capture (OMC) on harness (blue), averaged over all cycles for knee 40. Angles are shown as a percentage of the gait cycle under three conditions: 1) raw, i.e. in the absence of post-processing methods to correct reference frame orientation differences (left), 2) after implementation of REFRAME$_{IMU->OMC}$ (middle), and 3) after implementation of REFRAME$_{RMS}$ (right).

	Femur		Tibia	
	IMUs on harness	IMUs on skin	IMUs on harness	IMUs on skin
Rx	0.0 ± 0.0	0.0 ± 0.0	-2.7 ± 0.1	-4.1 ± 0.1
Ry	-0.7 ± 0.3	-2.8 ± 0.7	-1.7 ± 0.3	-3.0 ± 0.8
Rz	3.6 ± 0.3	-3.0 ± 0.6	-2.5 ± 0.1	-1.0 ± 0.3

Table S118: Mean ± standard deviation (in degrees) of the rotational transformations (Rx: Rotation around X, Ry: Rotation around Y, Rz: Rotation around Z) applied to the femoral and tibial segment frames as part of REFRAME$_{IMU->OMC}$, calculated across all cycles of knee 40.

	Femur			Tibia		
	OMC on harness	IMUs on harness	IMUs on skin	OMC on harness	IMUs on harness	IMUs on skin
Rx	0.0 ± 0.0	0.0 ± 0.0	0.0 ± 0.0	3.0 ± 0.8	1.3 ± 0.7	2.1 ± 0.9
Ry	-0.1 ± 1.1	-1.2 ± 1.3	-4.9 ± 2.2	-0.3 ± 1.0	-2.5 ± 1.3	-5.3 ± 2.3
Rz	3.0 ± 0.5	6.6 ± 0.7	1.0 ± 1.4	6.0 ± 0.6	3.6 ± 0.6	5.7 ± 0.7

Table S119: Mean ± standard deviation (in degrees) of the rotational transformations (Rx: Rotation around X, Ry: Rotation around Y, Rz: Rotation around Z) applied to the femoral and tibial segment frames as part of REFRAME$_{RMS}$, calculated across all cycles of knee 40.

	**Raw**		**REFRAME$_{IMU->OMC}$**		**REFRAME$_{RMS}$**	
	IMUs on harness	IMUs on skin	IMUs on harness	IMUs on skin	IMUs on harness	IMUs on skin
flexion/extension	2.8 ± 0.1	4.4 ± 0.1	1.0 ± 0.1	1.2 ± 0.1	1.5 ± 0.3	3.3 ± 0.4
abduction/adduction	1.2 ± 0.0	2.6 ± 0.1	0.7 ± 0.1	1.6 ± 0.0	0.7 ± 0.1	1.9 ± 0.1
external/internal rotation	5.5 ± 0.3	3.9 ± 0.2	0.8 ± 0.1	2.7 ± 0.4	0.8 ± 0.1	2.6 ± 0.3

Table S120: Mean ± standard deviation (in degrees) root-mean-square errors between tibio-femoral joint rotations estimated by the IMU-based systems and the optical reference on harness, calculated across all trials of knee 40.

3 Statistical Analysis

	**Raw vs. REFRAME**$_{IMU->OMC}$	**Raw vs. REFRAME**$_{RMS}$
flexion/extension	$1.3 * 10^{-7}$	$2.9 * 10^{-6}$
abduction/adduction	$9.0 * 10^{-9}$	$1.0 * 10^{-9}$
external/internal rotation	$1.5 * 10^{-12}$	$1.4 * 10^{-12}$

Table S121: p-values associated with a two-tailed paired t-test performed on the mean RMSEs between tibiofemoral joint rotations estimated by the IMUs on harness and the OMC on harness.

	**Raw vs. REFRAME**$_{IMU->OMC}$	**Raw vs. REFRAME**$_{RMS}$
flexion/extension	$1.2 * 10^{-7}$	$4.4 * 10^{-5}$
abduction/adduction	$1.7 * 10^{-9}$	$7.6 * 10^{-9}$
external/internal rotation	$6.7 * 10^{-8}$	$2.0 * 10^{-8}$

Table S122: p-values associated with a two-tailed paired t-test performed on the mean RMSEs between tibiofemoral joint rotations estimated by the IMUs on skin and the OMC on harness.
